# Neural representation of action symbols in primate frontal cortex

**DOI:** 10.1038/s41586-026-10297-x

**Published:** 2026-05-20

**Authors:** Lucas Y. Tian, Kedar Garzón Gupta, Daniel J. Hanuska, Adam G. Rouse, Mark A. G. Eldridge, Marc H. Schieber, Xiao-Jing Wang, Joshua B. Tenenbaum, Winrich A. Freiwald

**Affiliations:** 1 Laboratory of Neural Systems, The Rockefeller University, New York, NY USA; 2 Center for Brains, Minds and Machines, Cambridge, MA USA; 3 The Price Family Center for the Social Brain, The Rockefeller University, New York, NY USA; 4 Department of Neurosurgery, University of Kansas Medical Center, Kansas City, KS USA; 5 Department of Cell Biology and Physiology, University of Kansas Medical Center, Kansas City, KS USA; 6 Biosciences Institute, Newcastle University, Newcastle upon Tyne, UK; 7 Department of Neurology, University of Rochester, Rochester, NY USA; 8 Center for Neural Science, New York University, New York, NY USA; 9 Department of Brain and Cognitive Sciences, Massachusetts Institute of Technology, Cambridge, MA USA

**Keywords:** Problem solving, Intelligence, Decision, Premotor cortex, Decision

## Abstract

A hallmark of intelligence is proficiency in solving new problems, including those that substantially differ from previously seen problems. Problem solving in turn depends on the goal-directed generation of novel ideas and behaviours^1^, which has been proposed to involve internal representations of discrete units (or symbols) that can be recombined into numerous possible composite representations^1–7^. Although this view has been influential in cognitive-level explanations of behaviour, definitive evidence for a neuronal substrate of symbols has remained elusive. Here we identify a neural population that encodes action symbols—recombinable representations of discrete units of motor behaviour—in a specific area of the frontal cortex. In macaque monkeys performing a drawing-like task, we found behavioural evidence that action elements (strokes) exhibit three crucial features that indicate an underlying symbolic representation: (1) invariance over low-level motor parameters; (2) categorical structure, which reflects discrete action types; and (3) recombination into novel sequences. Based on simultaneous neural recordings across eight regions of the motor, premotor and prefrontal cortex, we identified population activity specifically in the ventral premotor cortex that encodes planned actions in a manner that also reflects invariance, categorical structure and recombination. These findings reveal a neural representation of action symbols localized to the ventral premotor cortex and a putative neural substrate for symbolic operations.

## Abstract (web summary)

A drawing-like task designed to study compositional generalization identifies a specific neural population in the ventral premotor cortex in primates that encodes action symbols.

## Main

Understanding the mechanisms of intelligence requires an explanation for generalization, especially to situations or problems that considerably differ from those previously encountered. For example, if asked to draw an animal that does not exist, children can generalize from previous experience to produce an imaginary animal, such as a dog-like creature with six legs, three camel humps and three pig tails^8^. An influential hypothesis for this ability is that such generalization depends on an internal representation of discrete units (symbols) that can be recombined into composite representations in a process called compositional generalization^1–7^. Symbols enable the combinatorial derivation of numerous possible novel representations from a few reused components (for example, animal = 1 torso + 8 arms + 4 legs). This hypothesis is not restricted to concepts explicitly represented as symbol systems in language, computer programs and mathematics but may also be broadly applicable to abilities that are not superficially symbolic^3,5,7^. In humans, these include geometry^5^, handwriting^9^, drawing^10^, dancing^11^, musicianship^5^ and speech^12^. In nonhuman animals, such abilities may include reasoning (logical^7^, spatial^13^, physical^7^, numerical^6^ and social^14^), object manipulation and tool use^15^, artificial grammar learning^16^ and communication^17^.

Despite behaviour implicating the existence of symbolic representations, we lack definitive evidence for whether and how symbols are implemented in neural activity. Furthermore, it is uncertain how symbols reconcile with other mechanistic theories of cognition, including those based on distributed processing in neural networks^18,19^, dynamical systems^20^ and cognitive maps^21,22^. Given that symbols are discrete representational units that are recombined, a neural population that represents symbols should exhibit at least three essential properties: (1) invariance, (2) categorical structure and (3) recombination. Invariance means that activity is independent of variables irrelevant to the task goal. Categorical structure means that there is expression of one distinct activity pattern per symbol and a bias towards these discrete patterns even with continuous variations in task parameters. Recombination implies that the activity pattern of a symbol should reoccur in all contexts in which it is composed with other symbols.

Neural recordings during cognitive tasks have revealed a diversity of invariant representations, including of rules^23,24^, actions^25,26^, sequences^27,28^, numerical concepts^6^, perceptual categories^29^, cognitive maps^22^ and other high-level concepts^30^. Moreover, specific brain regions have been associated with such representations, including the prefrontal cortex (PFC)^23,24,27–29^ and the medial temporal lobe^22,23,30^. However, it is unclear whether these activity patterns exhibit the other properties expected of symbols. First, with a few exceptions^29^, previous studies rarely assessed categorical structure by testing systematically whether activity varies discretely with continuous variation in task parameters. Second, evidence for recombination in activity is also rare, with a notable exception being hippocampal neurons that encode novel spatial paths that seem to reuse parts of experienced paths^31,32^. However, whether these continuous paths reflect a recombination of discrete components is unclear. Third, the tasks in these hippocampal and other studies of invariant representations generally lack tests of compositional generalization. Consequently, it is unclear whether and how identified activity patterns support compositional processes. Thus, we still lack evidence for a neural representation of symbols. That is, activity that jointly exhibits invariance, categorical structure and recombination in the behavioural setting of compositional generalization.

**Fig. 1:**
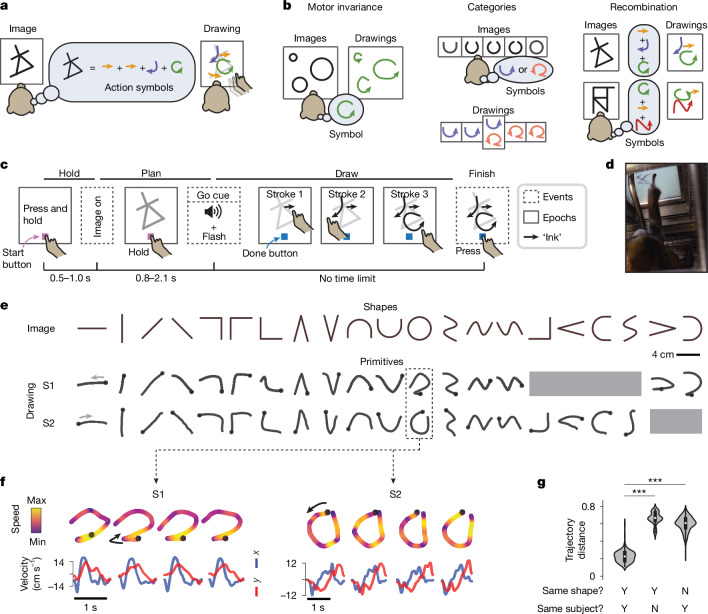
Learned stroke primitives in a drawing-like task paradigm. **a**, Schematic of the experiment. The subjects (S1 and S2) draw images by tracing on a touchscreen, with no cues for what strokes to make (for example, stroke type and order). We tested whether strokes are internally represented as learned action symbols. **b**, Schematic of three essential properties of symbolic representation: invariance, categorical structure and recombination. Predictions according to the action symbol hypothesis are shown. Alternative predictions are shown in Fig. 2a,f,k. **c**, Schematic of the trial structure, showing three discrete events (dashed boxes) and sustained epochs (solid boxes). Hold: press and hold a start button, which enforces a consistent posture. Plan: the subjects see the image but must maintain their hold on the start button. Draw: the subjects produce strokes. Black arrows represent ‘ink’ (visible to the subject) marking touched locations. The subjects can report completion at any time by pressing the done button. They receive a juice reward based mainly on drawing accuracy (Methods). Note that here, buttons always refers to touchscreen buttons. **d**, Photograph during in-cage training. The metal tube is the reward spout. **e**, Learned stroke primitives for each shape. Stroke onsets are marked with a circle. Grey shading marks images for which the subjects did not readily learn primitives, either because they used two strokes or used one stroke in a variable manner (Methods). Drawings averaged over 15 trials. **f**, Four example trials per subject for the ‘circle’ primitive, depicted spatially (top) and temporally (bottom). **g**, Summary of stroke trajectory distances for trial pairs defined along two dimensions: same shape (yes (Y) or no (N)) and same subject (yes or no). Shown is a violin plot of kernel density estimates, with the inner box plots showing medians and quartiles. *n* = 300 (same shape, same subject: YY and YN; 15 shapes × 10 trials × 2 subjects), *n* = 4,200 (NY; 15 shapes × 10 trials  × 14 other shapes × 2 subjects). ***P = 6.10 × 10^−5^, two-sided Wilcoxon signed-rank test (*W* = 0, *n* = 15 shapes, averaged over trials and subjects). Source data

To search for such a representation, we developed a task that involved symbol-based compositional generalization implemented in macaque monkeys (Fig. 1a,b). This task includes the generation of novel, goal-directed action sequences, an ability that is thought to often involve the recombination of discrete units of motor behaviour, or action symbols, into sequences^9–12,15,33^. For example, imitating a dance may depend on symbolic representations of dance poses^11^. Action symbols are also essential to various computational models of action sequencing, including in handwriting^9^, drawing^10^, object manipulation^34^ and tool use^35^, and may be related to movement segments identified in naturalistic animal behaviours^15,36,37^. Thus, a task that requires compositional generalization in action sequencing may be ideal for studying the neural basis of action symbols. Here we establish such a task and then, through behavioural and neural analyses, identify a neural representation of action symbols in the ventral premotor cortex (PMv).

## Learned stroke primitives in a drawing-like task

**Extended Data Fig. 1:**
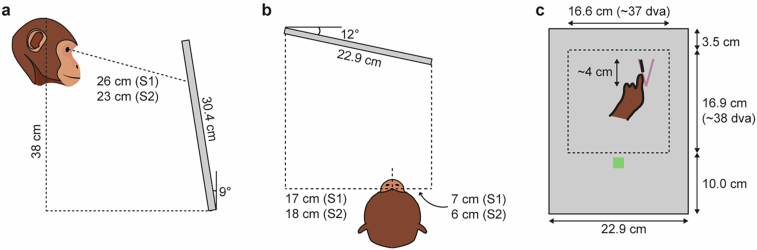
Behavioral task setup. (a). Schematic of subject relative to touchscreen, profile view. Subjects 1 (S1) and 2 (S2) were positioned at different distances to accommodate their individual anatomies and postures. The screen was slanted slightly to optimize the ability to see and reach to the same part of the screen (the workspace at the top of the screen; see panel c). (b). Schematic of subject relative to screen, top view. Subjects were positioned to the right to accommodate reaching with the hand used for drawing (left). (c). Schematic of screen during trial, with component locations and sizes to scale. The finger is tracing over the figure (purple-gray “V”), leaving a black trail of “ink” behind. The average size of shapes was 4.0 cm (maximum of width and height). The thickness and color of the figure and ink are to scale. The subject can press the “done” button (green square) at any time to report completion. Dashed line (not visible to the subject) indicates the workspace where images and drawings were contained. dva, degrees visual angle.

Modelled after studies of drawing^10^ and handwriting^9^, we trained two macaque monkeys (subject 1 and subject 2) to draw varied geometric figures by tracing on a touchscreen (Fig. 1a,c,d and Supplementary Videos 1–10; the setup is illustrated in Extended Data Fig. 1). The subjects were rewarded for accurately copying the target image (assessed primarily using the Hausdorff distance; Methods).

**Extended Data Fig. 2:**
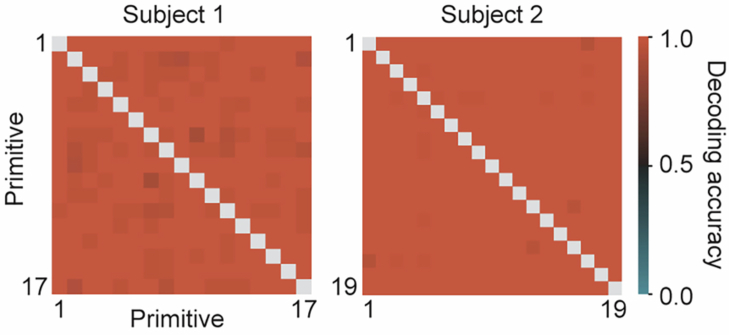
Primitives are distinct from each other in their single-trial kinematic trajectories. Pairwise decoding accuracy for all pairs of primitives, using single-trial stroke kinematics. Strokes were represented as 8D vectors derived from their x- and y-position time series (see Methods). Cross-validated decoding was performed separately for each pair of primitives, and the results for all pairs are combined in these summary matrices. Chance decoding is 0.5, because each analysis had two primitive classes. Decoding accuracy was high for all primitive pairs (minimum accuracy was 0.90 for S1 and 0.94 for S2).

The subjects practised a diverse set of simple shapes, each using one stroke (Fig. 1e). Although we did not force them to use particular spatiotemporal trajectories (that is, strokes), through practice, each subject learned a set of idiosyncratic strokes, one for each shape, which we call the subject’s primitives (Fig. 1e). To analyse the primitives, we devised a pairwise trajectory distance metric based on the Euclidean distance between the normalized velocity time series of the two strokes (Methods). For each subject, each shape was consistently drawn with the same primitive (Fig. 1f,g), and different shapes were drawn with distinct primitives (Fig. 1e–g). A further decoding-based analysis confirmed that the primitives were easily distinguishable on the basis of single-trial trajectories (Extended Data Fig. 2). The primitives were also unique to each subject, because subject 1 and subject 2 used different primitives even for identical shapes (Fig. 1e–g).

To test whether these idiosyncratic primitives reflect action symbols, we analysed how the subjects generalize to draw new figures that assess three symbol properties: motor invariance, categorical structure and recombination (Fig. 1b).

## Primitives exhibit motor invariance over location and size

**Fig. 2:**
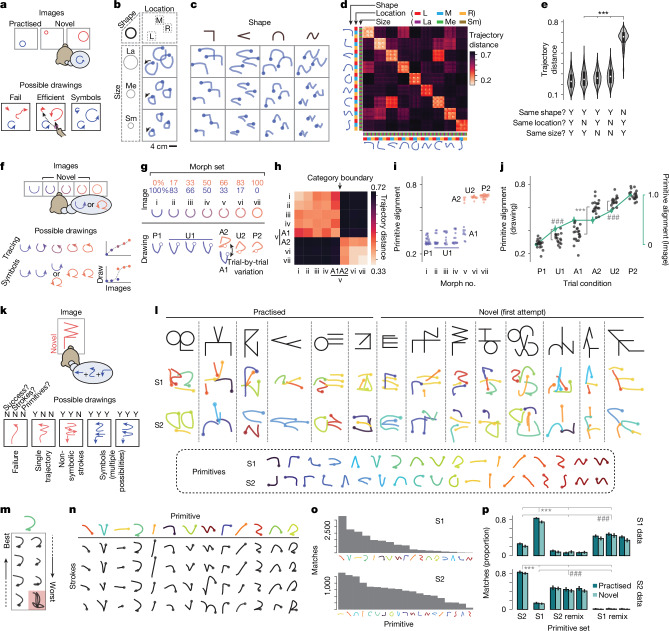
Behavioural evidence for action symbols based on motor invariance, categorical structure and recombination into sequences. Data are shown for motor invariance (**a**–**e**), categorical structure (**f**–**j**) and recombination (**k**–**p**). **a**, Schematic of the experiment testing motor invariance, with three possible drawing outcomes. Shapes vary in size (some novel) and location. **b**, Example drawings for the circle shape (nine total trials, three in each subpanel), varying in location (left (L), middle (M) or right (R)) and size (small (Sm), medium (Me) or large (La)) from S2. **c**, More example drawings. **d**, Mean trial-level pairwise trajectory distance between each shape–location–size condition (*n* = 9 trials per condition). **e**, Summary of pairwise trajectory distances (trial versus shape–size–location condition). Shown are kernel density estimates, with medians and quartiles. *n* = 648 trials (same shape, size and location: YYY), 1,296 (YNY), 1,296 (YYN), 2,592 (YNN) and 5,184 (NYY). ***P < 0.0001 (NYY versus others). Statistical details are in the Methods. **f**, Schematic of the experiment testing categorical structure, using novel images that morph parametrically between two well-practised shapes. Two hypotheses for possible drawings are shown. **g**, Example drawings for a morph set varying between image i (100% U, 0% circle) and image vii (0% U, 100% circle). Trial conditions were labelled as practised (P), unambiguous (U) or ambiguous (A). **h**, Pairwise trajectory distances for the morph set in **g**. Category boundary is defined as the morph that led to discrete drawing variation (*n* = 5−30 trials). **i**, Single-trial primitive alignment versus morph number for the set in **g** (*n* = 5−30 trials). Primitive alignment is defined as *d*_1_/(*d*_1_ + *d*_2_), where *d*_1_ and *d*_2_ are the average trajectory distances to P1 and P2, respectively. Note that applying the primitive alignment score to images instead produced a linear relationship with morph number (Extended Data Fig. 3c). **j**, Summary of primitive alignment across experiments, using both drawing trajectories (black) and image data (green, mean and 95% confidence interval; rescaled so that end points match drawing scores); *n *= 20 morph sets (S1: 13, S2: 7). ^###^*P* = 1.91 × 10^−6^ (sigmoidal nonlinearity); ***P = 1.91 × 10^−6^ (trial-by-trial switching). Statistical details in the Methods. **k**, Schematic of the experiment testing recombination in the character task, with four possible drawing outcomes. There are multiple ways to reuse primitives (symbols). **l**, Example drawings, with strokes colour-coded by the best match from each subject’s own primitives, which are plotted underneath. **m**, Example strokes assigned to the reversed C primitive, ranging from the best to the worst match. Red background, failure to have high-quality match to any primitive. Data are for S1. **n**, Example strokes and their matching primitives, ordered from high (top) to median (bottom) quality match. Data are for S1. **o**, Match frequency distribution by primitive. **p**, Summary of primitive reuse for characters performed by both subjects, showing the proportion (mean and 95% confidence interval) of strokes (data) matching a primitive from each of the subjects’ primitive sets (S1 and S2) and three simulated sets that remix subparts of primitives; *n* (characters) = 133 (S1, practised), 90 (S1, novel), 70 (S2, practised), 135 (S2, novel). ***P < 0.0001 (own set versus others). ^###^*P* < 0.0001 (own remixed set versus others). Statistical details in the Methods. Source data

Symbols are expected to exhibit motor invariance, with the idiosyncratic trajectory of each primitive generalizing across motor parameters (for example, muscle activity patterns), as in handwriting and other skills^38^. To test this possibility, we presented each shape at varying sizes and locations that are known to drive variation in motor cortical and muscle activity (Methods). If there is invariance, trajectories across location and size will be similar (Fig. 2a, symbols). Alternatively, if responses were memorized for each stimulus, then the subjects would have difficulty generalizing, as in overtrained behaviours^39^ (Fig. 2a, fail). A third strategy would involve prioritizing efficiency by minimizing movement from the starting location, which would lead to different trajectories (Fig. 2a, efficient). We found that stroke trajectories were similar across locations and sizes (Fig. 2b–e), thereby indicating motor invariance.

## Primitives exhibit categorical structure

If primitives reflect categorically structured symbols (discrete action types, such as strokes in Chinese characters or phonemes in speech^12^), we anticipated that the subjects would be biased towards using their primitives when challenged with new figures that interpolate (or morph) between learned shapes (Fig. 2f, symbols). By contrast, if the subjects trace figures closely without interpreting them as symbols, we anticipated that the drawings would closely match the interpolated figures (Fig. 2f, tracing). We presented images randomly sampled from morph sets, whereby each set had two practised shapes and four to five morphs between them. We tested whether the resulting drawings reflect categorical structure by assessing two behavioural hallmarks (Fig. 2f, symbols): a steep sigmoidal relationship between image and drawing variation^29^ and trial-by-trial variation between two distinct primitives given the same image on the boundary between categories.

**Extended Data Fig. 3:**
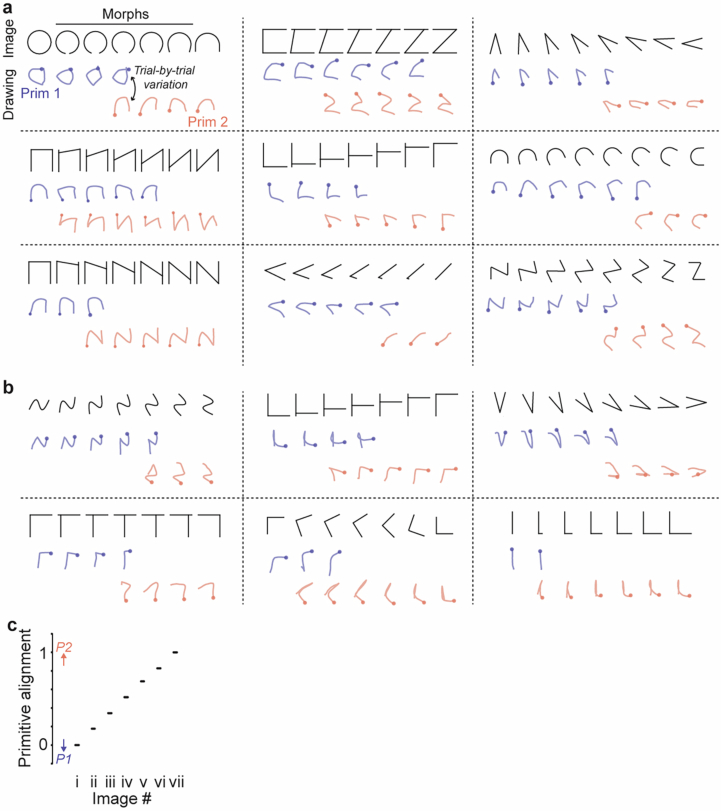
Categorical structure in behavior: more examples and quantification of primitive alignment for image data. (a). Example single-trial drawings across nine different morph sets, for subject 2. Drawings are colored by whether they reflect use of primitive 1 (blue) or primitive 2 (orange). Images morph between two well-practiced shapes (the two endpoint shapes). The examples here are depicted similarly to the example in Fig. 2g. (b). Same as panel a, but for subject 1. (c). Primitive alignment vs. trial condition for the example experiment in Fig. 2g, performed on image data (using image distance; Methods), and not on behavioral data as in Fig. 2i (which used trajectory distance).

The behaviour of the subjects exhibited both hallmarks of categorical structure. In the example in Fig. 2g (additional examples are provided in Extended Data Fig. 3), the images morphed between two practised shapes, a U shape (morph i) and a circle (morph vii). We defined the category boundary as the morph that induced trial-by-trial variation in behaviour (morph v). Drawings for morphs to the left of that boundary (unambiguous trial condition 1, U1) were similar to primitive 1 (practised trial condition 1, P1), and drawings to the right (U2) were similar to primitive 2 (P2) (Fig. 2g). This pattern was evident as two blocks in a matrix of pairwise trajectory distances (Fig. 2h). We devised a primitive alignment score, which quantifies the relative similarity of a drawing to P1 (alignment = 0) and P2 (alignment = 1) (details in Fig. 2i and Methods). Primitive alignment exhibited a sigmoidal relationship with morph number (Fig. 2i) and discrete trial-by-trial variation for morphs at the category boundary (morph v, Fig. 2i). These effects—sigmoidal nonlinearity and trial-by-trial variation—were consistent across the subjects and morph sets (Fig. 2j). Thus, behaviour reflects categorical stroke types.

## Primitives are recombined into sequences

**Extended Data Fig. 4:**
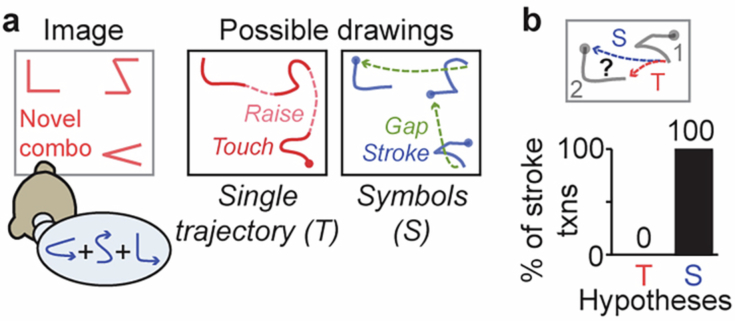
Recombination of stroke primitives into sequences, in the multishape task. (a). Schematic of experiment testing for recombination of primitives into sequences, using the multishape task. Given images composed of multiple disconnected shapes, two possible drawing responses are shown, consistent with either the “Single trajectory” (T) or “Symbols” (S) hypotheses (main text). (b). Percent of stroke-to-stroke transitions in which the second stroke is drawn in a manner consistent with the Single trajectory (T) or Symbols (S) strategies, restricted to transitions where the behavioral predictions of these two strategies differed [bottom bar plot, n = 391 transitions (S1), 529 (S2), combined]. Top, schematic of an example transition between two strokes labeled 1 and 2. Stroke 2 can be drawn either starting from the top-left, consistent with primitive reuse (“Symbols”), or from the bottom right, which would reflect the subject taking the shorter of the two gap distances (“Single trajectory”).

If the subjects represent primitives as symbols, they should recombine primitives to construct multistroke drawings. We tested this possibility in two tasks that challenged the subjects with complex figures, including novel ones. The first multishape task used figures that combined two to four disconnected shapes that could be drawn in any order. For this task, we predicted two approaches that could be used to perform the drawings. One strategy would be to use a single trajectory that efficiently traces over all shapes with appropriately timed screen touches and raises to make the strokes (single trajectory, Extended Data Fig. 4a). Behaviour would be biased to minimize the movement distance in gaps between shapes. A second strategy would be to recombine learned primitives at the cost of potentially longer gap distances (symbols, Extended Data Fig. 4a). The subjects recombined primitives at the cost of longer movements in gaps, a pattern consistent with a symbolic representation (Extended Data Fig. 4b).

The second task used complex figures called characters. Although characters were designed by connecting multiple simple shapes, the subjects were not forced to use primitives that matched these shapes. Moreover, characters had ambiguity in being consistent with multiple possible interpretations of the composition of their shape components. We considered four possible outcomes (Fig. 2k): failure to draw these novel figures (failure); success using a single trajectory (single trajectory); success using multiple strokes not in the set of learned primitives (nonsymbolic strokes); or success while reusing primitives (symbols).

The drawings were consistent with symbols. The subjects successfully drew novel characters and did so using multiple strokes (Fig. 2l and Supplementary Videos 1–10), thus contradicting the failure and single-trajectory predictions. Given the same images, the two subjects produced different drawings (Fig. 2l), which raised the possibility that they reused their own primitives. To directly test this idea, we quantified how often the strokes closely matched each subject’s own primitives using a trajectory-distance-based classification procedure that labelled each stroke as either similar to a specific primitive (match) or as failing to match any primitive (nonmatch; Methods). The resulting matches are shown as examples in Fig. 2m,n and as a frequency distribution in Fig. 2o.

**Extended Data Fig. 5:**
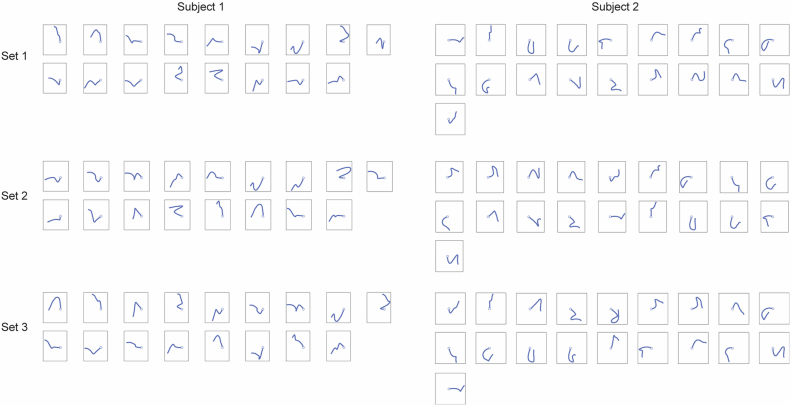
Simulated primitives created by “remixing” each subject’s actual primitives. The three sets of remixed primitives used in Fig. 2p (“remixed” sets) for each subject are shown. Each remixed primitive was constructed by connecting the first half of one primitive to the second half of another primitive (both from the same subject). Each set was optimized so that remixed primitives within a set were sufficiently different from each other and were also sufficiently different from all actual primitives. See Methods for details.

Most strokes were classified as matches to each subject’s own primitives (>82% for both subjects, Fig. 2p), even for novel characters (>74%, Fig. 2p). Two control analyses tested how specific these matches were to each subject’s own primitives. First, we asked how often the strokes matched the other subject’s primitives and found that this was rare (<21% for novel characters, Fig. 2p). Second, we asked how often strokes matched simulated ‘remixed’ primitives designed to resemble each subject’s own primitives in their subparts (first and second halves) but not in their entire trajectories (Extended Data Fig. 5). Strokes infrequently matched these remixed primitives (<43% for novel characters, Fig. 2p). Combined, these analyses show that character strokes resemble each subject’s own primitives (first analysis) in their idiosyncratic trajectories (second analysis). Thus, the subjects drew novel figures by recombining their own primitives.

## Multiarea recordings across the frontal cortex

**Fig. 3:**
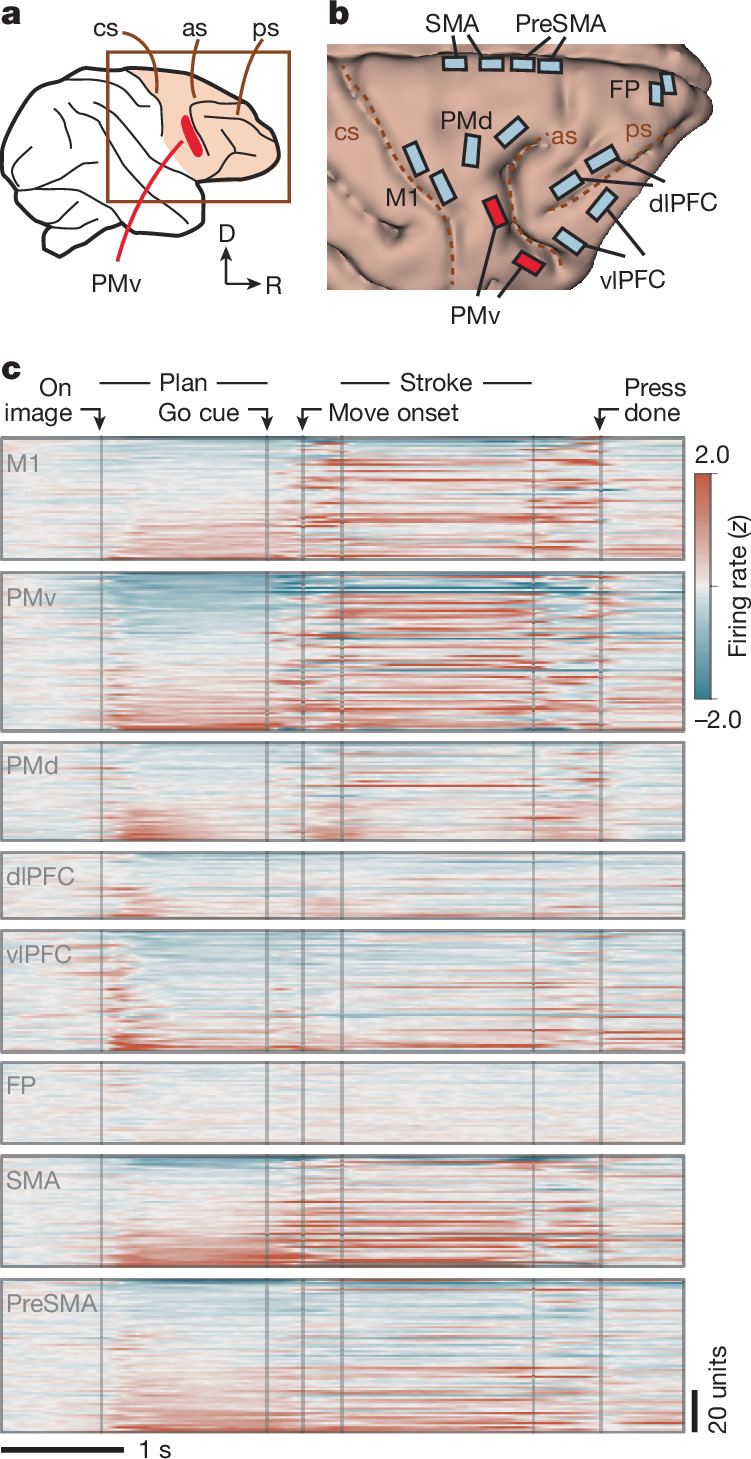
Multiarea neuronal recordings across the frontal cortex. **a**, Recordings were targeted to the right frontal cortex, contralateral to the drawing hand. As, arcuate sulcus, cs, central sulcus; D, dorsal; ps, principal sulcus; R, rostral. **b**, Floating microelectrode arrays (32-channel, Microprobes) to scale on a three-dimensional rendering of the brain (for S1). SMA and preSMA electrodes were angled to target the medial wall. For S2 and details on medial wall targeting, see Extended Data Fig. 6. We targeted regions associated with the following functions: planning and control of movements in M1, PMd and PMv^25^; abstraction and reasoning in dlPFC and vlPFC^23,27–29^ and FP; and sequencing in SMA and preSMA^42^ (details in the Methods). We recorded 48.4 ± 19.9 units per area (mean and s.d.) for S1 and 48.0 ± 16.0 for S2. **c**, Average activity across trials, grouped by brain area and split by unit (inner rows; both single-unit and multiunit). Trials were aligned by linear time-warping (Methods). Activity was *z*-scored relative to the baseline (preceding image onset) and units were sorted by activity during planning, in a cross-validated manner (Methods). Data are for S2.

**Extended Data Fig. 6:**
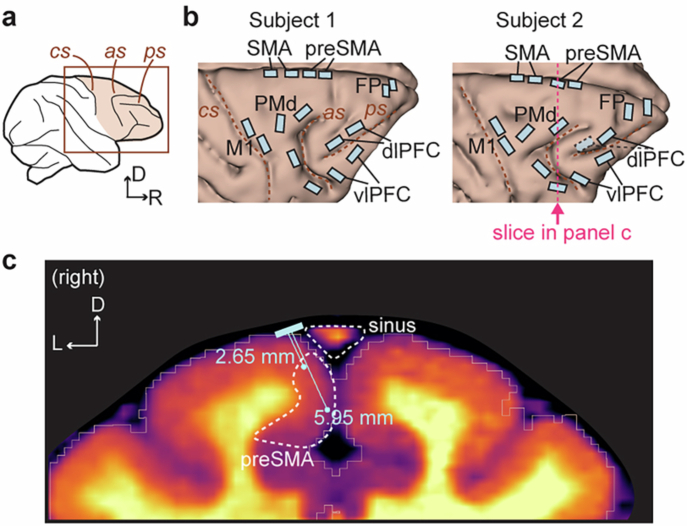
Multi-area neuronal recordings across frontal cortex and angled implantation of medial wall arrays. (a). Recordings were targeted to right frontal cortex, contralateral to the drawing hand. cs, central sulcus; as, arcuate sulcus; ps, principal sulcus. R, rostral. D, dorsal. (b). Arrays to scale on 3D rendering of brain. The caudal dlPFC array for subject 2 malfunctioned. SMA and preSMA electrodes were angled to target the medial wall (see panel c). Panels a and b (subject 1) are reproduced from Fig. 3. (c). Coronal MRI section showing SMA and preSMA arrays angled to avoid the superior sagittal sinus and target the medial wall, depicted for a preSMA array. Arrays were implanted 2 mm lateral to the midline, and angled medially. The array is depicted as a blue rectangle and two lines represent the shortest (2.65 mm) and longest (5.95 mm) electrodes. This location and angle is our best estimate based on the surgical plan, stereotactic coordinates, and intra-operative photographs. The other two SMA and one preSMA arrays were angled similarly. D, dorsal. L, lateral.

These behavioural findings indicate that stroke primitives have an underlying symbolic representation. We next searched for neural activity that corresponded to such a representation. We recorded neurons simultaneously across multiple areas of the frontal cortex using chronically implanted multielectrode arrays (16 32-channel arrays; Fig. 3a,b and Extended Data Fig. 6). We targeted eight regions associated with motor, planning and other cognitive functions (details in Fig. 3b and Methods). We found clear task-related activity in all areas except the frontopolar cortex (FP) (Fig. 3c). Furthermore, these activity patterns differed across areas with respect to image onset, planning and movement (Fig. 3c). For example, many units (each a single neuron or a few combined neurons; Methods) in the ventrolateral prefrontal cortex (vlPFC), dorsolateral prefrontal cortex (dlPFC), dorsal premotor cortex (PMd) and PMv exhibited rapid responses locked to image onset and varying activity during the planning epoch. By contrast, the primary motor area (M1) had strong movement-related activity during the stroke epoch. We tested whether activity encodes primitives in a manner exhibiting motor invariance, categorical structure and recombination. We found that each of these properties was strongest in a single area: PMv.

## Motor-invariant encoding of primitives in PMv

**Fig. 4:**
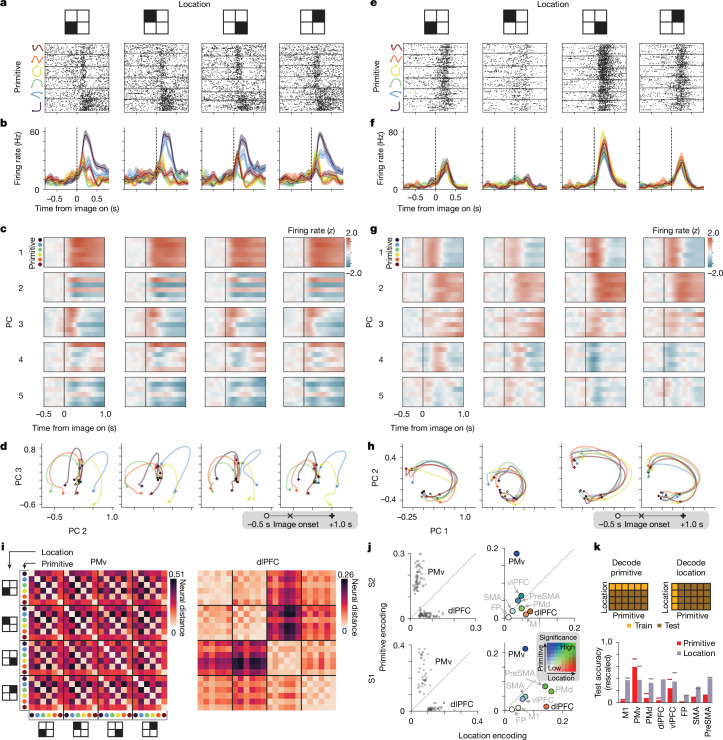
Location-invariant encoding of primitives in PMv. Data shown are for PMv (**a**–**d**,**i**), dlPFC (**e**–**h**,**i)** and all areas (**j**,**k**). Size invariance is shown in Extended Data Fig. 10. **a**, Raster plot for an example PMv unit. Trials (inner rows) are grouped by primitive and location. Holding finger ‘fixation’ is enforced throughout the duration (go cue at 1.1−1.5 s). **b**, Firing rates (mean and s.e.m.) for the example unit (*n* = 13−20 trials). **c**, Mean PMv population activity in primitive-encoding principal components (PCs), *z*-scored relative to before the image onset. **d**, Mean PMv population trajectory, with the timeline in the grey box (× indicates image onset). **e**, Analogous to **a**, but for a dlPFC unit. **f**, Analogous to **b**, but for the dlPFC unit. **g**, Analogous to **c**, but for dlPFC. **h**, Analogous to **d**, but for dlPFC, plotting PC 1 and PC 2 to emphasize the main effect of location. **i**, Pairwise neural distance between primitive–location conditions, for the session in **a**–**h**, averaged over time (0.05–0.6 s); *n* = 13−20 trials. Neural distance is defined as the mean pairwise Euclidean distance between all across-condition trials, subtracting within-condition Euclidean distances, which ranges between 0 (no difference) and 1 (ceiling, 98th percentile of all pairwise Euclidean distances). **j**, Primitive and location encoding. Left, individual primitive–location conditions; *n* = 38 (S1, aggregating 2 sessions) and 60 (S2, 3 sessions). Right, mean values. Colour denotes significance: the number of other areas ‘beaten’ in pairwise tests of primitive and location encoding, ranging from low (0) to high (7); legend in inset heatmap. For statistics, data points were unique pairs of primitive–location conditions pooled across sessions (2 for S1; 3 for S2); primitive: *n* = 93 (S1), 288 (S2); location: *n* = 114 (S1), 132 (S2). For exact statistics, see Extended Data Fig. 8a,b. **k**, Across-condition decoder generalization for primitive (red) and location (grey), with schematic of train–test split (top). Accuracy is rescaled between 0 (chance) and 1 (100%). Horizontal lines, within-condition decoding. Source data

**Extended Data Fig. 7:**
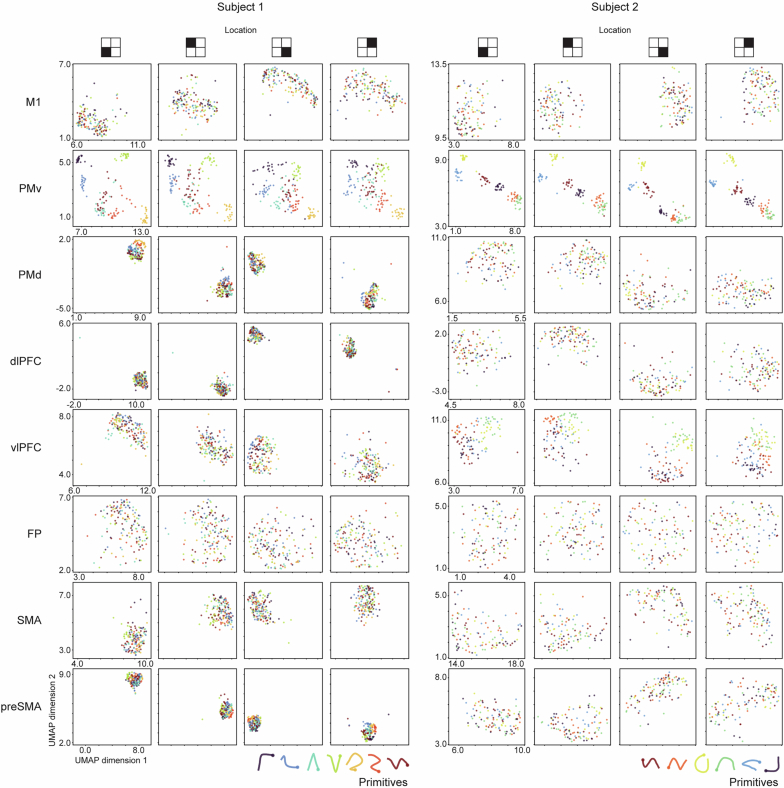
Separation of single-trial activity by primitive, invariant to location. Two-dimensional embedding of single-trial population activity using Uniform Manifold Approximation and Projection (UMAP). Each point represents a single trial, colored by primitive and split by brain region (rows) and screen location (columns). UMAP was performed on a vector representation of single-trial activity, constructed by concatenating all units’ time-varying activities into a single vector (Methods). Activity was taken from the planning epoch (0.05 to 0.6 sec after image onset), from a single session of the single shape task, in which primitives varied in location. It is visually apparent that PMv activity clusters by primitive and that this clustering is largely unaffected by location. In contrast, activity in other regions is either unstructured (FP), varies by location with a relatively weaker effect of primitive (M1, PMd, dlPFC, SMA, preSMA), or varies somewhat by primitive but in a manner that depends on location (vlPFC).

In the single-shape task, we analysed activity during the planning epoch (between image onset and the go cue) as the subjects drew primitives that varied in location (Fig. 2a–e). PMv activity varied depending on the planned primitive, with relatively little influence of location. For example, the unit in Fig. 4a,b fired strongest for the rotated L primitive (black) regardless of location. Population activity, visualized using a linear projection to two dimensions (a primitive-encoding subspace; Methods), showed strong variation with the primitive but not with the location (Fig. 4c,d). For example, in each location, activity for each primitive separated after image onset and did so in a similar manner across locations (Fig. 4d). By contrast, concurrent activity in dlPFC (an area implicated in executive function) reflected location, with minimal effect of the primitive (Fig. 4e–h). Location-invariant encoding was apparent in PMv (and not other areas) even for individual trials (Extended Data Fig. 7).

**Extended Data Fig. 8:**
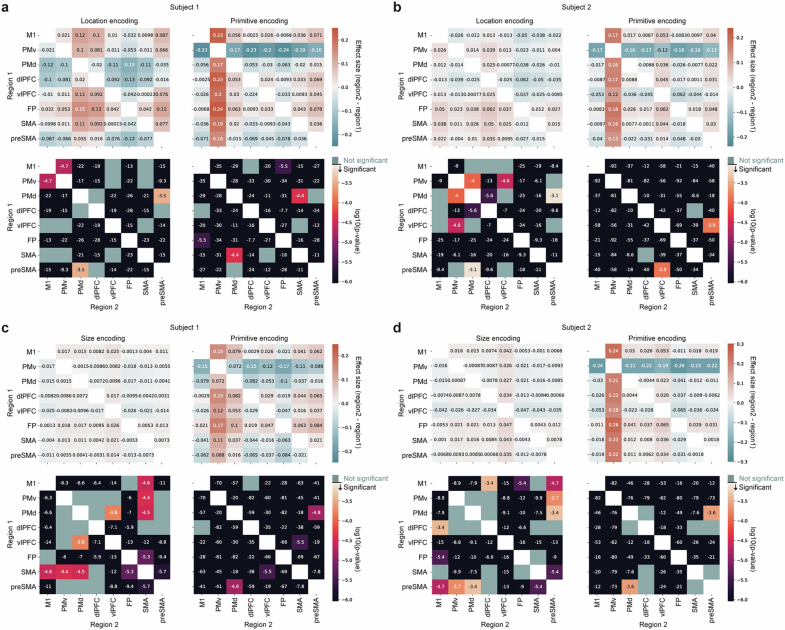
Detailed statistics for the analysis of location invariance and size invariance. Statistics are shown for analysis of location invariance in **a**,**b** (corresponding to results in Fig. 4j) and for analysis of size invariance in **c**,**d** (corresponding to results in Extended Data Fig. 10b). (a). Effect size (top) and p-value (bottom) for all pairwise comparisons between brain regions, for location encoding (left) and primitive encoding (right) in experiments varying primitive and location, for subject 1. “Significant” for the p-value heatmap means that p < 0.05 after Bonferroni correction for 56 comparisons (28 brain region pairs and 2 variables, location and primitive). (b). Same as panel a, but for subject 2. (c). Same as panel a, but for size, instead of location, encoding. (d). Same as panel c, but for subject 2.

To quantify the extent to which primitives and locations were encoded in activity, we devised neural distance: a Euclidean-distance-based metric of population activity dissimilarity between any two primitive–location conditions (details in Fig. 4i and Methods). In PMv, neural distances between primitives computed in manner that controls for location (primitive encoding) were higher than distances between locations controlling for primitives (location encoding). This pattern was evident as off-diagonal streaks in a matrix of pairwise distances (Fig. 4i and quantified in Fig. 4j, left column). In dlPFC, the opposite was true, whereby there was low primitive encoding and high location encoding, which was evident as four diagonal blocks in the distance matrix (Fig. 4i and quantified in Fig. 4j, left column). Summarizing across areas (Fig. 4j, right column, with statistics in Extended Data Fig. 8), PMv was the only region with strong primitive encoding and weak location encoding.

To directly test that PMv neural population code for primitives is similar across locations, we used a decoding approach. We found that a linear decoder that we trained to decode primitives at one location using PMv activity generalized well to decode primitives in held-out locations^23^ (Fig. 4k).

**Extended Data Fig. 9:**
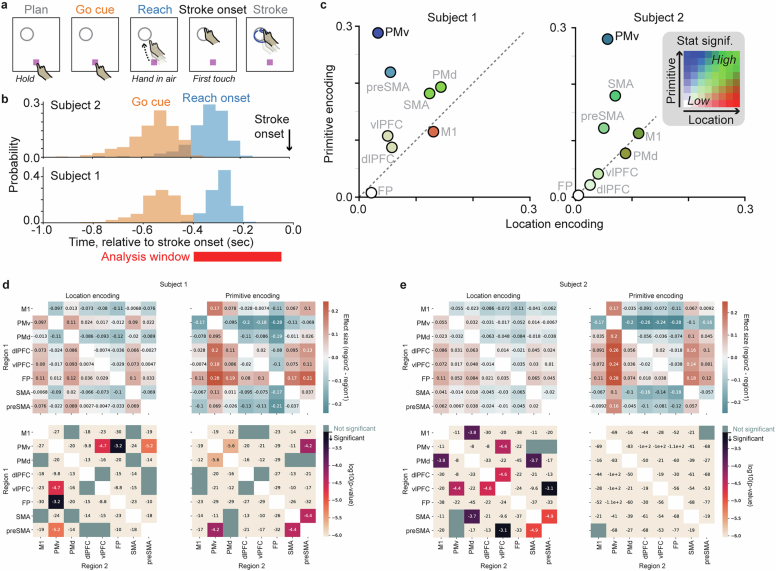
Location-invariant encoding of primitives in PMv during the initial reaching movement. (a). Schematic of trial events, highlighting the initial reaching movement from the location of the “hold” button to the location of stroke onset. (b). Histogram of times of the go cue (orange) and reach onset (blue) relative to the time of stroke onset. To analyze activity during the initial reach, we used the time window -0.4 s to -0.05 s relative to stroke onset. (c). Summary of primitive encoding and location encoding across areas and sessions, showing means. The color of each point denotes statistical significance (explained in Fig. 4j). Each data point was a unique pair of primitive-location conditions (trial-averaged). Primitive encoding (same location, different primitive): n = 93 (subject 1), 288 (subject 2). Location encoding (same primitive, different location): n = 114 (S1), 132 (S2). Statistical tests were performed on condition pairs pooled across sessions (2 for S1, 3 for S2). (d). Statistics for primitive and location encoding in panel c. Effect size (top) and p-value (bottom) for all pairwise comparisons between brain regions, for location encoding (left) and primitive encoding (right), for subject 1. “Significant” for the p-value heatmap means that p < 0.05 after Bonferroni correction for 56 comparisons [28 brain region pairs and 2 variables (location and primitive)]. (e). Same as panel d, but for subject 2.

**Extended Data Fig. 10:**
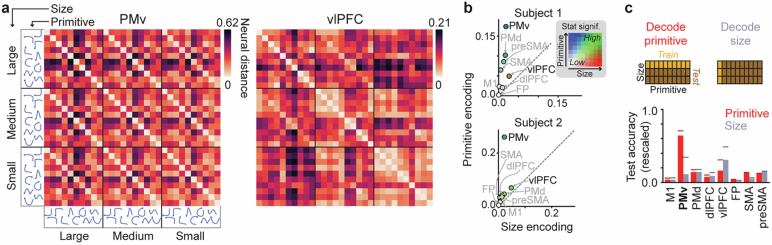
Size-invariant encoding of stroke primitives in PMv. (a). Matrix of pairwise neural distances between unique combinations of primitive and size, averaged over the planning epoch (0.05 to 0.6 s relative to image onset), for PMv and vlPFC. Shown are data from a single session for subject 2; n = 17 - 23 trials per primitive-size combination. (b). Summary of primitive encoding and size encoding across areas and sessions, with each point depicting the encoding scores for a given area. The color of each point denotes statistical significance (explained in Fig. 4j). Each data point was a unique pair of primitive-size conditions (trial-averaged). Primitive encoding (same size, different primitive): n = 313 (subject 1), 253 (subject 2). Size encoding (same primitive, different size): n = 60 (S1), 56 (S2). Statistical tests were performed on condition pairs pooled across sessions (3 for S1, 2 for S2). For exact effect sizes and p-values, see Extended Data Fig. 8c,d. (c). Across-condition generalization of linear SVM decoders for primitive (red) and size (gray). See explanation in Fig. 4k.

We performed two other tests of invariance. First, during the initial reaching movement between the go cue and the stroke onset, when motor areas strongly encode reach direction^40^, we found location-invariant encoding of primitives in PMv. By contrast, activity in M1, PMd and supplementary motor area (SMA) mixed primitive and location encoding (Extended Data Fig. 9). Second, PMv population activity during planning encoded primitives in a manner also invariant to size (Extended Data Fig. 10).

## Categorical encoding of primitives in PMv

**Fig. 5:**
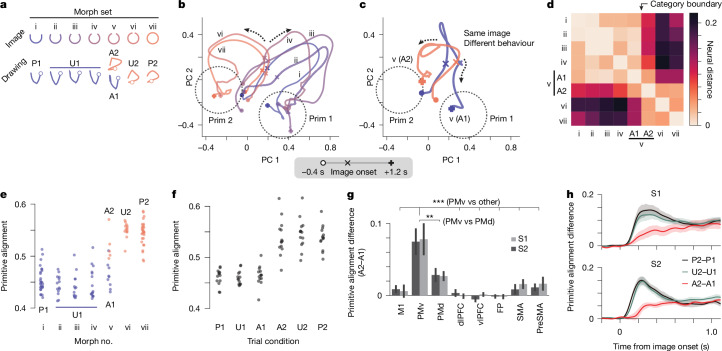
Categorical encoding of stroke primitives in PMv. **a**, Schematic of the experiment (details in Fig. 2g). **b**, PMv mean population trajectories (practiced (P1, P2) and unambiguous (U1, U2) trial condition), preceding the go cue (occurring at 1.2−1.6 s). The trajectory legend is shown below; *n* = 14−30 trials. Prim 1, primitive 1; Prim 2, primitive 2. **c**, PMv mean population trajectories (ambiguous trials (A1, A1)) for morph v, coloured by whether the subject will draw primitive 1 (A1) or primitive 2 (A2), overlaid on the same PCs as in **b**; *n* = 9 (A1) and 5 (A2) trials. **d**, Pairwise neural distances for PMv activity (*n* = 5−30 trials). **e**, PMv primitive alignment for trials in the example experiment in **a**–**d**; *n* = 5–30 trials. **f**, Summary of PMv primitive alignment versus trial condition. Each morph set contributes one point to each condition; *n* = 14 morph sets (5 from S1, 9 from S2), including only the 14 out of 20 sets that have all 6 trial conditions. **g**, Summary of the primitive alignment difference for ambiguous images (A2–A1), plotting mean ± s.e.m., **P = 0.0012. The following *P* values are versus PMv: FP, ***P = 3.39 × 10^−6^; M1, ***P = 2.69 × 10^−5^; PMd, ***P = 0.0012; SMA, ***P = 4.17 × 10^−5^; dlPFC, ***P = 4.05 × 10^−6^; preSMA, ***P = 5.76 × 10^−5^; vlPFC, ***P = 9.17 × 10^−6^; two-sided paired *t*-tests; *n* = 20 morph sets, combining S1 (7) and S2 (13). **h**, Average time course of the difference in primitive alignment for PMv activity, split by trial condition (mean ± s.e.m.). Sample size is the same as in **f**. Source data

When we combined the task that tests categorical structure (Figs. 2f–j and 5a) with recordings, we found that PMv activity during planning diverged towards separate primitive-encoding states depending on what primitive the subject will draw (Fig. 5). After image onset (the × icon in Fig. 5b), activity for trials planning primitive 1 (morphs i–iv) separated from trials planning primitive 2 (morphs vi and vii). Similarly, for the ambiguous image at the category boundary (morph v), trajectories separated towards these two states depending on whether the subject was planning to draw primitive 1 or primitive 2 (ambiguous trial condition 1 (A1) and A2, respectively, in Fig. 5c).

We quantified this separation by first computing the Euclidean distance (averaged over the planning epoch) between each pair of trials and then scoring each trial with its primitive alignment: *d*_1_/(*d*_1_ + *d*_2_), where *d*_1_ and *d*_2_ are the average Euclidean distances to P1 and P2 trials, respectively. This calculation revealed the same two hallmarks of categorical structure seen in behaviour: sigmoidal nonlinearity and trial-by-trial switching. Sigmoidal nonlinearity was evident in the matrix of pairwise neural distances as two main blocks separated by the category boundary (Fig. 5d) and in the plot of primitive alignment versus morph number (Fig. 5e,f). Trial-by-trial switching between primitive-representing states for ambiguous images was evident in the matrix of pairwise neural distances (Fig. 5d, A1 relatively close to morphs i–iv compared with A2) and in primitive alignment (morph v in Fig. 5e and A1 versus A2 in Fig. 5f). When comparing across areas, this effect was strongest in PMv (Fig. 5g).

**Extended Data Fig. 11:**
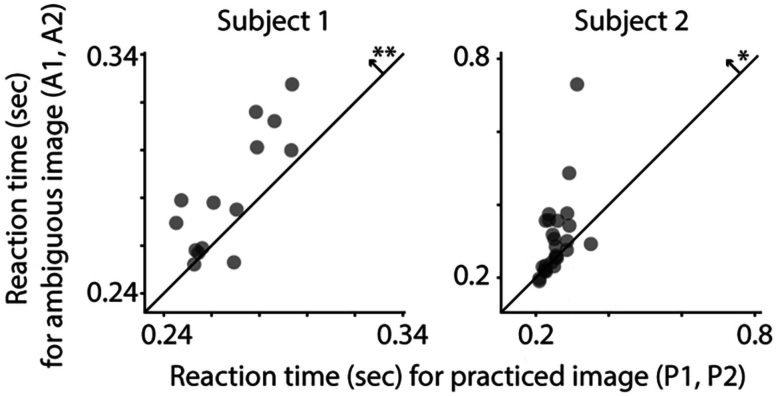
Slower reaction time for ambiguous images in tasks testing categorical structure. Average reaction time in seconds (between go cue and stroke onset), comparing ambiguous to practiced images. Each data point represents a single primitive from one morph set (each morph set contributes two primitives, one at each morph endpoint), plotting reaction time when it was drawn in response to an ambiguous image (y-axis) vs. practiced image (x-axis). **, *p* = 0.0031, two-sided Wilcoxon signed-rank test (n = 14, W = 8); *, *p* = 0.024, Wilcoxon signed-rank test (n = 26, W = 87).

Trial-by-trial switching for ambiguous images may reflect a winner-takes-all competitive process between activity states encoding different primitives^41^. Consistent with this possibility^41^, activity separation was slower for ambiguous images (A2–A1 in Fig. 5h). Mirroring the neural activity, the behavioural reaction time was also slower (Extended Data Fig. 11).

**Extended Data Fig. 12:**
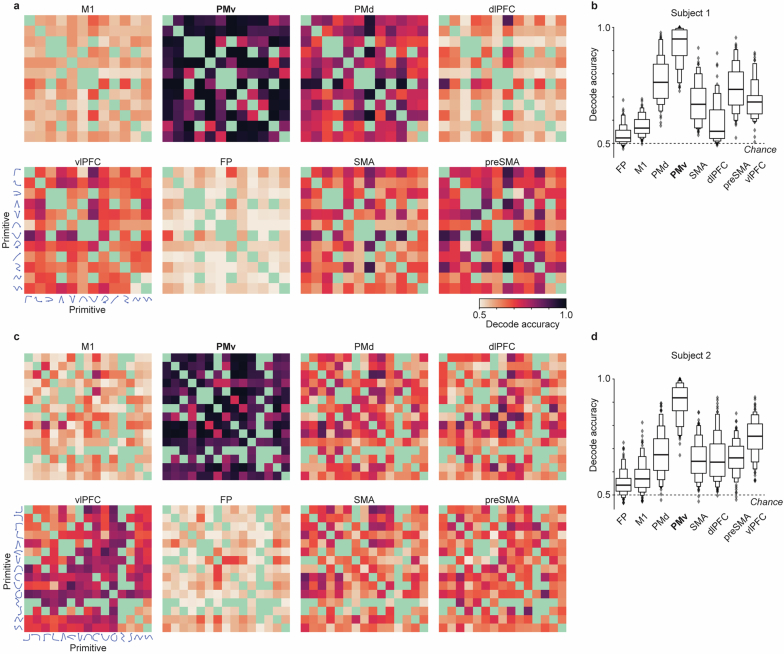
Primitives are distinct from each other in their planning-related activity in PMv. (a). Pairwise decoding between all primitives (drawn in the same session) for subject 1, using population activity from the planning epoch (0.05 to 0.6 sec after image onset) represented as a 50-dimensional vector (see Methods). Chance decoding is 0.5. The matrices aggregate across sessions (n = 5 single shape sessions, 2 varying in location, 3 varying in size). The gray-green cells are pairs which lack data because the primitives were not recorded in the same session. (b). Summary of decoding accuracy for all primitive pairs for subject 1, shown in a letter-value plot, a boxplot variant where boxes depict successively decreasing quantiles, from median, to quartile, to eighths, and so on. Each datapoint is one unique primitive pair (n = 74 pairs, across the 5 sessions from panel a). (c). Like panel a, but for subject 2 (n = 5 single shape sessions, 3 varying in location, 2 varying in size). (d). Like panel b, but for subject 2 (n = 108 unique primitive pairs, across the 5 sessions from panel c).

Categorical encoding implies that every primitive has a distinct activity pattern. Consistent with this idea, activity visualized in a two-dimensional embedding separated primitives in PMv (Extended Data Fig. 7). Moreover, when quantifying this separation, primitives were easily distinguishable on the basis of single-trial activity (Extended Data Fig. 12).

## Recombination of primitives is reflected in PMv

**Fig. 6:**
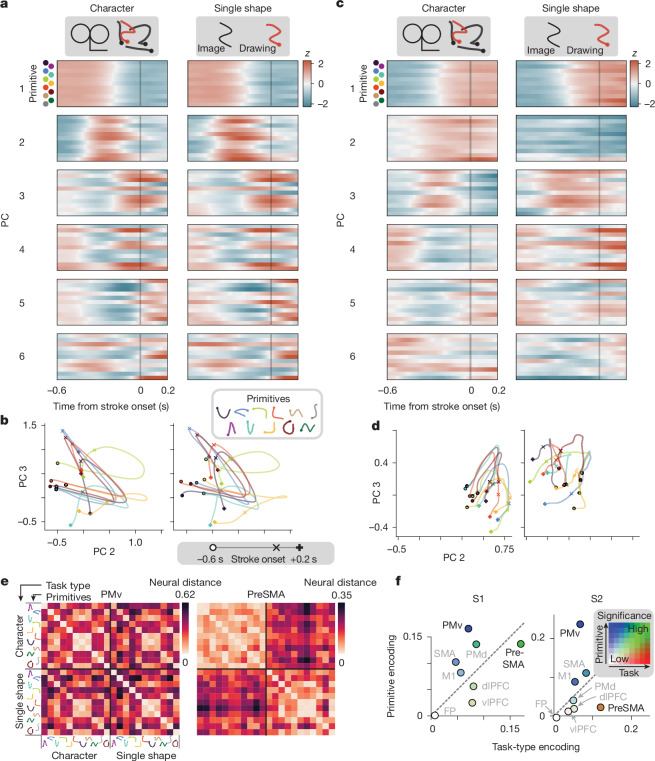
Recombination of primitives into sequences is reflected in PMv. Data shown are for PMv (**a**,**b**,**e**), preSMA (**c**–**e**), and all areas (**f**). **a**, PMv population activity in its first six PCs, split by primitive (inner rows) and task type (columns) and *z*-scored over the entire window; *n* = 4−16 trials (single-shape), 6−72 (character) for S2. Colours index the primitives in **b**. **b**, PMv population trajectories, aligned to stroke onset (marked ×), showing a subset of primitives (7 out of 11) to reduce clutter. The trajectory legend is in grey. **c**, Analogous to **a**, but for simultaneously recorded activity in preSMA. **d**, Analogous to **b**, but for preSMA. **e**, Pairwise neural distance between combinations of primitive and task type for the experiment in **a**–**d**, averaged over time (−0.5 to −0.05 s). *n* = 4−16 trials (single-shape), 6−72 (character). **f**, Summary of primitive and task-type encoding across areas and sessions. Primitive encoding is the neural distance between primitives controlling for task type, whereas task-type encoding is the distance between task types controlling for primitives. Points show means, with colour denoting significance (explained in Fig. 4j). Statistical tests were performed on condition pairs pooled across sessions (9 for S1, 9 for S2), using bootstrapping to balance sample sizes across sessions. Each data point was a unique pair of primitive–task conditions. Primitive encoding: *n* = 700 (S1), 548 (S2). Task-type encoding: *n* = 82 (S1), 64 (S2). For exact effect sizes and *P *values, see Extended Data Fig. 13. Source data

**Extended Data Fig. 13:**
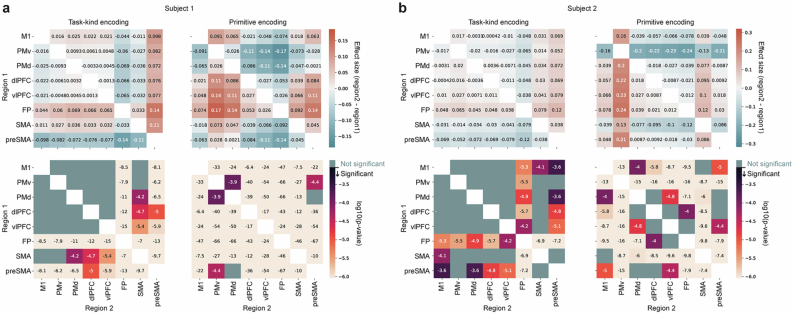
Detailed statistics for the analysis of recombination. Statistics are shown that correspond to the results in Fig. 6f. (a). Effect size (top) and p-value (bottom) for pairwise comparisons between brain regions, for task-type encoding (left) and primitive encoding (right), for subject. 1“Significant” for the p-value heatmap means that the value is less than 0.05 after Bonferroni correction for 56 comparisons [28 brain region pairs and 2 variables (task type and primitive)]. Note that because analyses involved bootstrapping to balance sample sizes across sessions (see Fig. 6f), these are statistics from a single bootstrapped sample that matches the mode. (b). Same as panel a, but for subject 2.

We next examined whether recombining primitives into multistroke characters (Fig. 2k–p) reuses primitive-encoding activity. We compared activity when a primitive was used in the single-shape task with when it was used in the context of a sequence in the character task. Because the character task involves multiple strokes, to facilitate comparison with the single-shape task, we focused on the time window immediately preceding the stroke onset (rather than the entire planning epoch). PMv population activity for each primitive was similar across task types (Fig. 6a,b, across columns), an effect apparent in the matrix of pairwise neural distances (off-diagonal bands for PMv in Fig. 6e). A summary analysis confirmed high primitive encoding and low task-type encoding (Fig. 6f). By contrast, in the presupplementary motor area (preSMA), an area involved in sequencing^42^, activity differed between task types (Fig. 6c–f). Across all areas, PMv most consistently exhibited high primitive encoding and low task-type encoding (Fig. 6f, with statistics in Extended Data Fig. 13).

**Extended Data Fig. 14:**
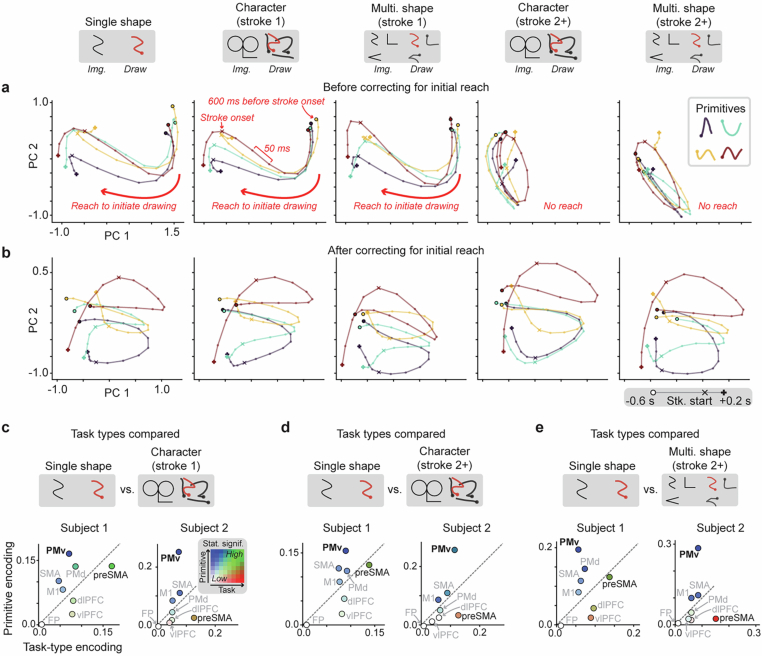
Recombination of primitives into sequences is reflected in PMv, including for the second stroke onwards. Results shown are extensions of the analysis that restricted data from character trials to only data for the first stroke, in Fig. 6. (a). PMv population trajectories for each primitive (colors) aligned to stroke onset (“x” icon). Each subpanel shows a different subset of trials (i.e., strokes) from this session, differing by task type (Single-shape, Character, or Multi-shape) and whether it includes only the first stroke (“stroke 1”) or only the second stroke onwards (“stroke 2+”), plotted in the same subspace. Note that the Single-shape task by design only has one stroke. A prominent activity pattern is the right-to-left sweep during the initial reaching movement to start the drawing, visible for the conditions involving an initial reach—Single-shape, Character (stroke 1), and Multi-shape (stroke 1)—and not for conditions lacking this reach—Character (stroke 2+) and Multi-shape (stroke 2+). (b). Analogous to panel a, but after correcting for the initial reach by subtracting, identically for all trials, the across-trial mean effect of the initial reach on neural activity, performed separately for each time bin (Methods). After applying this simple linear correction, the primitive-associated trajectories are now similar across all conditions, consistent with the reach effect being an additive component, associated with the large movement of the arm during the initial reach. (c). Summary of primitive encoding and task-type encoding across areas and sessions, comparing Single-shape to Character (stroke 1) trials, after correcting for the mean effect of initial reach on neural activity. Apart from this correction, this is the same data as in Fig. 6f. For panels c-e, the plotting conventions are identical to Fig. 6f. The color of each point denotes statistical significance (explained in Fig. 4j). (d). Same as panel c, but comparing Single-shape trials to Character (stroke 2+) trials. Each data point is a unique pair of primitive-task conditions (trial-averaged). Primitive encoding (same task type, different primitive): n = 858 (subject 1), 624 (subject 2). Task-type encoding (same primitive, different task type): n = 90 (S1), 63 (S2). (e). Same as panel c, but comparing Single-shape trials to Multi-shape (stroke 2+) trials. Primitive encoding (same task type, different primitive): n = 1458 (subject 1), 778 (subject 2). Task-type encoding (same primitive, different task type): n = 117 (S1), 75 (S2).

In the preceding analysis, to control for effects of the initial reaching movement (between release of the start button and stroke onset) on neural activity^40,43^, we included only the first stroke for the character task (the single-shape task always has one stroke). Including all character strokes (after controlling for the initial reach by applying a linear correction in a manner explained in Extended Data Fig. 14a,b and Methods) led to the same finding; that PMv exhibits high primitive encoding and low task-type encoding (Extended Data Fig. 14c–e). This result indicates reuse of primitive-encoding activity.

## PMv activity dissociates from visual and motor parameters

**Extended Data Fig. 15:**
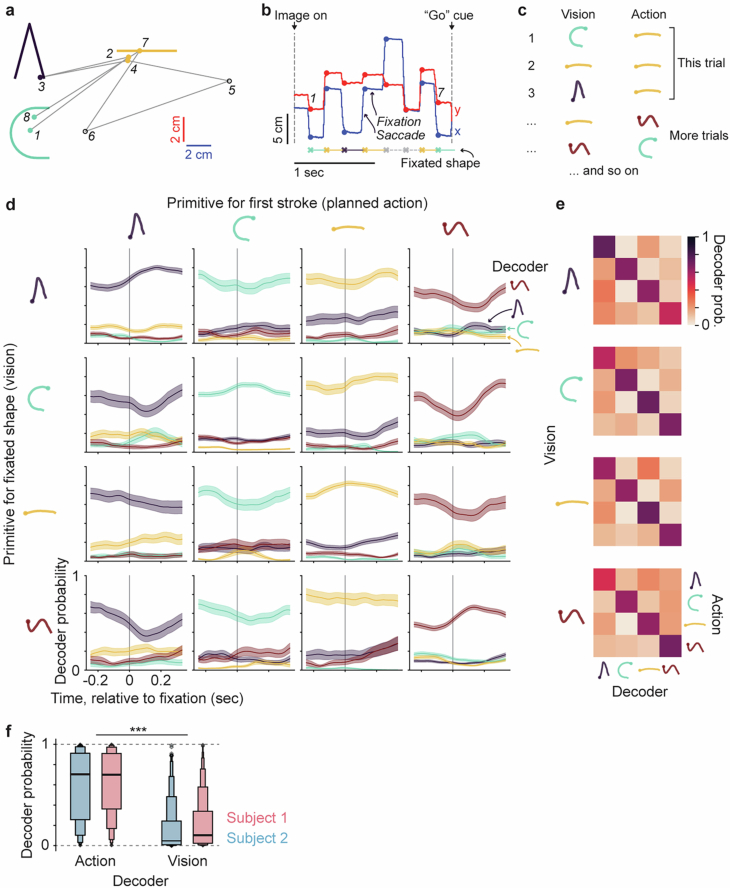
PMv activity reflects the primitive the subject is planning to draw, rather than the primitive associated with the visually fixated shape, in the Multi-shape task. (a). Gaze location during the planning epoch (between “image on” and “go cue”) for an example trial of the Multi-shape task, which uses images consisting of multiple separated shapes, with no imposed rule for drawing order (see Extended Data Fig. 4). Saccades (grey lines) and fixations (circles) overlaid on the image for this trial, numbered in order, and colored by the identity of the fixated shape. Note that shapes on the screen were not colored for the monkey. Each fixation event was assigned a “vision” label defined as its closest image shape, and a “planned action” label defined as the shape the subject chose to draw first on that trial. Fixations that were too far from any shape (here, numbers 5 and 6) were excluded from subsequent analyses (see Methods). (b). Gaze location vs. time during the planning epoch for the example trial in panel a. Gaze involved interleaved saccades (rapid change in location) and fixations (stable location) onto different shapes. The shape in view for each fixation is indicated by the colored line below (dashed gray means the fixation was too far from any shape and therefore not assigned any shape). On this trial, the image had three shapes, and the subject chose to draw the horizontal line first. (c). Analysis was performed on a dataset of planning-epoch fixation events across trials with different first-drawn primitives, thus dissociating the visually fixated shape from the primitive the subject planned to draw first. This table illustrates part of a dataset of fixation events. Note the dissociation between “vision” and “action” labels across events. (d). Average fixation-aligned decoder probability for each primitive, grouping fixation events by the planned action (columns) and the fixated shape (rows). This, and the following panels, includes fixation events from the second half of the planning epoch, when the subject is expected to have decided what to draw first. Note the high probability for the decoder for the planned action, and low probability for the decoder for the fixated shape. Further, note that the decoder probability for the planned action is not strongly modulated relative to the time of fixation onset. (e). Summary of mean decoder probability across conditions varying in vision and action. The mean is taken over the time window 0.05 to 0.3 sec after fixation onset. Note the strong diagonal band, consistent with activity encoding the planned primitive rather than the visually fixated shape. (f). Summary of decoder probability for action vs. vision, shown in a letter-value plot (explanation in Extended Data Fig. 12b). Each fixation event contributed one data point for both action and vision. This includes only fixation events with action differing from vision. ***, *p* = 7.35 × 10^−112^ (subject 1), 9.1 × 10^−72^ (subject 2), two-sided Mann–Whitney U test; n = 883 fixations (S1), 446 (S2), each from a single session.

**Extended Data Fig. 16:**
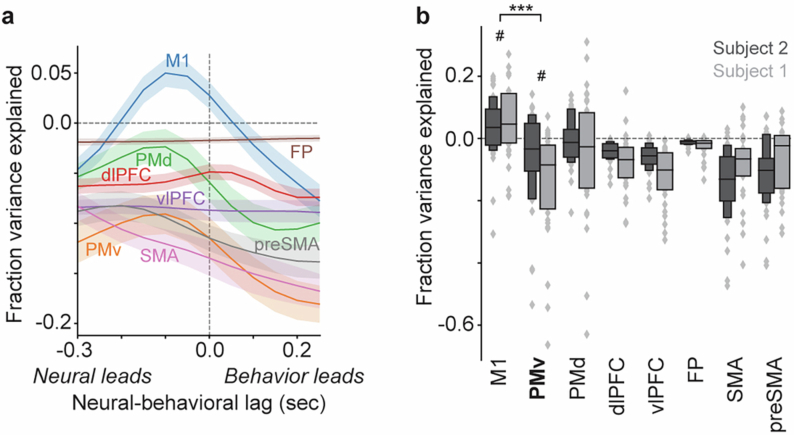
PMv activity does not significantly encode generic motor kinematics. (a). Fraction of neural activity variance (across time bins and trials) explained by stroke kinematics (x- and y-velocity) during drawing. A linear regression model, mapping from kinematics to neural activity, was trained on a subset of data consisting of every primitive except one, and then tested on the one held-out primitive, therefore testing for encoding of kinematics that generalizes across primitives. This was repeated for every primitive and averaged (S1, n = 30 primitives across five sessions; S2, n = 42 primitives across three sessions; datasets were combined in this plot). This analysis was performed separately for different time lags between the neural and behavioral data, and the resulting values were plotted as a function of time lag. Note that negative explained variance indicates failure to generalize across primitives (i.e., overfitting), with the model performing worse than an intercept-only model trained on the held-out dataset (see Methods). (b). Summary of fraction of variance explained, averaging over the time window -0.15 to -0.05 s relative to zero lag (chosen because it is centered at the time of maximum variance explained, which is consistent with the expected lag between neural activity and behavior from prior studies; Methods), summarized in a letter-value plot (explanation in Extended Data Fig. 12b). Note that M1 is the only area with positive fraction variance explained. Values for M1 (around 0.0 to 0.2) are comparable to a prior study of motor cortex in human handwriting^101^ (Methods). Statistical tests involving M1 and PMv: ***, M1 vs. PMv: S1 (W = 10, *p* = 8.01 × 10^−8^), S2 (W = 137, *p* = 3.36 × 10^−5^), S1 and S2 combined (W = 205, *p* = 4.87 × 10^−10^). #, M1 vs. zero: S1 (W = 117, *p* = 0.016), S2 (W = 230, *p* = 0.0049), combined (W = 667, *p* = 0.00028), S1 does not reach significance after Bonferroni correction. #, PMv vs. zero: S1 (W = 33, *p* = 5.97 × 10^−6^), S2 (W = 293, *p* = 0.047), combined (W = 559, *p* = 2.27 × 10^−5^), S2 does not reach significance after Bonferroni correction. Sample size for all tests: S1, n = 30 primitives across five sessions; S2, n = 42 primitives across three sessions. Bonferroni correction considered all brain regions, thus correcting for 28 tests for PMv vs. M1, and for 8 tests for PMv/M1 vs. zero.

That motor invariance (Fig. 4), categorical structure (Fig. 5) and recombination (Fig. 6) were each strongest in PMv indicates a representation of action symbols in this region. Given that PMv can encode visual stimuli^25^ and movement kinematics^25,44^, we further characterized this representation by testing how visual and motor parameters contribute to PMv activity in this task. First, we tested whether PMv is driven by the shape in view using a task that dissociates what shape the subject is looking at from what primitive it is planning to draw (multishape task). We found that PMv encodes the planned primitive rather than the shape in view (Extended Data Fig. 15). Second, we tested whether PMv encodes stroke kinematics in a generic manner (that is, generalizing across primitives). Using linear encoding models that map the stroke velocity to neural activity, we found evidence for generic velocity encoding in M1 but not in PMv (Extended Data Fig. 16). Thus, PMv activity in this task abstracts over immediate visual input and motor output.

## Discussion

### Identification of a neural substrate of action symbols

We tested for three properties of symbols: invariance, categorical structure and recombination. The monkeys traced new figures by recombining stroke primitives (Fig. 2k–p), which exhibited motor invariance (Fig. 2a–e) and categorical structure (Fig. 2f–j). In motor, premotor and prefrontal neural activity (Fig. 3), we found evidence for these three properties, which were each strongest in a single area: PMv (Figs. 4–6). These behavioural and neural findings reveal a localized representation of action symbols in PMv activity.

This result was unexpected given the evidence for encoding of abstract cognitive variables in other areas, especially PFC^6,23,24,27–29^ and the medial temporal lobe^22,23,30^. This difference in areas may reflect a fundamental difference in the role of motor behaviour. Here complex, learned movement details were relevant for success, whereas commonly studied cognitive tasks use simple movements for reporting a choice. Our results suggest that PMv has a privileged role in the abstract representation of skilled movements. In support of this possibility, human-based studies have shown that lesions to ventral premotor areas are associated with disrupted knowledge of action concepts^45^ and that activity in these areas is associated with action-related perception^46^, imitation^47^, verbalization^48^ and imagination^46^. Our findings therefore highlight a type of abstraction that is relatively understudied at the neuronal level (motor abstraction) and point to a crucial role for action symbols in PMv in such abstraction.

Our findings also provide general insights into the neural basis of symbols. Various animal behaviours seem to reflect symbols^7,13–17^. However, they may lack certain abstract properties implicated in symbol systems in human language, mathematics and abstract reasoning, including reversible reference^49^, higher-order relations^6^ and recursive syntax^5,6^. Our identification of a kind of symbolic representation in macaque motor behaviour raises the possibility that at least basic types of symbolic operations exist across species and, more speculatively, may reflect shared mechanisms for recombining discrete, invariant representations. The criteria of invariance, categorical structure and recombination may guide future studies, including reassessing findings of invariant neural activity in cognitive tasks^6,22–30^.

### Action symbols and abstraction in PMv

Our findings indicate that this action symbol representation is not directly driven by visual features or motor parameters. First, PMv encoded the planned primitive in a manner dissociated from visual features, including of the shape in view (Extended Data Fig. 15), of ambiguous shapes (A2 differs from A1 in Fig. 5f), and of complex charcaters (low task-type encoding in Fig. 6f). Second, independence from motor parameters was indicated by three properties of primitive-encoding activity in PMv: presence during planning, temporally dissociated from drawing (by >1 s; see, for example, Fig. 5h); invariance to location and size (Fig. 4 and Extended Data Figs. 9 and 10); and lack of generic kinematic encoding (Extended Data Fig. 16). These properties are in contrast to the motor-related properties of M1, including lack of primitive-encoding during planning (Fig. 4j), lack of location-invariance during initial reach (Extended Data Fig. 9) and encoding of generic kinematics (Extended Data Fig. 16). Thus, PMv encodes action symbols in an abstract manner.

Motor-invariant activity has been observed in PMv, especially during grasping and object manipulation^25,44,50^, including invariance to effector and muscle activity patterns. PMv also contains so-called ‘mirror neurons’ that fire similarly whether one observes or performs a given action^50^. These abstract properties have been proposed to reflect various possible functions, including visuomotor transformation^25,51^, action understanding^50^, imitation^50^ and, related to action symbols, encoding of a repertoire of action types^25,50,52^. In comparison to these proposals, our proposal of action symbols places a greater emphasis on categorical structure (supported by systematic tests using morphed stimuli) and recombination (supported by studying varying sequences, in contrast to previous studies that used a few well-practised sequences^53^). Our findings raise the possibility that the involvement of PMv in diverse motor behaviours—including possibly speech^54^—may reflect diverse types of action symbols.

PMv is at a unique intersection of motor, cognitive and sensory circuits. Like the other macaque premotor areas, PMv is connected with M1 and the spinal cord^25^, which affords it direct control over motor output. In contrast to other premotor areas, PMv is substantially interconnected with PFC (especially vlPFC)^55^, consistent with PMv being involved in cognitive processes. PMv is also interconnected with preSMA^25,55^, an area involved in action sequencing^42^, and together with vlPFC and preSMA, overlaps areas of the human ‘multiple-demand’ system for abstract problem solving^56^. PMv receives input from the two main cortical visual pathways^25^: (1) spatial-related and action-related ‘dorsal stream’ signals via the inferior parietal cortex and (2) shape-related and object-related ‘ventral stream’ signals from the inferotemporal cortex via vlPFC. Although ventral stream signals can encode shape parts^57^, they are unlikely to fully explain symbol-encoding activity in PMv, as we found that PMv preferentially encodes action rather than vision (see above). Processes in PMv may be critical, including a winner-takes-all competition between different symbol-encoding states, consistent with the slow activity dynamics we found for ambiguous images^41^ (Fig. 5h). Future studies are needed to elucidate how diverse inputs interact with local processing in PMv to implement action symbol representations.

### Motor behaviour as a model system for compositionality

Our study introduced a task for studying compositional generalization, with three important methodological features: free choice, stimulus ambiguity and flexible stimulus design. First, subjects must decide their own actions (no movement cues) and cannot memorize solutions (highly varied, including novel, images). This free choice, coupled with an inductive bias for knowledge compression^3,5,9,10^, incentivized the macaques to learn generalizable motor concepts (action symbols). This approach is in contrast to previous animal studies that used drawing-like tasks, which involved direct instruction to track a moving cue^58^ or just a few images^59^. Second, our images were often ambiguous in how they could be interpreted using primitives, which was useful for revealing the nature of each subject’s prior knowledge. Third, our stimulus design was flexible in that it allowed parametric variation of images to test invariance, categorical structure and recombination. Together, these features model a critical aspect of naturalistic behaviour, that is, the need to generalize to situations in which behaviour is not uniquely determined by sensory inputs. In such cases, behaviour requires decisions involving multiple interacting processes, including perception, reasoning and planning, constrained by structured knowledge, including of symbols and how they can be recombined.

This drawing task complements tasks used in previous neural studies of motor behaviour, which have generally not directly addressed compositional generalization. Indeed, behaviours used in those studies tend to fall into the following classes: (1) highly practised, which involves sequences learned through extensive repetition^36,39,60,61^; (2) instructed, which involves cues that signal the correct order of movements, either directly (for example, cues whose location signals reach targets)^43,60,62,63^ or through rules^28,64^; (3) working-memory-guided, which involves reproduction from short-term memory^27,42,60,61^; or (4) minimally restrained, which involves spontaneous production, often in a naturalistic setting^36,37,52^. These overlapping classes expand on a previous classification scheme^60^. Previous drawing-like tasks in monkeys can be classified as highly practised^59^ or instructed^58^.

Behavioural studies have suggested that continuous movements decompose into reused segments called ‘motor primitives’^65^. Compared with action symbols, motor primitives are at a lower level of abstraction, often defined as muscle co-activity patterns (synergies)^65^. An open question is whether they are encoded in the brain (evidence exists in M1 ^43,62,63,66,67^) or are instead by-products of biomechanical constraints, task low-dimensionality or spinal circuit properties^65^. Related studies have identified reused segments (or syllables) in minimally restrained behaviours^17,36,37^. Future studies are required to address how motor primitives and syllables relate to action symbols.

### Bridging symbols and neural computation

Our findings may help bridge two prominent theoretical frameworks of cognition: one based on symbols and rules^2–7,13^ and the other on neural network (connectionist) architectures and dynamical systems^18–20^. Explaining cognition may depend on their unification, possibly by studying whether the brain implements symbolic programs^3,5,9,10,28^ in its neural representations and dynamics^4,13,28,68^, using insights from task-optimized network models^18,19^. Building on our results, future studies could test for activity consistent with symbolic programs in PMv and connected areas, including PFC^6,27,28^, preSMA^42^ and hippocampal circuits implicated in generating compositional representations^4,21,22,30,31,32^.

## Methods

### Subjects and surgical procedures

Data were acquired from two adult male macaques (*Macaca mulatta*, average weight of 17 kg (subject 1 (S1)) and 10 kg (S2), average age of 9 years (S1) and 7 years (S2)). This sample size was chosen to match the standard for neural recording studies of behaviour in monkeys^22,24^. All animal procedures complied with the NIH Guide for the Care and Use of Laboratory Animals and were approved by the Institutional Animal Care and Use Committee of the Rockefeller University (protocol 24066-H).

After undergoing initial task training in their home cages, the subjects underwent two surgeries: the first to implant an acrylic head implant with a headpost and the second to implant electrode arrays. Both surgeries followed standard protocols, including for anaesthetic, aseptic and postoperative treatments. In the first surgery, a custom-designed MR-compatible Ultem headpost was implanted, surrounded by a bone cement cranial implant, or a headcap (Metabond, Parkell and Palacos, Heraeus), which was secured to the skull using MR-compatible ceramic screws (Rogue Research). After a 6-month interval, to allow bone to grow around the screws and for the subject to acclimate to performing the task during head fixation via the headpost, we performed a second surgery to implant 16 floating microelectrode arrays (32-channel FMA, Microprobes for Life Science) using standard procedures^69^. In brief, after performing a craniotomy and durotomy over the target area, arrays were inserted one by one stereotactically while held at the end of a stereotaxic arm with a vacuum suction attachment (Microprobes). Using vacuum suction enabled us to release the arrays, after insertion, with minimal mechanical perturbation by turning off the suction. After all arrays had been implanted, the dura mater was loosely sutured and covered with DuraGen (Integra LifeSciences). The craniotomy was closed with bone cement.

We used standard density arrays (1.8 mm × 4 mm) for all areas, except SMA and preSMA, for which we used four high-density arrays (1.6 mm × 2.95 mm). Four additional electrodes on each array served as the reference (two electrodes) and the ground (two electrodes). Two arrays each were targeted to multiple areas of the frontal cortex, with locations identified stereotactically, and planned using brain surface reconstructions derived from anatomical MRI scans (3D Slicer 5.6.2). Locations were selected on the basis of their published functional and anatomical properties (see below), anatomical sulcal landmarks and a standard macaque brain atlas^70^. During surgery, locations were further adjusted on the basis of cortical landmarks and to avoid visible blood vessels. Arrays were implanted in the right hemisphere (contralateral to the arm used for behaviour).

Array locations are depicted in Fig. 3 and Extended Data Fig. 6 and confirmed with intraoperative photographs. For M1, we targeted hand and arm representations (F1) directly medial to the bend of the central sulcus (which corresponds roughly to the intersection of the central sulcus and the arcuate spur if the latter was extended caudally), based on retrograde labelling from the spinal cord and microstimulation of M1^71^ and M1 recordings^72^. For PMd, we placed both arrays lateral to the precentral dimple, with one (more caudal) array directly medial to the arcuate spur (the arm representation^71–73^, F2), and the other more rostral (straddling F2 and F7). For PMv, we targeted areas caudal to the inferior arm of the arcuate sulcus (F5), which are associated with hand movements based on retrograde labelling from the spinal cord^71^ and M1^74^, microstimulation^75^ and functional studies^51,76,77^ and with decision making^77,78^. These areas contain neurons interconnected with PFC^74^. For SMA (F3) and preSMA (F6), we targeted the medial wall of the hemisphere, with the boundary between SMA and preSMA defined as the anterior–posterior location of the genu of the arcuate sulcus, consistent with previous studies finding differences across this boundary in anatomical connectivity (for example, direct spinal projections in SMA but not preSMA^79^) and function^42,80^. SMA arrays were largely in the arm representation^79^. For dlPFC, we targeted the region immediately dorsal to the principal sulcus (46d), following previous studies of action sequencing^27,28,81^ and other cognitive functions^82^. For vlPFC, we targeted the inferior convexity ventral to the principal sulcus, with one (more rostral) array directly ventral to the principal sulcus (46v) and the other rostral to the inferior arm of the arcuate sulcus (45A/B) based on evidence that encoding of abstract concepts occurs in regions that broadly span these two locations^29,83,84^, including a possibly heightened role (compared with dlPFC) in encoding abstract concepts in a manner invariant to temporal or spatial parameters^84–86^. For FP, we targeted a rostral location similar to previous recording and imaging studies (one array fully in area 10, the other straddling areas 9 and 10)^87,88^, including areas associated with executive functions^89^. In general, array locations targeted the cortical convexity immediately next to sulci, instead of in the banks, to allow shorter insertion depths that minimize the risk of missing the target or damaging blood vessels. The exceptions were SMA and preSMA in the medial wall, for which this was not possible. To avoid damaging the superior sagittal sinus, we positioned the arrays laterally (2 mm from midline) and slanted the electrodes medially (Extended Data Fig. 6).

The lengths of each electrode were custom designed to target half-way through the grey matter and to substantially vary across the array to maximize sampling of the cortical depth. The following electrode lengths were used (in mm): 1.5−3.5 (M1), 1.5−3.1 (PMd and PMv), 2.8−5.8 (SMA and preSMA), 1.5−2.5 (dlPFC and vlPFC) and 1.5−2.6 (FP) for S1; and 1.7−3.75 (M1), 1.5−3.3 (PMv), 1.5−3.1 (PMd), 2.65−5.95 (SMA and preSMA), 1.75−3.15 (dlPFC), 1.35−3.2 (vlPFC) and 1.6−2.9 (FP) for S2. Reference electrodes were longer (6 mm) to anchor the arrays. All electrodeswere Pt/Ir (0.5 MΩ), except 4, which were Ir (10 kΩ). Array connectors (Omnetics, A79022) were housed in custom-made Ultem pedestals (Crist), which were secured with bone cement onto the cranial implant. Four pedestals were used per subject, holding 5, 5, 4 and 2 connectors each.

### Behavioural task

#### Task overview

The subjects were seated comfortably in the dark with their head restrained by headpost fixation. They faced a touchscreen (Elo 1590L 15-inch E334335, PCAP, 768 × 1,024 pixels, refresh rate of 60 Hz, with a matte screen protector to reduce finger friction) that presented images and was drawn on. The touchscreen location was optimized to allow each subject to easily draw at all relevant locations on the screen (23–26 cm away; diagram in Extended Data Fig. 1). Both subjects decided on their own over the course of learning to perform the task with the left hand. The chairs were designed to minimize movements of the torso and legs (by using a loosely restricting ‘belly plate’) and the non-drawing arm (by resting on the belly plate and having movement restricted to within the chair). Gravity-delivered reward (water–juice mixture) was controlled by the opening and closing of a solenoid pinch valve (Cole-Parmer, 1/8-inch inner diameter). The subjects were water-regulated, with careful monitoring to ensure that consumption met the minimum requirement per day (typically exceeding it), and body weight was closely monitored to ensure good health. The task was controlled with custom-written software using the MonkeyLogic (v.2.2.45) behavioural control and data acquisition MATLAB package^90^ (PC: Windows 10 Pro, Intel Core i7-4790K, 32GB RAM; DAQ: National Instruments PCIe-6343). All stimuli (images of line figures defined as point sets, with points rendered large enough to appear as continuous curves) were also generated with custom-written MATLAB (R2021a) code. Images were presented in a workspace area on the screen (16.6 cm × 16.9 cm, corresponding to approximately 37° by 38° visual angle). Shape components in images were on average 4.0 cm (9°) (maximum of width and height).

Each recording session consisted of 2−3.5 h of recording. We collected 5–20 trials per condition (that is, each unique image for Figs. 4 and 5 and the single-shape task in Fig. 6, and each primitive stroke for the character task in Fig. 6). All trials were shuffled across all conditions in the session and presented in a randomly interleaved fashion, except for one case, the experiment in Fig. 6, in which character and single-shape tasks were switched in blocks.

#### Early training

Before surgery, the naive subjects underwent initial training on core task components (that is, to trace images accurately using a sequence of discrete strokes). Early training took place in the home cage using custom-built rigs that were attached to an opening in the cage using the same hardware and software described above, except for the computer (Lenovo IdeaPad 14-inch laptop, Windows 10, AMD Ryzen 5 3500U, 8GB RAM) and DAQ (National Instruments USB-6001). This initial training progressed through seven stages. (1) Touch circle. The subjects were rewarded for touching a circle anywhere within its bounds. The circle started large, filling the entire screen, and shrank over trials to enforce more accurate touches. (2) Touch with a single finger. We shrank the circle until it was so small that it could only be touched with a single finger. The trial aborted if the subject touched outside the circle or with multiple fingers simultaneously. (3) Hold still. The subjects were rewarded for keeping their fingertip still on a dot, with the duration of this hold increasing across trials for up to a few seconds. (4) Track moving dot. The subjects had to track the dot with their finger as it moved (a lag between dot and finger was allowed). (5) Trace a line. We increased the speed of the moving dot over trials until eventually the dot moved so fast that the line it traced immediately appeared. We then positioned the line at locations far from the hold position to train the subject to raise its finger from the hold position and to trace lines at arbitrary locations, angles and lengths. (6) Trace single shape. We presented shapes of increasing difficulty (gradually morphing across trials from a straight line), including arcs, L-shapes, squiggles and circles. Across these stages, the shapes were presented at random locations. We did not enforce any particular tracing trajectory for each shape, which allowed the subjects to choose on their own. (7) Trace multiple shapes. We presented images composed of multiple disconnected shapes. This trained the subjects to understand that they should use multiple strokes to trace multiple shapes. At this point, the subject understood the basic structure of the task: to trace shapes using multiple strokes if needed. The progression across these stages was not determined by strict quantitative criteria but instead on a combination of quantitative and qualitative evaluations of task performance.

The subjects then practised various tasks to incentivize the learning of stroke primitives (consistent stroke trajectories for each shape). They practised single-shape trials using the set of diverse simple shapes in Fig. 1e, varying randomly in shape and location across trials. We chose this set of shapes to cover a range of trajectory profiles (by varying rotation, the number of direction changes and whether shapes were curved or linear) and yet were simple enough to draw with one stroke and to combine multiple (two to six) shapes into single-character images. We did not constrain the subjects to learn specific stroke trajectories for each primitive; therefore, differences between primitives reflected each subject’s own learning trajectory for how to draw each shape. Note that our study did not depend on using an optimal set of shapes or primitives but instead depended on learning primitives and then demonstrating behavioural generalization using these primitives, as we found for our subjects. For S2, the four S-shapes and four arc shapes were also sometimes presented as rectilinear versions. For S-shapes these resembled Z, and for arc these resembled squares missing one edge. S2 drew these rectilinear versions using curved strokes similar to those used for S and arc shapes. Therefore we combined data for each rectilinear shape with its respective curved version. On different days, the subjects also practised multishape and character tasks.

#### Trial structure

Trials (event sequence in Fig. 1c, screen schematic in Extended Data Fig. 1c) began when the subject pressed and held a finger fixation button (blue square) at the bottom of the screen (note that button always means a virtual button). After a random delay (uniform, earliest 0.4–0.6 s and latest 0.8–1.0 s across experiments for S1 and 0.8–1.1 s for S2) the image appeared (dark grey on a light-grey background). After a random delay (uniform, ranging from 0.6–1.0 s to 1.2–1.6 s across experiments for S1, and 1.1–1.5 s to 1.8–2.4 s for S2), a go cue (400 Hz tone and image blank for 300 ms) was presented. During this delay between image presentation and the go cue (planning epoch), the finger had to be kept still on the fixation button, but the subject was free to look anywhere. After the go cue, the subject was free to move their hand towards the image and start drawing. Immediately after the finger was raised from the fixation button, it disappeared and a ‘done button’ (green square) appeared at its location and stayed there. During drawing, the image stayed visible and the finger left a trail of black ‘ink’ on the screen. The subject signalled drawing completion by pressing the done button (effectively no time limit was imposed). This was followed by performance feedback, which spanned four modalities, each signalling performance: (1) screen colour, (2) sound, (3) duration of delay before getting reward (time out) and (4) reward. First, screen colour and sound were signalled, followed by the time out, and then the reward (detailed below). In addition to this feedback at the end of the trial, we also provided online feedback by immediately aborting the trial in case of serious errors; for example, touching far from any image points or for single-shape and multishape trials (but not for character trials), using more than one stroke per shape component. These online abort modes were turned off for trials testing novel characters.

Screen image changes (including image presentation and other trial events) were recorded using photodiodes (Adafruit Light Sensor ALS-PT19), and sounds were recorded using an electret microphone (Adafruit Maxim MAX4466, 20-20KHz). We performed eye tracking (ISCAN), but did not enforce eye fixation.

### Scoring behavioural performance

Behaviour was scored by aggregating multiple metrics or factors. There were three classes of factors. The factors that had the greatest influence on the final aggregate score measured image similarity, or the similarity of the final drawing to the target image (ignoring its temporal trajectory). We also computed factors that reflected behavioural efficiency and, in some cases, factors that were task-specific. These scores were computed using behavioural data (a sequence of touched *xy* coordinates with gaps between strokes) and image data (a set of *xy* coordinates). Below, we describe the factors and then how they were aggregated into a single score.

#### Image similarity

This included two factors: drawing-image overlap and Hausdorff distance. Drawing-image overlap was the fraction of the image points that were touched (within a margin of error) by at least one of the drawn points. A subset of the image points were weighted more heavily because they captured characteristic features of the shape (for example, the corners and end points of an L-shape). Hausdorff distance is a metric used to measure the distance between the set of drawn points and the set of image points (definition below).

#### Behavioural efficiency

To incentivize efficiency, we included a factor that compares the cumulative distance travelled in the drawing (that is, the amount of ink) to the cumulative distance of the edges of the figure in the image, with its value negatively proportional to the excess of drawn ink over image ink.

#### Task-specific factors

During practice trials for the character task (see the section ‘Task types’), we also included factors that capture the extent to which drawn strokes matched the shapes used in the image. This included two factors: one proportional to the similarity of the number of strokes and the number of image shapes, and the other proportional to the spatial alignment of the drawn strokes to the image shapes. Importantly, these factors were included only for practice images and not for novel test images.

The final score aggregated the image similarity, behavioural efficiency and task-specific factors, with more weight on image similarity factors. We first rescaled factors linearly between 0 and 1 (where 1 means good performance), with the dynamic range set by a lower and upper bound. These bounds were adaptively updated on every trial based on the distribution of factor values in the last 50 trials (lower bound set to the 1st percentile and upper bound to the 53rd percentile), which ensured that the dynamic range of feedback matched the dynamic range of behavioural performance from recent history. We then weighted each factor to tune its relative contribution (using weights hand-tuned for each experiment; generally highest for image similarity) and computed the final scalar score (range 0 to 1) using the worst factor after weighting:

$${s}_{\mathrm{scal}}=\mathop{\min }\limits_{i}(1-{w}_{i}(1-{f}_{i}))$$

where *i* indexes the factors, *w*_*i*_ are the weights (between 0 and 1) and *f*_*i*_ are the factor values (between 0 and 1). We also gave each trial a categorical score: great (*s*_scal_ > 0.82), good (0.65 < *s*_scal_ ≤ 0.82), OK (0.15 <* s*_scal_ ≤ 0.65) or fail (*s*_scal_ ≤ 0.15).

The scalar and categorical scores determined the feedback across the four different modalities. The meaning of screen colour and sound were learned, whereas delay and reward had intrinsic value. For screen colour, a linear interpolation between two colours, such that a score of 0 was mapped to red (RGB: 1, 0.2, 0) and 1 was mapped to green (0.2, 1, 0.2). For sound cue, a sound was determined by *s*_scal_: if great, then three pulses (1,300 Hz, 0.16 s on and off); if good, then a single pulse (1,000 Hz, 0.4 s); if OK, then no sound; if fail, then a single pulse (120 Hz, 0.27 s). For delay until reward, a nonlinear mapping was generated from score to delay before reward. We first applied a linear mapping from the scalar score, such that a score of 0 was mapped to a long delay (5 s + a random uniform jitter of 0–2.5 s), and a score of 1 was mapped to 0 s of delay. Furthermore, if *s*_scal_ was great, good or OK, this delay was reduced by multiplying by 0.65. Finally, for reward, the open duration of the solenoid gating the juice line was defined as

$${\rm{r}}{\rm{e}}{\rm{w}}{\rm{a}}{\rm{r}}{\rm{d}}={C}\times m\times a\times {s}_{{\rm{s}}{\rm{c}}{\rm{a}}{\rm{l}}}$$

where *C* is a constant in dimensions of time (0.15−0.6 s, manually set depending on the difficulty of the task); *m* is a multiplier that gives a bonus for good performance and further penalizes bad performance, depending on the value of *s*_scal,_ great (1.3), good (1.0), OK (0.8) or fail (0); *a* is a random variable sampled from the uniform distribution *a* ~ 0.75 + 0.5 ×* U*(0,1); and *s*_scal_ is defined as above. On average, including failed trials, the subjects received around 0.35 ml reward per trial. The temporal order in which these four feedback signals were delivered is described above (see the section ‘Trial structure’).

### Task types

#### Single-shape task

The single-shape task presented one of the practised simple shapes or, in the categories experiment, sometimes a morphed shape. The subjects were only allowed to use a single stroke (triggering online abort if more was used). In four single-shape sessions for S1, the ending of the drawing epoch was triggered by stroke completion (that is, on finger raise) not on the pressing of the done button as in all other sessions and experiments.

To test for motor invariance (Figs. 2a–e and 4), we presented images of practised shapes, varying across trials in location, size or both. For location variation, images spanned 321 pixels (9.6 cm) in the *x* and *y* dimensions (measuring between shape centres), which is 2.38 times the average size of shapes (135 pixels, 4.0 cm, maximum across width and height). For size variation, the maximum size was 2.5 times larger than the smallest (in diameter), except for two experiments for S1, in which the ratio was 2.0. The location and size variation in Fig. 2b,c is representative (S1, *n* = 2 sessions varying in location, *n* = 3 varying in size; S2, *n* = 3 sessions varying in location, *n* = 2 varying in size).

Size and location variation was chosen based on previous studies of M1 activity and electromyography of muscles controlling the arm during reaching in macaques^91,92^. For reaches performed along the coronal plane around 25 cm from the subject, similar to the geometry of the touchscreen relative to the subject in our study, M1 and electromyographic activity are substantially affected by translating the reach location by 10 cm (ref. ^91^), similar to the location variation in our study (9.6 cm), and by varying the scale of the reach by 2-fold (7 cm to 12 cm)^92^, similar to the size variation in our study (2–2.5-fold).

To test for categorical structure (Figs. 2f–j and 5), we constructed morph sets (S1, *n* = 7 morph sets across 3 sessions; S2, *n* = 13 morph sets across 4 sessions), each consisting of two practised shapes and four to five images that morph between those shapes through linear interpolation along shape parameters, such as the extent of closure of the top of the U (Fig. 2g). Across morph sets, we varied different image parameters (Extended Data Fig. 3).

#### Multishape task

Each image was composed of two to four shapes positioned at random, nonoverlapping, locations spanning the space of the screen (possible locations include the four corners and the centre). The subjects were allowed to draw the shapes in any order and to use any trajectory for each shape, but were constrained to use one stroke per shape and to not trace in the gaps between shapes. We present results averaged across two sessions, one from each subject (Extended Data Fig. 4). For each trial, an image was constructed by sampling shapes randomly without replacement. This led to *n* = 531 (S1) and *n* = 278 (S2) unique images.

#### Character task

Each image was generated by connecting two to six simple shapes into a single character by sampling from a generative model as follows. A character with *N* shapes was defined by randomly sampling *N* shapes and *N* – 1 relations, where each relation (indexed *i*) defines the locations of the attachment points on shapes *i* and *i* + 1, which in turn define how the shapes connect to each other. This approach is similar to a previous generative model for handwritten characters^9^. Generated characters were only kept if there was minimal crossing of shapes over each other.

For experiments testing behavioural generalization to novel characters (Fig. 2k–p), we mixed practised and novel characters (practised, *n* = 189 (mean, range 22–491) per day; novel, 48 (mean, range 0–155) per day). For analyses, we labelled as ‘novel’ only the very first trial for a given character. Because of random sampling in generating characters, it would in principle be possible that characters generated on different days are in fact identical. To avoid this possibility, we ensured post hoc that all characters labelled novel were different from every previously encountered character across all days (quantified using the Hausdorff distance).

For neural experiments comparing single-shape and character tasks (Fig. 6), we analysed the sessions for which we collected data from both the single-shape and character tasks (S1, *n* = 9 sessions, median *N* matching primitives between single-shape and character tasks = 9 (range 5−12); S2, *n* = 9 sessions, median* N* matching primitives = 10 (range 2−14)). We switched between single-shape and character tasks using a block design (2−5 blocks each per session), except one session for S2, which used random interleaving across trials.

### Behavioural data analysis

#### Preprocessing of touchscreen data

Touchscreen data were represented as time series of (*x*, *y*) coordinates in units of pixels (conversion: 33.6 pixels per cm) and sampled at 60 Hz, which we upsampled to 500 Hz (performed in MonkeyLogic to align all behavioural signals, including trial event markers and eye tracking) and low-pass filtered to keep only drawing-related movements (15 Hz). Strokes were segmented based on the time of first touch (onset) and the time of last touch (offset) with 500 Hz resolution.

For some analyses (Fig. 1f and as input to the trajectory distance below) we further computed stroke instantaneous velocity and speed as follows. Extracted strokes were low-pass filtered (12.5 Hz) and downsampled to 25 Hz. We then used the standard five-point stencil method to compute a finite difference approximation of the derivative (separately for the *x* and *y* coordinates):

$${f}^{{\prime} }[n]=\frac{f[n-2]-8f[n-1]+8f[n+1]-f[n+2]}{12\,{\mathrm{h}}}$$

where *f*[*n*] is a discrete time series (that is, the *x* or *y* coordinates) indexed by integer *n*, and *h* is the sampling period in seconds. The resulting velocity time series was upsampled to the original 500 Hz sampling rate with a cubic spline. Speed was computed as the norm of the (*x*, *y*) velocity at each time point.

#### Computing the trajectory distance

To quantify the similarity between any two strokes based on their spatiotemporal trajectories while ignoring their relative size and location, we devised a trajectory distance metric, a scalar dissimilarity score based on the dynamic time warping distance between two strokes represented as velocity time series *v*_1_ and *v*_2_. To compute trajectory distance between two strokes, we spatially rescaled each stroke (while maintaining its *xy* aspect ratio) to make the diagonal of its bounding box unit length 1. We then linearly interpolated each stroke to the same number of points (70) to allow point-by-point comparison between strokes. This was done spatially by interpolating based on fraction of cumulative distance travelled (so that the distances between successive points were the same over the entire stroke) to capture the spatiotemporal trajectory, as in a previous analyses of strokes in handwriting^9^. Interpolated trajectories were converted to velocity time series as described above. We then computed the dynamic time-warping distance between velocities *v*_1_ and *v*_2_:

$${D}_{\mathrm{DTW}}({v}_{1},{v}_{2})=\frac{{\mathrm{min}}_{{\rm{\pi }}}{\sum }_{(i,j)\in {\rm{\pi }}}d(i,j)}{{\mathrm{N}}}$$

where *i* and *j* index the two velocity trajectories, *N* is the number of points (70), and π is a set of (*i*, *j*) pairs representing a contiguous path from (0, 0) to (*N*, *N*). The local distance metric *d*(*i*, *j*) is the Euclidean distance plus a regularization factor to discourage excessive warping:

$$d(i,j)=|{v}_{1}[i]-{v}_{2}[\,j]|+\lambda \times |i-j|,$$

$$\lambda =0.045{\langle |{v}_{n}[i]|\rangle }_{i,n}.$$

For the regularization parameter, *λ*, the purpose of the summation term was to rescale it to match the magnitude of velocities. The resulting distance *D*_DTW_ was then rescaled between 0 and 1 to return the trajectory distance:

$${D}_{\mathrm{traj}}({v}_{1},{v}_{2})=1-\frac{1}{{D}_{\mathrm{DTW}}({v}_{1},{v}_{2})+1}.$$

#### Computing the image distance

To compare the similarity of two images—each a set of (*x*, *y*) points—we used a modified version of the Hausdorff distance, a distance metric commonly used in machine vision for comparing the similarity between two point sets based on shape attributes^93^. There are, in principle, at least 24 variants of the Hausdorff distance based on possible formula variations^93^. Here we used a variant that is minimally susceptible to outlier points because it takes means instead of minima and maxima (variant 23 in the referenced study^93^). Image distance was computed as follows: (1) each image was centred so its centre of mass was at (0, 0); (2) the image distance was then computed. First, we defined the distance between two points, *d*(*a*, *b*), as the Euclidean distance. We also defined the distance between a point and a set of points, *d*(*a*, *B*), and the distance from set *A* to set *B*, *d*(*A*, *B*), as follows:

$$d(a,B)=\mathop{\min }\limits_{b\in B}\,d(a,b),$$

$$d(A,B)=\frac{1}{|A|}\sum _{a\in A}d(a,B).$$

The image distance was then defined as:

$${D}_{\mathrm{image}}(A,B)=\frac{d(A,B)+d(B,A)}{2}.$$

#### Computing the primitive alignment score

For experiments on categorical structure, we generated a set of images with each set containing four to five novel images that morph between one primitive (P1) and another primitive (P2) to create a morph set. Each trial presented a single image from one morph set. We sought to quantify the relative similarity between the data of a given trial—its behavioural, image or neural data (see below)—and data for the two primitives, P1 and P2, in its morph set. To do so, we devised a primitive alignment score defined as:

$$a=\frac{{d}_{1}}{{d}_{1}+{d}_{2}}$$

where *d*_1_ is the average of the distances between a given trial and each of the P1 trials, and *d*_2_ the average distance to the P2 trials. A score closer to 0 implies similarity to P1, and a score close to 1 implies similarity to P2 (note that in practice the primitive alignments for P1 and P2 data are not exactly 0 and 1 owing to trial-by-trial variation). The particular metric used for these distances depended on the analysis: image distance (for images), trajectory distance (for drawings) or Euclidean distance between population activity vectors (for neural activity). We confirmed that primitive alignment scores for image data varied linearly with morph number (Fig. 2j and Extended Data Fig. 3c), which ensured that any deviation from linearity in behavioural or neural data could not trivially be the consequence of how the score is defined.

#### Classifying strokes from the character task

To assess whether the subjects drew characters by reusing their own stroke primitives, we scored the fraction of character strokes that were high-quality matches to the subject’s own primitives and the fraction that were high-quality matches to the other subject’s primitives. If the fraction of matches to a subject’s own primitives was high, and to the other subject’s primitives was low, then this was evidence for recombining the subject’s own primitives.

This assessment was done by assigning each stroke the label of its most similar primitive using the trajectory distance and then defining this as a high-quality match only if the trajectory distance was sufficiently low (defined below).

First, each stroke was assigned its best-matching primitive, p*, from a set of primitives (the choice of primitive set—same or different subject—depending on the analysis; see below):

$${p}^{\ast }=\mathop{\mathrm{argmin}}\limits_{p}\,d(s,{\mu }_{p})$$

where *p* indexes the primitives, *s* is the stroke trajectory, *µ*_*p*_ is the mean trajectory for primitive *p* (averaged over trials from the single-shape task), and *d*(.,.) is the trajectory distance.

Second, the quality of the assignment of the stroke to p* was scored as high if $d(s,{\mu }_{{p}^{\ast }}) < {{D}}_{max,{{\mathrm{p}}}^{\ast }}$ and low if $d(s,{\mu }_{{p}^{\ast }})\ge {{D}}_{max,{p}^{\ast }}$, where ${{D}}_{max,{{\mathrm{p}}}^{\ast }}$ is an upper bound on trajectory distances that would be expected from trial-by-trial variation in primitive p*. It is the 97.5th percentile of the distribution of trajectory distances from single-shape trials, determined separately for each primitive, which we consider as a good (arguably conservative) estimate of trial-by-trial variation. This is because the single-shape task presents no ambiguity as to what primitive needs to be drawn and can therefore be considered ‘ground truth’.

These steps assigned each stroke a class tuple (p*,quality), where high quality was interpreted as the stroke matching the primitive set and low quality meant failure to match any primitive. Note that the non-uniformity of the frequency distribution of matches across primitives (Fig. 2o) resembles non-uniformity seen for language and other behaviours^94^. Also, note that even the primitives with a low frequency of match (Fig. 2o) can be considered legitimate matches because they satisfied the criteria to be labelled as high quality.

In summary analyses, we pooled all cases of high-quality matches into a single match class (regardless of the assigned primitive), and all low-quality matches into a single no-match class (Fig. 2p). To test whether a given subject’s character strokes aligned better with its own primitives compared with the other subject’s primitives (Fig. 2p), we performed the above analysis separately for all four combinations of stroke data (2 subjects) × primitives (2 subjects) using only images drawn by both the subjects.

The control analysis of testing primitive reuse using a simulated set of remixed primitives was performed as follows. We generated remixed primitive sets by mixing subparts of different primitives. Given two actual primitives, we extracted the first half of the first primitive (defined by the distance travelled) and the second half of the second primitive and connected them by aligning the offset of the first half to the onset of the second half, smoothing the connection with a sigmoidal weighting function. To sample a remixed primitive set, we first generated a pool of all possible remixed primitives using every possible pair of actual primitives. We then sampled (without replacement) a set of remixed primitives from this pool, keeping only remixed primitives that satisfied the following constraints: (1) no self-intersection, and (2) no excessively sharp turns, detected as curvature at any point along the inner 80% of the stroke exceeding 0.8. Curvature was defined in a standard manner as the inverse of the radius of curvature,

$$\frac{\dot{x}\ddot{y}-\dot{y}\ddot{x}}{{({\dot{x}}^{2}+{\dot{y}}^{2})}^{\frac{3}{2}}}$$

where $\dot{x}$ and $\dot{y}$ are the velocity components and $\ddot{x}$ and $\ddot{y}$ are the acceleration components. Once a candidate set of remixed primitives was sampled, it had to pass further constraints: (1) each actual primitive contributed its first half or second half to a maximum of two remixed primitives; (2) the trajectory distance between no pair of remixed primitives was lower than the minimum trajectory distance between actual primitives; and (3) the trajectory distance between no pair of remixed primitive and actual primitive was less than the minimum trajectory distance between actual primitives. These constraints ensured that remixed primitives in each set were different from each other and from the actual primitives (visually apparent in Extended Data Fig. 5).

We then labelled strokes from character drawings using each remixed primitive set using the same approach as for actual primitives, except the following. The analysis using actual primitives used trajectory distance thresholds (${{D}}_{max,{{\mathrm{p}}}^{\ast }}$) determined empirically for each primitive based on single-shape trials (see above). Because remixed primitives were never drawn, a similar approach could not be used. Instead, thresholds for remixed primitives were assigned from the pool of thresholds for the actual primitives, in a manner meant to increase the stroke–primitive match rate (and thus allow a stronger, or more conservative, test that the remixed primitives do not match the character strokes) by assigning the largest (most lenient) threshold to the worst-matching remixed primitive, the second largest to the second worst, and so on, where worse means having larger average trajectory distance to character strokes.

#### Analysis of kinematic separability of primitives

To test whether each primitive was decodable from every other primitive based on single-trial kinematics (stroke trajectory) (Extended Data Fig. 2), strokes were first converted from time series (*x* and *y* position) to eight-dimensional vectors as follows. Similar to the trajectory distance computation (above), strokes were normalized in time (linear interpolation to 50 time points) and space (rescaling each stroke, while maintaining its *xy* aspect ratio, to make the diagonal of its bounding box unit length 1). The *x* and *y* coordinates were concatenated to a 100-dimensional vector and then reduced to 8 dimensions using principal component analysis (PCA). Decoding was performed on these 8D representations using a linear support vector machine classifier (SVC) (LinearSVC, scikit-learn (v.1.3.0), regularization parameter C set to 0.1), with 10-fold cross-validation. By performing this procedure for every pair of primitives (each time returning a single decoding accuracy score), we populated the matrix shown in Extended Data Fig. 2.

### Neural recordings

Recordings were acquired using a Tucker-Davis Technologies (TDT) system, including headstage (Z-Series 32 Channel Omnetics, LP32CH-Z), amplifier (PZ5M-256), processor (RZ2) and storage (RS4), sampled at 25 kHz (local reference mode), controlled with TDT Synapse (v98) software run on a Windows 10 PC (Intel Core i7-3770, 32GB RAM) and saved to disk. Analog and digital task-related signals, including behavioural events (photodiode, audio and trial event markers) and eye tracking (ISCAN, 125 Hz), were synchronized to external triggers recorded by the neural data acquisition system.

### Neural data preprocessing

#### Spike sorting

We extracted for later analysis both single-unit (SU) and multiunit (MU) spike clusters from the stored broadband signal as follows. MU clusters consisted of identified spikes, which were not isolatable into distinct SU clusters. We used a three-step approach for extracting and clustering spikes, with a first pass using Kilosort (v.2.5)^95^ to extract putative spike clusters, a second pass using a custom-written program to label these clusters as SU, MU or noise, and a final manual curation step. Although Kilosort classifies clusters, we did not use those labels.

For Kilosort, we used default parameters, except AUCsplit (0.90), Th ([6, 4]) and lam (10), which we optimized using parameter sweeps on data from representative sessions and by manual evaluation of results.

We next refined cluster labels. For each cluster, we removed outlier waveforms (exceeding a 3 times interquartile-range threshold for any of the minima, maxima or sum-of-squares). Waveforms were then shifted slightly in time if needed (<1 ms) to improve their alignment by troughs (or peaks, for positive-going waveforms). For each cluster, we computed two features: the signal-to-noise ratio (SNR) and inter-spike-interval violations (ISIVs). The SNR was defined as the ratio of the peak-to-trough difference (of the average spike waveform) tothe standard deviation (averaged across time bins). If a cluster contained both positive-going and negative-going waveforms, the SNR was computed separately for these two subsets of data and then averaged. ISIVs were defined as the fraction of inter-spike intervals less than a refractory period (1.5 ms). On the basis of these SNR and ISIVs, we provisionally classified clusters as SU (if either (SNR > 9.6 and ISIV < 0.05) or (SNR > 6.9 and ISIV < 0.01)), noise (SNR < 3.9) or MU (the remaining clusters).

We then manually curated these clusters. We visualized waveforms for every cluster to either confirm its label (MU, SU or noise) or to manually re-assign it to a different label (including artefact) using a custom-written MATLAB GUI. We also manually checked whether to merge multiple SU clusters on a single channel into a single SU cluster if they exhibited high waveform similarity, inversely correlated spike count frequency over the course of the session or a negative peak close to zero lag in a cross-correlogram of spike times. Finally, for each channel, all MU clusters were merged into a single MU cluster. Combining SU and MU, this process produced the following number of units per area (mean ± s.d. across sessions) for the different brain regions: for S1, M1 (59.9 ± 12.5), PMd (44.1 ± 6.2), PMv (34.2 ± 7.3), SMA (63.0 ± 7.9), preSMA (75.4 ± 17.7), dlPFC (47.8 ± 17.2), vlPFC (43.3 ± 9.9) and FP (19.2 ± 3.8); for S2, M1 (40.7 ± 13.1), PMd (54.7 ± 5.5), PMv (71.1 ± 6.6), SMA (53.4 ± 7.2), preSMA (57.9 ± 11.1), dlPFC (24.6 ± 4.8), vlPFC (38.6 ± 13.7) and FP (42.6 ± 5.0).

#### Converting spike times to firing rates

Single-trial spike trains were converted to firing rate functions by smoothing with a 0.025 s Gaussian kernel (0.01 s slide). We removed units with very low firing rates (if the 80th percentile of their firing rates across all trials and time bins was less than 1 Hz). We also removed units for which the firing rates were unstable, either due to high systematic drift in the firing rate over the session (*m*/*u* > 0.2, where *m* is the slope from regressing the square-root-transformed firing rate versus time (in hours), and *u* is the mean firing rate) or due to large fluctuations in the firing rate across the session. For the latter, we excluded units satisfying either (*s*_max_ – *s*_min_)/*s*_mean_ > 1.15 or (*u*_max_ – *u*_min_)/*u*_mean_ > 0.65, where the session is first split into contiguous 50-trial bins, the across-trial standard deviation in the square-root-transformed firing rate is computed in each bin, and then *s*_max_, *s*_min_ and *s*_mean_ are defined as the maximum, minimum and mean standard deviation across bins, respectively; *u*_max_, *u*_min_ and *u*_mean_ are defined similarly, except using the within-bin mean firing rate instead of the within-bin standard deviation. We then processed firing rates as follows. We first square-root transformed activity to normalize its variance. Following a common approach in analyses of population firing rates^96^, we *z*-scored the activity of each unit to ensure that neurons with highly different firing rates contributed similarity to population analyses, but in a ‘soft’ manner so that higher-firing-rate neurons contributing relatively more:

$${x}_{i}^{{\rm{norm}}}(t)=\frac{{x}_{i}(t)-{\mu }_{x}}{{\sigma }_{x}+C}$$

where *x*_*i*_(*t*) is firing rate for trial *i* at time bin *t*; *µ*_*x*_ and *σ*_*x*_ are the mean and standard deviation, respectively (across trials and time bins), and *C* is an additive factor to ensure softness, where *C *= min(*m*) + 3 Hz, where *m* is a vector of mean firing rates across units. All subsequent analyses used this normalized firing rate.

#### Time-warping neural activity to a common trial template

For the figure showing the average firing rates over the entire trial (Fig. 3c), we first time-warped each trial to a common trial template. We defined a set of events that occur across trials as anchors (fixation touch, image onset, go cue, finger raise off fixation, stroke onset, stroke offset, touch done button and reward). We included only single-stroke trials. We first generated a median trial. For each segment (that is, the time window between a pair of successive anchor events), we found its median duration and then concatenated these median segments to construct a median trial. We then aligned each trial to this median trial at the anchor events, warping time linearly in each segment. This warping did not change the firing rate values, just their timing. To avoid sharp discontinuities at anchor points, we smoothed the final firing rates at the times of the anchor points (2.5 ms Gaussian kernel).

Sorting of units (rows) in the resulting firing rate plot (Fig. 3c) was performed in a cross-validated manner. Sort indices were determined using one subset of trials (*n* = 50) and then applied to sort the remaining subset of trials that are plotted (*n* = 235).

### Neural data analyses

#### PCA

We performed dimensionality reduction on the neural population activity, in general because high-dimensional noise can reduce the interpretability of the Euclidean distance^97^, and in one case to identify a potential linear projection of population activity (that is, a subspace) that preferentially encodes primitives, a standard approach^28,98^. We represent data for a single area from a single session and in a specific within-trial time window as a matrix *X* of size *N* × *KT*, where *N*, *K* and *T* are the number of units, trials and time bins, respectively, constructed by concatenating time bins from all trials along the second dimension. Data were first binned in time (0.15 s window, 0.02 s slide) before constructing this data matrix. Instead of applying PCA on single-trial data *X*, we applied PCA on trial-averaged data *X*_*C*_, to minimize the influence of trial-by-trial variation (noise). *X*_*C*_ holds the mean activity for each trial condition, of size *N* × *K*_*C*_*T*, where *K*_*C*_ is the number of unique conditions, with the specific conditions depending on the experiment (see below). We performed PCA on *X*_*C*_ and retained the top eight PCs. The specific trial-averaged conditions used for identifying PCs were the following. For analysis of motor invariance (Fig. 4), the conditions were each unique primitive (averaging over location or size), which resulted in identifying PCs that preferentially encoded primitives if such PCs exist. For analysis of categorical structure (Fig. 5), PCA was performed separately for each morph set, and the conditions were the unique images (that is, the two end point shapes and the morphed shapes in between). For the analysis of primitive representational reuse in characters (Fig. 6), the conditions were each combination of primitive and task type.

We performed PCA in a cross-validated manner to ensure that it was not overfitting to noise. We partitioned trials into two subsets (in a stratified manner): one training set that was used only for identifying the PCs and a test set that was projected onto these PCs and then used for all subsequent analyses. We performed eight randomized train–test splits (including all downstream analyses) and averaged their results.

#### Representing time-varying population activity as a vector

For some analyses, we captured single-trial time-varying population activity during the planning epoch (0.05–0.6 s after image onset) as a vector, which could then be visualized after dimensionality reduction (Extended Data Fig. 7) or used in decoding analyses (Extended Data Fig. 12). Starting from the population data of a region, represented as a *K* × *N* × *T* matrix, where *K*, *N* and *T* are the numbers of trials, units, and time bins, respectively, (with time binned using a 0.2 s window, 0.1 s slide), we concatenated the *N* channels’ length *T* vectors end-to-end to construct the data matrix *D*, of size *K* × *NT*, where each trial is represented by a vector of length *N* × *T*.

#### Nonlinear dimensionality reduction

To perform nonlinear dimensionality reduction of population activity to two dimensions (Extended Data Fig. 7), we used the uniform manifold approximation and projection (UMAP), performed on *D*, using values of 40 and 0.1 for the parameters n_neighbors and min_dist.

### Computing the neural distance

To quantify the similarity of population activity between two sets of trials, such as trials for conditions *A* and *B*, each a specific conjunctive value of task-relevant variables, we devised the ‘neural distance’ metric. Inspired by a previously described ‘normalized distance’^99^, it is the average pairwise Euclidean distance across conditions *A* and *B*, minus the average within-condition distance. This subtraction ensures the useful property that this distance is unbiased, in that the expected value of neural distance between two sets of trials sampled from the same distribution is zero (unlike the mean Euclidean distance, which is biased upwards^26^). Moreover, the resulting distance is normalized by dividing by an upper-bound distance to normalize it between 0 and 1. The neural distance is defined as:

$${D}_{{AB}}^{* }={D}_{{AB}}-({D}_{{AA}}+{D}_{{BB}})/2$$

where the normalized Euclidean distance between sets of trials (indexed by *i* and *j*) in conditions *A* and *B* is:

$${D}_{AB}={\left\langle \frac{1}{{d}_{max}(t)}{\langle |{x}_{i}(t)-{x}_{j}(t)|\rangle }_{i\in A,j\in B}\right\rangle }_{t\in \{{t}_{1},\ldots ,{t}_{n}\}}.$$

Here, *x*_*i*_(*t*) is the population activity vector at time *t* (in a window between times *t*_1_ and *t*_*n*_), and *d*_max_ is an upper bound (98th percentile) of the distances between all pairs of different trials combined across all conditions.

### Computing the encoding strength of a variable

To compute how strongly a given variable is encoded in population activity (for example, primitive encoding in Fig. 4j), we computed the mean effect of that variable on population activity in terms of neural distance while controlling for the other relevant variables. Consider an experiment in which conditions vary along two variables, primitive and location, represented as the tuple (*p*, *l*), where *p* and *l* represent the primitives and locations, respectively. Primitive encoding is the average neural distance across all pairs of conditions that have different primitives but same locations:

$$\text{primitive encoding}\,=\,{\langle {D}_{(p,l)({p}^{{\prime} },{l}^{{\prime} })}^{* }\rangle }_{p\ne {p}^{{\prime} },l={l}^{{\prime} }}.$$

Location encoding is defined analogously:

$$\text{location encoding}\,=\,{\langle {D}_{(p,l)({p}^{{\prime} },{l}^{{\prime} })}^{* }\rangle }_{p={p}^{{\prime} },l\ne {l}^{{\prime} }}.$$

This approach generalizes to any pair of variables, such as primitive and task type in Fig. 6.

### Statistically comparing brain regions in the strength of variable encoding

In analyses that compared the encoding strengths of a particular pair of variables (for example, primitive versus location in Fig. 4j), we used the following procedure to compare each brain region with every other brain region in how strongly they encode these two variables. (1) For each variable and pair of brain regions, we compared the strength of encoding of that variable in these regions. This involved first extracting a dataset of neural distances for each pair of trial conditions for each of the two brain regions. For example, if the variable was primitive, then each of the two brain regions would contribute a dataset consisting of neural distances for all pairs of trial conditions with different primitives but the same location. The datasets for these two regions would be combined and we then fit a linear least-squares regression model to test for an effect of brain region on neural distance *y*, controlling for the effect of trial–condition pair:

$$y={\beta }_{0}+{\beta }_{r}{X}_{r}+\mathop{\sum }\limits_{j=1}^{{N}_{c}}{\gamma }_{j}{Z}_{j}+{\epsilon }$$

where *β*_0_ is the intercept term, *X*_*r*_ is 0 or 1 depending on the brain region, and *Z*_*j*_ is an indicator variable representing the trial–pair condition, with *γ*_*j*_ as their coefficients, and ${\epsilon }$ is a noise term. Finally, we extracted the *P* value for *β*_*r*_ (two-sided *t*-test), which represents the significance of the difference between these two regions in encoding strength for this variable. (2) This procedure was performed once for each combination of variable and brain region pair, which resulted in 56 *P* values, corrected for multiple comparisons using the Bonferroni method (2 variables × 28 brain region pairs = 56 comparisons). (3) Using these 56 *P* values, we summarized each region with 2 numbers representing the number of regions in comparison to which this region more strongly encodes these two variables. For example, for primitive × location experiments, each region was scored with a tuple (*N*_prim_,*N*_loc_), where *N*_prim_ is the number of other regions this region beats in the pairwise tests of primitive encoding (and analogous for *N*_loc_, except for location encoding). In summary plots (Figs. 4j and 6f), the resulting (*N*_prim_,*N*_loc_) tuples for each region are represented as colours using a two-dimensional colour map.

### Specific analyses

#### Analysis of motor invariance in neural activity

Dimensionality reduction was performed as described above using a time window of 0.05–0.6 s after image onset (to avoid including data after the go cue, which, for a subset of these motor-invariance experiments, occurred 0.6–1.0 s after image onset) for fitting the PCs and for analyses that involve time-averaging (Fig. 4i–k).

To test cross-condition decoder generalization (Fig. 4k), we used a linear SVC with a one-versus-the-rest scheme for multiclass classification (LinearSVC, scikit-learn (v.1.3.0), regularization parameter C set to 0.1). We report the test accuracy, linearly rescaled so that chance level (inverse of the number of classes) and 1 were mapped to 0 and 1. Because decoders were trained and tested on different conditions (with non-overlapping trials), there was no concern of overfitting. Decoding was performed separately for, and averaged across, time bins (0.05–0.6 s relative to image onset).

#### Analysis of categorical structure in neural activity

Dimensionality reduction was performed as described above using a time window from 0.05 s to 0.9 s after image onset for fitting the PCs. For analyses involving time averaging (Fig. 5d–g), we used a window late in the planning epoch (0.6–1.0 s), when separation for A1 and A2 trials was the greatest (Fig. 5h). The primitive alignment index was computed as above using the Euclidean distance.

To assess whether primitives were associated with distinct activity patterns, we asked whether single-trial activity for each primitive was separable from the activity of every other primitive using a decoding approach (Extended Data Fig. 12). We used activity during the planning epoch (0.05–0.6 s after image onset) represented as a dataset *D* (size *K* × *NT*, where each trial is represented by a vector of length *N* × *T*; see the section ‘Representing time-varying population activity as a vector’), with its dimensionality further reduced to *K* × 50 by performing PCA and keeping the top 50 PCs. For each pair of primitives, decoding was performed using a linear SVC (LinearSVC, scikit-learn (v.1.3.0), regularization parameter *C* set to 0.1) in a cross-condition manner to assess generalizable decoding of primitives; on each iteration, we trained the model using data for one location (or size, for sessions with size variation) and tested using held-out data from all other locations (or sizes), and then averaging over analyses for different locations (or sizes). Because decoding can be biased upwards for regions with more units, to fairly compare regions that had the strongest primitive encoding (preSMA, SMA, PMv, PMd and vlPFC), we first matched their numbers of units by randomly subsampling (without replacement) to match the area with the fewest units and averaged the results from repeating this ten times. By performing this overall procedure for every pair of primitives and separately for every brain region, each time returning a single decoding accuracy score, we populated the matrices in Extended Data Fig. 12. We combined results across five sessions for each subject (S1, 2 varying in location, 3 varying in size; S2, 3 varying in location, 2 varying in size).

#### Analysis of recombination of primitive representations in the character task

We analysed primitives that were performed in both single-shape (instructed by the shape image) and character trials (the subject’s choice) in the same session. For character trials, we used only strokes scored as high-quality primitive matches. Dimensionality reduction was performed as above, using a time window of –0.8 to 0.3 s relative to the stroke onset for fitting the PCs. For analyses involving time averaging, we used a window –0.5 to –0.05 s relative to the stroke onset. Neural distance, primitive encoding and task-type encoding were computed analogous to above, using primitive and task type (instead of location or size) as the two variables.

For analyses comparing strokes from the single-shape task to the non-first stroke of the character and multishape tasks (Extended Data Fig. 14), all methods were the same as in the main analysis comparing single-shape strokes to the first stroke of character drawings except two. First, we used a shorter analysis time window of –0.35 to –0.05 s relative to the stroke onset (instead of –0.5 to –0.05 s) to match the duration of the gap between strokes (mean of around 0.3 s) in character drawings. Second, we preprocessed neural data to account for a strong effect of the reaching movement to initiate the trial (that is, reaching from the hold button towards the image; see Fig. 1c). This effect is clear in population trajectories (Extended Data Fig. 14a). To accurately assess whether primitive-encoding activity was reused across task types, it was important to correct for this effect of initial reach because it was present for single-shape trials but not for the non-first strokes of the character and multishape tasks. We performed this correction in a simple manner by subtracting, for all strokes, the across-stroke mean effect of the initial reach. This correction was performed separately for each time bin as follows. For each unit, we estimated the mean effect of initial reach by using linear least-squares regression:

$$y={\beta }_{0}+{\beta }_{f}{X}_{f}+{\beta }_{t}{X}_{t}\,+\mathop{\sum }\limits_{j=1}^{{N}_{p}}{\gamma }_{{j}}\,{Z}_{j}+{\epsilon }.$$

Here, *y* is the firing rate, *β*_0_ is the intercept term, *β*_*t*_ is the effect of task type, *X*_*f*_ is 0 or 1 depending on whether the stroke is the first stroke (always 1 for single-shape data), *X*_*t*_ is 0 or 1 depending on the task type (single-shape or character), *Z*_*j*_ is an indicator variable for the primitive class, and ${\epsilon }$ is a noise term. To correct for the effect of initial reach, we subtracted *β*_*f*_ (the mean effect of initial reach) from neural activity for all first strokes. We did not perform this correction for comparisons of single-shape strokes to the first stroke in character drawings (Fig. 6) because, there, all strokes included the initial reaching movement.

#### Analysis of primitive encoding aligned to visual fixations

We assessed the extent to which PMv activity, aligned to visual fixation events during the multishape task, preferentially encodes either the primitive associated with the visually fixated shape or the first primitive the subject is planning to draw (Extended Data Fig. 15). We first converted time-varying eye tracking data (*x* and *y* position time series) to a sequence of fixation and saccade events using Cluster Fix^100^, which, in brief, uses *k*-means clustering on distance, velocity, acceleration and angular velocity, and then assigns clusters to fixation and saccade events. Each fixation event was assigned a visually fixated primitive label, defined as the closest shape. If all shapes were further than 70 pixels (2.08 cm), then this fixation event was considered to be looking away from all shapes and was therefore excluded from further analysis. Each fixation event was also assigned a planned primitive label, based on which primitive the subject would draw first on that trial.

We assessed the extent to which fixation-aligned neural activity encoded each primitive using a decoding approach, whereby the strength of representation of a given primitive was defined as the probability score returned by a decoder trained to classify that primitive versus all other primitives. Decoders were trained using single-shape trials from the same session. Data from the planning epoch were cut into multiple short-duration snippets, each a data point used for training the decoder. Specifically, neural data were represented as a matrix *X* of size *N* × *KT*, where *N*, *K* and *T* are the number of units, trials and time bins, respectively, constructed by concatenating time bins from all trials along the second dimension (data were first binned in time using a 0.3 s window with 0.1 s slide) before constructing this data matrix. These *KT* training data points (each *N*-dimensional), each with an associated primitive label, were used to train a multilabel, logistic regression, one-versus-rest classifier (scikit-learn), which pools multiple independent classifiers, one per primitive. This resulted in a multilabel decoder that could be applied to neural data at any time point to return one probability score per primitive.

#### Analysis of encoding of kinematic variables in neural activity

To test for encoding of generic kinematics (Extended Data Fig. 16), we used a standard encoding model approach, which was based on a previous study of handwriting kinematics encoded in the human motor cortex^101^. We assessed the fraction of variance in neural activity explained by a linear mapping from moment-by-moment finger kinematics to neural activity. As in that study^101^, we used activity in the top ten dimensions found after performing PCA. PCA was performed as described above, except, instead of identifying the PCs using trial-averaged data, we did so using single-trial data to retain activity potentially related to trial-by-trial variation in kinematics. Activity at each time point was modelled as:

$${f}_{t}=E{v}_{t}+b.$$

Here *f*_*t*_ is the neural activity at time bin *t* in the top 10 neural PCs, *E* is a 10 × 2 matrix mapping kinematics to neural activity (that is, preferred directions), *v*_*t*_ is the 2 × 1 finger velocity, and *b* is an intercept term. The fraction of variance accounted for (FVAF) was computed as:

$${\rm{F}}{\rm{V}}{\rm{A}}{\rm{F}}=1-\frac{{{\rm{S}}{\rm{S}}}_{\mathrm{err}}}{{{\rm{S}}{\rm{S}}}_{\mathrm{tot}}}=1-\frac{{\sum }_{t=1}^{T}{(E{v}_{t}-{f}_{t})}^{{\rm{T}}}(E{v}_{t}-{f}_{t})}{{\sum }_{t=1}^{T}{f}_{t}^{{\rm{T}}}{f}_{t}}.$$

Here, SS_tot_ is the total variance, SS_err_ is the sum of squared errors, and *T* is the total number of time steps across all trials. Note that T (upright) represents the matrix transpose operator.

To test how strongly activity encoded kinematics in a manner generalizing across primitives, models were trained on one subset of primitives (all except one) and tested on one held-out primitive. This was performed once for each primitive, with the final FVAF taken as the mean FVAF across all train–test splits. We used sessions performing the single-shape task with location and size variation (S1, *n *= 30 primitives across 5 sessions; S2, *n* = 42 primitives across 3 sessions, which excludes 2 sessions with fewer than 6 primitives). We included data from throughout the stroke duration.

To allow for the possibility that kinematics better relate to neural activity at a non-zero lag, we performed this analysis multiple times with different time lags between neural and behavioural data, varying from –0.3 to 0.3 s (0.05 s increments). The final scalar summary averaged the results in a time window from –0.15 s to –0.05 s (with neural leading behaviour), consistent with a peak lag of around –0.1 s (neural leading) found in previous studies^102^ and in our analysis (Extended Data Fig. 16).

Note that the FVAF values we found in M1 are comparable to the average value of 0.3 in a previous study of the motor cortex in human handwriting^101^. Our finding of lower values (ranging from around 0.0 to 0.2, Extended Data Fig. 16b) is consistent with our use of trial-level instead of trial-averaged data (which introduces more variability) and of testing generalization across primitives (note that if we instead test generalization to held-out trials, this results in higher FVAF values ranging from around 0.1 to 0.3).

### Statistics and reproducibility

The findings from this study resulted from experiments that produced similar results across multiple independent repetitions: location invariance (two sessions for S1 and three for S2); categorical structure (three sessions for S1 and four for S2); and recombination in characters (nine sessions for S1 and nine for S2). Randomization was performed by having all comparisons between experimental conditions done within the same animal, with all experimental conditions (task variants × stimuli) presented randomly. No blinding in group allocation was necessary as each subject was tested in all experimental conditions. Blinding of the subject and experimenter during data collection was effectively implemented owing to the randomization and balancing of conditions across trials. All behavioural and neural analyses were performed using custom-written Python (v.3.8) code unless otherwise noted, incorporating the analysis and plotting libraries numpy (v.1.24.3), scipy (v.1.10.1), scikit-learn (v.1.3.0), pandas (v.2.0.3), seaborn (v.0.12.2), elephant (v.1.0.0) and statsmodels (v.0.14.0).

The following are detailed descriptions of statistics that did not fit in the figure legends.

For Fig. 2e, *n* = 648 (same shape, size and location: YYY), 1,296 (YYN), 1,296 (YNY), 2,592 (YNN), 5,184 (NYY), from a pool of 729 trials (81 conditions × 9 trials each). Two-sided Wilcoxon signed-rank tests comparing NYY to others: versus YNY (*W* = 0, ***P = 5.36 × 10^−15^), versus YYN (*W* = 0, ***P = 5.36 × 10^−15^), versus YNN (*W* = 0, ***P = 5.36 × 10^−15^); *n* = 81 shape, size and location conditions.

For Fig. 2j, test for sigmoidal nonlinearity: ^###^*P* = 1.91 × 10^−6^, two-sided Wilcoxon signed-rank test (*W* = 0, *n* = 20) that drawing < image (U1) and drawing > image (U2). Test for trial-by-trial switching: ***P = 1.91 × 10^−6^, two-sided Wilcoxon signed-rank test (*W* = 0, *n* = 20) that A2 > A1 (drawing).

For Fig. 2p, for remix primitive sets, three independent simulations are plotted separately. Wilcoxon signed-rank tests: results were highly significant when comparing match to one’s own primitives versus other. For S1 data, S1 versus S2 (*W* = 0, ***P = 6.75 × 10^−25^), versus S1 remix (*W* = 125, ***P = 3.43 × 10^−22^), versus S2 remix (*W* = 0, ***P = 8.75 × 10^−25^). For S2 data, S2 versus S1 (*W* = 49, ***P = 6.26 × 10^−23^), versus S2 remix (*W* = 424.5, ***P = 3.61 × 10^−16^), versus S1 remix (*W* = 0, ***P = 5.82 × 10^−25^). Results were also highly significant when comparing one’s own remixed primitives versus others. For S1 data, S1 remix versus S2 (*W* = 995, ^###^*P* = 1.41 × 10^−11^), versus S2 remix (*W* = 351, ^###^*P* = 1.90 × 10^−20^). For S2 data, S2 remix versus S1 (*W* = 731.5, ^###^*P* = 1.09 × 10^−11^), versus S1 remix (*W* = 1, ^###^*P* = 2.85 × 10^−20^). For remixed primitives, the least significant of the three simulations is shown. Sample sizes were identical for all tests (*n* = 141 characters, combining novel and practised).

### Reporting summary

Further information on research design is available in the Nature Portfolio Reporting Summary linked to this article.

## Online content

Any methods, additional references, Nature Portfolio reporting summaries, source data, extended data, supplementary information, acknowledgements, peer review information; details of author contributions and competing interests; and statements of data and code availability are available at 10.1038/s41586-026-10297-x.

## Supplementary information

Reporting Summary

Supplementary Video 1 Behaviour in the character task (example, character 1, subject 1). This depicts the trial in Fig. 2l, 4th column from the right.

Supplementary Video 2 Behaviour in the character task (example, character 1, subject 2). This depicts the trial in Fig. 2l, 4th column from the right.

Supplementary Video 3 Behaviour in the character task (example, character 2, subject 1). This depicts the trial in Fig. 2l, 7th column from the left.

Supplementary Video 4 Behaviour in the character task (example, character 2, subject 2). This depicts the trial in Fig. 2l, 7th column from the left.

Supplementary Video 5 Behaviour in the character task (example, character 3, subject 1). This depicts the trial in Fig. 2l, 3rd column from the left.

Supplementary Video 6 Behaviour in the character task (example, character 3, subject 2). This depicts the trial in Fig. 2l, 3rd column from the left.

Supplementary Video 7 Behaviour in the character task (example, character 4, subject 1). This depicts the trial in Fig. 2l, 2nd column from the left.

Supplementary Video 8 Behaviour in the character task (example, character 4, subject 2). This depicts the trial in Fig. 2l, 2nd column from the left.

Supplementary Video 9 Behaviour in the character task (example, character 5, subject 1). This depicts the trial in Fig. 2l, 1st column from the left.

Supplementary Video 10 Behaviour in the character task (example, character 5, subject 2). This depicts the trial in Fig. 2l, 1st column from the left.

Peer Review File

## Source data

Source Data Fig. 1

Source Data Fig. 2

Source Data Fig. 4

Source Data Fig. 5

Source Data Fig. 6

## Data Availability

The data used in this study are available at Figshare (https://figshare.com/s/05da05cd28329d618b94)^103^. Source data are provided with this paper.

## Code availability

The code used in this study is available at Figshare (https://figshare.com/s/05da05cd28329d618b94)^103^.
