# Supplementary material for: Neural representation of action symbols in primate frontal cortex
Source: Nature. 2026 May 20;654(8117):152–62. doi: 10.1038/s41586-026-10297-x (PMC13233313; doi:10.1038/s41586-026-10297-x)
Supplement: Supplementary file 1 — Reporting Summary [file 41586_2026_10297_MOESM1_ESM.pdf]

nature portfolio

Corresponding author(s): Lucas Y. Tian
Winrich A. Freiwald

Last updated by author(s): Jan 3, 2026

Reporting Summary

Nature Portfolio wishes to improve the reproducibility of the work that we publish. This form provides structure for consistency and transparency in reporting. For further information on Nature Portfolio policies, see our Editorial Policies and the Editorial Policy Checklist.

Statistics

For all statistical analyses, confirm that the following items are present in the figure legend, table legend, main text, or Methods section.

n/a	Confirmed	
☐	☒	The exact sample size (*n*) for each experimental group/condition, given as a discrete number and unit of measurement
☐	☒	A statement on whether measurements were taken from distinct samples or whether the same sample was measured repeatedly
☐	☒	The statistical test(s) used AND whether they are one- or two-sided<br>*Only common tests should be described solely by name; describe more complex techniques in the Methods section.*
☐	☒	A description of all covariates tested
☐	☒	A description of any assumptions or corrections, such as tests of normality and adjustment for multiple comparisons
☐	☒	A full description of the statistical parameters including central tendency (e.g. means) or other basic estimates (e.g. regression coefficient) AND variation (e.g. standard deviation) or associated estimates of uncertainty (e.g. confidence intervals)
☐	☒	For null hypothesis testing, the test statistic (e.g. *F*, *t*, *r*) with confidence intervals, effect sizes, degrees of freedom and *P* value noted<br>*Give P values as exact values whenever suitable.*
☒	☐	For Bayesian analysis, information on the choice of priors and Markov chain Monte Carlo settings
☒	☐	For hierarchical and complex designs, identification of the appropriate level for tests and full reporting of outcomes
☒	☐	Estimates of effect sizes (e.g. Cohen's *d*, Pearson's *r*), indicating how they were calculated

Our web collection on statistics for biologists contains articles on many of the points above.

Software and code

Policy information about availability of computer code

Data collection	Stimuli and behavior control used custom-written code using the Monkeylogic 2.2.45 package in MATLAB R2021a (Mathworks). Stimuli were generated in MATLAB. Neural data collection used using Synapse v98 (TDT).
Data analysis	Neural preprocessing used Kilosort 2.5 and custom-written code in Python 3.8 and MATLAB R2021a. Neural and behavioral data were analyzed using custom-written code in Python 3.8, incorporating various standard analysis and plotting libraries used in neurophysiology, including numpy (1.24.3), scipy (1.10.1), scikit-learn (1.3.0), pandas (2.0.3), seaborn (0.12.2), elephant (1.0.0), and statsmodels (0.14.0). Eye tracking data were processed into fixations and saccades using Cluster Fix. MRI/CT volume rendering used 3D Slicer (5.6.2). The code used for this study are available at https://figshare.com/s/05da05cd28329d618b94.

For manuscripts utilizing custom algorithms or software that are central to the research but not yet described in published literature, software must be made available to editors and reviewers. We strongly encourage code deposition in a community repository (e.g. GitHub). See the Nature Portfolio guidelines for submitting code & software for further information.

Data

Policy information about availability of data

All manuscripts must include a data availability statement. This statement should provide the following information, where applicable:

- Accession codes, unique identifiers, or web links for publicly available datasets
- A description of any restrictions on data availability
- For clinical datasets or third party data, please ensure that the statement adheres to our policy

The data used in this study are available at https://figshare.com/s/05da05cd28329d618b94

Research involving human participants, their data, or biological material

Policy information about studies with human participants or human data. See also policy information about sex, gender (identity/presentation), and sexual orientation and race, ethnicity and racism.

Reporting on sex and gender	n/a
Reporting on race, ethnicity, or other socially relevant groupings	n/a
Population characteristics	n/a
Recruitment	n/a
Ethics oversight	n/a

Note that full information on the approval of the study protocol must also be provided in the manuscript.

Field-specific reporting

Please select the one below that is the best fit for your research. If you are not sure, read the appropriate sections before making your selection.

☒ Life sciences ☐ Behavioural & social sciences ☐ Ecological, evolutionary & environmental sciences

For a reference copy of the document with all sections, see nature.com/documents/nr-reporting-summary-flat.pdf

Life sciences study design

All studies must disclose on these points even when the disclosure is negative.

Sample size	We used N=2 monkeys. This sample size was chosen to match the standard in previously published comparable neurophysiological behavioral studies in monkeys (e.g., Neupane et al., Nature 2024; Tafazoli et al., Nature 2025).
Data exclusions	Units with very low firing rates and high within-session firing rate drift were excluded, based on conservative criteria (described in Methods).
Replication	All analyses were independently replicated in the two monkeys (as presented in the figures). Three independent analyses of symbolic properties were performed (invariance, categorical structure, recombination), and these analyses were performed across all eight brain areas, indicating that this study's finding of action symbol representation in PMv is both robust and specific. Dimensionality reduction in population analyses was done in a cross-validated manner (splitting the data used for PCA vs. quantification).
Randomization	All comparisons between experimental conditions were performed within the same animal, with all experimental conditions (task variants x stimuli) presented randomly.
Blinding	No blinding in group allocation was necessary as each subject was tested in all experimental conditions. Blinding of the subject and experimenter during data collection was effectively implemented due to the randomization and balancing of conditions across trials.

Reporting for specific materials, systems and methods

We require information from authors about some types of materials, experimental systems and methods used in many studies. Here, indicate whether each material, system or method listed is relevant to your study. If you are not sure if a list item applies to your research, read the appropriate section before selecting a response.

Materials & experimental systems

n/a	Involved in the study
☒	☐ Antibodies
☒	☐ Eukaryotic cell lines
☒	☐ Palaeontology and archaeology
☐	☒ Animals and other organisms
☒	☐ Clinical data
☒	☐ Dual use research of concern
☒	☐ Plants

Methods

n/a	Involved in the study
☒	☐ ChIP-seq
☒	☐ Flow cytometry
☒	☐ MRI-based neuroimaging

Animals and other research organisms

Policy information about studies involving animals; ARRIVE guidelines recommended for reporting animal research, and Sex and Gender in Research

Laboratory animals	Two adult male rhesus macaque monkeys (macaca mulatta), age between 7-9 years.
Wild animals	No wild animals were used in this study.
Reporting on sex	All subjects used in this study were male. The small number of subjects (N=2) typical in primate neurophysiology studies makes it typically infeasible to isolate effects due to sex.
Field-collected samples	This study did not involve samples from the field.
Ethics oversight	All animal procedures complied with the NIH Guide for Care and Use of Laboratory Animals and were approved by the Institutional Animal Care and Use Committee of the Rockefeller University (protocol 24066-H).

Note that full information on the approval of the study protocol must also be provided in the manuscript.

Plants

Seed stocks	n/a
Novel plant genotypes	n/a
Authentication	n/a
