# Supplementary material for: Neural representation of action symbols in primate frontal cortex
Source: Nature. 2026 May 20;654(8117):152–62. doi: 10.1038/s41586-026-10297-x (PMC13233313; doi:10.1038/s41586-026-10297-x)
Supplement: Supplementary file 12 — Peer Review File [file 41586_2026_10297_MOESM12_ESM.pdf]

nature portfolio

Peer Review File

Neural representation of action symbols in primate frontal cortex

Corresponding Author: Dr Lucas Tian

Any redactions in this file are there to maintain patient confidentiality, the confidentiality of unpublished data, or to remove third-party material.

This file contains all reviewer reports in order by version, followed by all author rebuttals in order by version.

Attachments originally included by the reviewers as part of their assessment can be found at the end of this file.

Version 0:

Reviewer comments:

Referee #1

(Remarks to the Author)
Summary

This manuscript investigates behavioral and neural population signatures associated with drawing movements produced by two macaque monkeys. Using analyses of the shape and velocity of the strokes the monkeys produced, along with analyses of neural activity patterns, the authors argue for the representation of "action symbols" in PMv. The criteria defined in this work for detecting action-symbol representations are threefold: (1) the representation should be invariant to changes in the location and size of the movement; (2) the representation should display categorical structure (i.e., sharp transitions at boundaries between categories), and (3) the representation should be combinable with other putative action symbols to generate novel sequences of compound actions.

The authors find that both monkeys are highly consistent in how they produce the stroke primitives that they receive extensive practice generating, regardless of the location from which they are cued or the size at which they appear. These invariant patterns were found to be most easily decoded from PMv, rather than other other frontal areas, including SMA and dlPFC. In addition, these primitives are argued to display categorical structure, based on the non-linear/sigmoidal transition that the movements make when monkeys are cued with "morphed" shapes that interpolate between two practiced ones. PMv best predicted the neural alignment patterns between morphed and primitive shapes, including for ambiguous stimuli. Lastly, the monkeys re-used learned primitives when drawing novel composite characters and the neural signatures in PMv were highly similar for the same primitive regardless of whether it was drawn in isolation or as part of a larger composite.

Overall, this manuscript reports an impressive body of rigorous work on a set of questions that is both fundamental and timely, and almost certainly will be of deep interest to members of the neuroscience and cognitive science communities, and potentially of interest to many other segments of the Nature readership. We offer the comments below in the spirit of highlighting opportunities for clarifying key components of the argument.

General comments

Clarifying the scope of the behavioural phenomenon under investigation

A conceptual issue that remains unresolved is the question of what the relationship is between the image-cued drawing task used here and other tasks that might (or might not) recruit internal representations that display the hallmarks of "action symbols" highlighted in this work.

Would a task in which the same movements were made, but no marks were left behind be thought to recruit action symbols? In other words, how critical is it that a "drawing" task was used in this study for the purposes of investigating action symbols?

Or what about a task in which the same movements were made, but cued by something other than an image containing visual information that can be largely reproduced by hand? In other words, how critical is it that the drawing movements be visually cued by an image that contains details meant to "directly" convey the spatial and shape information needed to succeed on the drawing task —that is, a visual cue that is isomorphic to the target behavioural output? What if the cues were instead three arbitrary shapes/sounds, and the monkeys were provided with training as or more extensive in order to learn to recreate the target primitives in the intended location at the intended size? One potential response might be that while such an approach might also yield the discovery of action symbols, it would be needlessly challenging, because monkeys might not ever become proficient at the task under such settings. If so, would that suggest that there is something important about the drawing phenomenon that is under study here that depends on the nature of the visual cue? If that is the case, then does the construct of an "action symbol" also include this information about the visual cue? If not, why not?

Or what about a task in which totally different movements were made, but happened to display many of the same hallmarks — for instance, in the case of a multi-step procedure for extracting food from different containers. Suppose it were the case that three steps needed to be performed in order to extract food from one container, while two of those steps needed to be performed in order to extract food from a different container, but the third step needed was different. And suppose a monkey could be shown to perform all of those steps in slightly different postures and in different locations of a room (invariance); engage in one learned 3-step strategy or the other when presented with a novel third container that had an appearance intermediate to the first two (categorical structure); and could demonstrate proficiency in performing the "same" step even when it is embedded in two different 3-step procedure. Moreover, suppose that recordings from PMv showed similar patterns in this hypothetical setting to those reported in the manuscript. Would the internal representations of each step also be a reasonable candidate for an action symbol — because it represented a quasi-discrete unit that could form part of a multi-step plan? If that is the case, why not use such a task — which more clearly exposes the function of these actions in the context of behaviors that might be considered to be more ethologically relevant? Why instead use a drawing task that requires potentially so much more training? Perhaps the reason is that a commonsense notion of what symbols are to many people is in their manifestation as visual marks on a surface, so it seemed natural to explore the ability of monkeys to produce "written"/"drawn" symbols. But is the "written"/"drawn" nature of the behavioural traces collected in this study really central to the notion of an "action symbol" — from the perspective of the representation in PMv? If so, why? If not, why not?

Clarifying the relationship between "action symbols" and "symbolic representations" more generally.

Clarifying the above points would help the reader to better understand the relationship "action symbols" (a novel construct) and "symbolic representations" more generally. The present lack of this distinction is especially evident around L.114, where for the different behavioral phenomena cited - handwriting, drawing, object manipulation., dance etc., it is not clear that they are really using action symbols, as the authors define them, in particular. This is related to a line in the General Discussion (around L.624) where the authors seem to imply the existence of different kinds of symbol systems, all of which rely on discrete units. It would be helpful to clarify early on what makes an action symbol distinct from other symbolic representations and on the basis of those properties what kinds of phenomena may or may not be thought to use these representations.

Relationship to visual categories and visual scene representation

The primate visual system possess well studied systems for extracting "symbol-like" representations from visual inputs that seem to also meet the standards laid out for action symbols — visual object concepts are defined by their invariance over a variety of visual perturbations, display signatures of categorical structure, and visual objects can be represented in consistent ways even when embedded in different visual scene contexts.

Yet it seems to be critical to the argument in this paper that the representation in PMv is not driven by visual input, but instead encodes planned actions. Here is a passage in the Discussion to this effect: "...evidence already suggests that the circuit mechanisms underlying action symbol representation in PMv depend on local processes within PMv, as opposed to being driven entirely by image properties in a feedforward manner, or by symbol representations inherited from another region. First, as noted above, we found that PMv was not driven by image properties, but instead encoded the planned action."

What direct evidence is there for these claims in the current study? Why would it not be plausible for the structure observed in PMv to have been driven by "symbol extraction"-like mechanisms in visual cortex that operate upstream of action planning? Moreover, what is at stake / lost by assigning credit to more posterior representations more strongly associated with sensory processing for the kind of flexible "compositional generalization" observed for drawing the multi-stroke targets? Notably, there are no electrophysiological measurements conducted in these posterior areas, making it difficult to resolve whether similar neural signatures for action symbols might be decodable from such posterior representations. Arguably the actual stroke primitives themselves are exactly the overlearned stereotyped movements that the manuscript takes care to distance this study from (L683-L705) in the Discussion, and what really distinguishes this behavior from other cases is the ability to generalize to novel and more complex visual targets. What are we missing?

Re-examining support for recombination

For the neural analyses, the authors report similar representations for primitives whether they are drawn in a standalone fashion or as the first primitive in a composite character. In L.657-L.664, the authors begin by highlighting a finding about PMv that closely dovetails with findings in this paper but try to distinguish the current findings by emphasizing the categorical and recombinatorial properties observed. While category sensitivity isn't unique to PMv (see notes on visual categories above), I see the argument as "PMv is the only area that exhibits all 3 properties (invariance, categorical structure, recombination)." While the authors show similar neural signatures for primitives when drawn alone or as the first sub-part in

a character, this does not seem to be evidence for recombination. A hallmark of recombination should be that the production of primitives in a character reuse the representations from when they are produced in a standalone manner. The justifications for why subsequent primitives in characters were not compared are reasonable, but as it stands the takeaways with respect to recombination are limited.

The present argument seems to be based on the results from the behavioral analyses of stroke trajectories, which are clearly related but are not directly germane to arguments about PMv. The authors should clarify how the present neural results constitute evidence for recombination.
Additionally, for the stroke trajectory analyses, while it is interesting that the strokes used by each monkey in the character task are aligned to their own primitives, it is not clear why an emphasis is placed on action symbols being individual-specific. Since the monkeys were strongly disincentivized from deviating from strict stroke patterns during the primitive learning phase perhaps this result is a partial artifact of that. There should be more discussion of these results as it relates to the extensive training procedure for the primitives.

Clarifying the process by which the stimulus set was generated
It was not entirely clear how the specific shapes used for training were generated. Could more detail be provided concerning the rationale for arriving at this particular set of shapes, and why there are no more and no fewer? What can be said about the degree to which these shapes provide good coverage over the set of possible shapes that could have been used in this study?

Potential impact of experimenter demands on learning stroke primitives
The methods seem to imply that 'sub-strokes' were heavily penalized "we also provided feedback online by immediately aborting a trial if subjects made serious errors, including (i) if they touched a position far from any image points, and, (ii) for single-shape and multi-shape trials, if they used more than one stroke per shape in the image." This seems to imply that the monkeys were highly disincentivized from producing anything outside the primitives. What bearing does this have on the different results reported? Would we have not observed as strongly categorical primitives and re-use of the same in the character tasks under a more lenient training regime?

Clarifying the degree to which the tasks studied here constitute a novel paradigm distinct from established motor tasks.
The class of behavioral tasks reported in this paper appear to be less of a novel category of tasks than one that combines aspects of some of the earlier ones mentioned (instructed movements / working memory guided movements / minimally restrained movements). While the evaluation tasks are fairly unrestrained, the training tasks aren't and adhere more to classic 'instructed movement' tasks. These strongly learned movements might themselves strongly constrain the space of possible results that can obtain on the various behavioral generalization tasks. A clearer distinction of the current paradigm that doesn't reduce to a 'sum of previous methods' should be presented if the authors choose to emphasize the novelty of this paradigm

Bridging the theoretical/modeling paradigms laid out in the introduction/Discussion (symbolic, neural, and dynamic systems approaches).
The argumentation is that the decodability of theorized action symbols in PMv hints at the possibility of programs as being represented in neural substrates too (e.g., REPEAT(◯, 3)). But this is a bit of a leap, as there is no evidence for anything beyond simple stroke primitives in the present neural data. Either this section needs further elaboration delineating how the present results might reasonably be extended to evidence for programs or the claims should be tempered.

Specific comments

Line 1079: "95% CI" is not the correct term to use here to refer to the interval defined by the 2.5th and 97.5th percentiles of the data distribution. The CI is used for the interval defined by those thresholds over the sampling distribution of a statistic.
L67. What are 'these abilities' referring to? Compositional Generalization abilities?
L78. Why these 3 properties specifically (invariance, categorically, recombination), is this well established?
L97. No stats reported for Fig 2d./2e.
L291.Does scoring 'images' mean using Hausdorff distance? Wasn't clear here.
L605.The line about PMv showing visual invariance seemed a bit out of left field.
Fig 4 k. It is interesting that best decoding accuracy for primitives (in PMv) is higher than decoding accuracy for location in any area. Given that decoding location (4-way classification) seems like an easier task than decoding primitives, of which there are many, could the authors elaborate as to what a reader should conclude about the representation of location generally in both motor and planning areas of the brain?
L610. The first paragraph of the Discussion closes with the following two sentences: "This finding of a localized representation of action symbols in PMv indicates an important role for symbols in producing novel, goal-directed action sequences. More broadly, this finding supports the hypothesis that cognition can be understood, at the algorithmic level, in terms of internal symbolic operations…" The latter sentence is overly broad in its claims about "cognition" and does not obviously follow from the first, nor from the set of findings here.
In the present work, symbols are acquired through intensive training. Does learning any form of action symbol require such laborious learning or are there alternative ways that such action symbol representations might be acquired by primates? It would be helpful to comment on the learning of such symbols in both humans and non-human primates and where convergences and differences might lie.
Some relevant references are missing with respect to investigations of the neural computations underlying compositional

generalization. Here is one using somewhat different methods in humans, but appears to be relevant to the overarching argument: Schwartenbeck, P., Baram, A., Liu, Y., Mark, S., Muller, T., Dolan, R., ... & Behrens, T. (2023). Generative replay underlies compositional inference in the hippocampal-prefrontal circuit. Cell, 186(22), 4885-4897.
Additionally, it is a strong claim to argue in the introduction that these other phenomena rely on the hypothesized construct in the present paper before results supporting the presence of the construct have been presented.

(Remarks on code availability)

Referee #2

(Remarks to the Author)
I co-reviewed this manuscript with one of the reviewers who provided the listed reports.

(Remarks on code availability)

Referee #3

(Remarks to the Author)
This is an extremely ambitious project that will be interesting to a broad readership within neuroscience and cognitive science. The authors find that when drawing complex shapes, macaques sequentially represent discrete strokes which are idiosyncratic to each subject, and these representations are invariant to location and size of the stroke on the drawing surface, as well as the order of strokes within a shape. The authors identify neural correlates of these representations during the planning period in the ventral premotor cortex (PMv). They show extremely convincingly that the neural representations in PMv represent the primitive categorically, in a manner that is invariant to low level motor details, and in a manner that can be reused in different combinatorial plans. Three critical factors that are required for symbolic-like processing.

The article is easy to read, and builds a strong case for an exciting finding that is strongly empirically supported. The task that the authors designed, the coverage of recording sites and the established results are all outstanding features. It is particularly impressive that the authors have established a rich compositional task for primates that even allows such rich questions to be asked. The vast majority of previous electrophysiology tasks engage much simpler behaviours where it is hard to know what is overtrained, and often hard to dissociate the roles of different brain regions, particularly within the frontal lobe. This task is extremely imaginative and impressive because the space of possible problems that can be given on different trials is vast, but yet the task is amenable to formal computational description - because of its compositional nature. It is a beautiful achievement.

The strength of the manuscript is that authors focus on a very targeted part of the problem - the representation of action symbols — and provide extremely strong empirical evidence for these particular claims. They describe this evidence very thoroughly, leaving little room for doubt (hence see limited questions below). They also show that the claims are limited to PMv amongst many frontal regions. It is striking that they can identify neural representations of actions that are so consistent with regard to the shape and velocity, and yet are invariant to the location, size and “part-whole” relation to a bigger shape. The testing of ambiguous stimuli helps a lot with ensuring that the authors really are capturing motor activity and not perceptual features. The finding sits well within the context of previous PMv literature on object grasping, and extends it dramatically both computationally and functionally. It also sits naturally with PMv’s anatomical location at the junction of the dorsal and ventral streams.

If there are weaknesses of the study, they are the corollaries of the strengths. Of their 16 recording sites, the authors effectively provide an account of the neural activity only in the PMv. Because of this, they cannot give a theory of more abstract representations that are taking place during this task (how is the complex shape is represented? how the parsing of the complex shape into simpler shapes is performed? Are there representations of the sequence of actions?). This is especially unfortunate, as neither the code nor the data are available, making (i) independent testing of the representation hypothesis in their data impossible and (ii) delaying further investigation of these exceptionally interesting questions.

Overall, my view is that this article represents an extremely unusual achievement and should be published by any journal to which it is submitted. My main comment is about the choice of scoping the analysis to the author’s action symbols, but I can see this being a trade-off to provide a very meticulous understanding of these action symbols in the PMv, and on those I have no major comments (see below for minor comments).

Minor Comments
The choice of signal processing steps applied to the touchscreen data seemed overly complex. If I understand correctly, the data is acquired at 60Hz. In order to estimate velocity, the data is then (i) upsampled to 500Hz, (ii) low-pass filtered to 15Hz, (iii) "epoched" based on the upsampled data, (iv) low-pass filtered to 12.5 Hz, (v) downsampled to 25Hz, (vi) a numerical estimation of the signal's derivative is performed, and (vii) upsampled to 500Hz. Because virtually all of the results afterward rely on an accurate estimation of action symbols from the touch data, the choices here are very impactful. I think that providing an explanation for the choice of these steps will help the reader understand why, e.g., data was low-pass filtered

twice in a row, and how robust the results would be to different preprocessing steps.
The color-coded statistical significance of the author's Fig 5j, 6g and Extended data Fig 5b is missing information: What do "low" and "high" mean here? We learn in the methods that there is a mapping between a Bonferroni-corrected two-sided t-test and the colors, but we don't know what the mapping is, or what the order of magnitude of the p-values are, etc.
Looking at Figure 1, it appears that one subject prefers systematically tracing starting from the left, while the other prefers starting from the right. This makes the set of stereotypical action symbols particularly different across primates and helps subsequent analyses. But it is also a striking feature that is not discussed in the article. Were there pre-training steps to get that? Are the animals tracing with the same hand? Were there more subjects in behavior, and those were selected to acquire neural data? Or is this just a fortunate coincidence?
This dataset is rich and the authors analysis only explore part of it (as the authors acknowledge in the discussion); this is particularly visible from the fact that almost all of the recorded areas appear to be performing task-related computations, but the authors' analyses are providing insight about the PMv. However, the authors have not made the data or the code available and, from the writing of the "Data and code availability" section, they are not intending to do so. Besides the fact that reviewers cannot offer a cursory review of the code as well as the article, this means testing hypotheses on this dataset outside of the author's lab will be much harder, hindering scientific progress.
In their discussion, the authors report as surprising that their action symbol representation is confined to the PMv (and not, for example, in the PFC or the MTL). But the authors' representations are identified using not the cinematic of tracing, i.e. both location and velocity. When they conclude that they did not find symbols in the dlPFC or the preSMA, what the authors mean is that the specific signature of velocity+location arrangement that they are looking for is not present in these areas; but perhaps this is because e.g. the dlPFC contains a more abstract representation (which does not encode velocity, etc.)?
Figure 4: the authors do not plot the same PCs for PMv (PC2 and PC3) and dlPFC (PC1 and PC2). Then in Figure 5, the plot PC1 and PC2 for PMv. And then in Figure 6 they plot PC2 and PC3 again (in both PMv and preSMA). This does not impact the analyses (which rely on the first 8 PCs) but begs the question of why these PCs specifically were chosen. Similarly, different time periods are used to estimate PCs in different analyses, but no information is provided about the motivation for these choices.
Similarly, indicating which shape number corresponds to which actual shape, would be very helpful to the reader in trying to understand e.g. specific features of Fig 4j. In fact, I am not entirely sure which shapes / primitives were included, and why, in some of the figures where a number is all that's referenced.
Extended Data Fig. 5 is never referenced in the text.

Tim Behrens

(Remarks on code availability)
The data and code are not available. Clearly the authors would like to analyse t further. This is understandable given the vast scope of the project and the limited focus of the current manuscript within this scope. However, it is clearly also disappointing (and perhaps against NIH policy?). I recommend the editors and authors negotiate a time period within which it will be made available.

Referee #4

(Remarks to the Author)
Overall evaluation:
In this study, the authors presented both behavioral and electrophysiological results from macaque monkeys that learned to trace/draw different stroke patterns on a touchscreen. By training monkeys to reproduce in one stroke a wide variety of visually presented simple geometric patterns first (horizontal/vertical lines, half/full circles, zigzags, etc.), the authors attempted to foster symbolic representations for these "primitives" of finger movements. To verify that such representations truly reflect action symbols, the authors performed analyses evaluating three requisite features of symbols: motor invariance across locations/stimuli magnitudes, (discrete) categorization of learned stroke patterns, and applied recombination (of primitives) when reproducing complex shapes.
The authors provided both neural and behavioral data suggesting motor invariance and sigmoidal/category-like responses to the ambiguous morphing of two "primitives". However, the former has already been established by previous studies, and the latter has little bearing on whether or how such "primitives" were actually utilized by the animals. Regarding recombination, this study offered tangential evidence in the form of "task invariance" such that a shape could be drawn similarly when drawn by itself or as part of a complex. Unfortunately, current data could not address either the neural mechanism of the stroke pattern "decomposition" required to complete a novel drawing task with primitives or the self-generated positional/ordinal relationships among the stroke primitives.
This work is truly ambitious, introducing a creative paradigm with great potential. The great effort it took to survey various cortical areas with closely placed microelectrode arrays should also be acknowledged. With admirable courage, the authors addressed a very important question of the field, that of symbolic representation which, as the authors rightfully surmised, is key to our understanding of problem-solving using learned knowledge, and of intelligence in general. That said, as things stand, current evidence remains insufficient to call what the authors observed "neural representations of action symbols".

Major issues:
1. My first concern is whether/to what extent the macaques acquired a system of symbols that could be utilized to accomplish various tasks, judging by the animals' behavioral performance. The authors trained the subjects for many generalization tasks to show that the symbol system was well established. However, some of the evidence seemed ambiguous:
(a) Macaques were trained to learn > 15 different stroke patterns. How are these primitives independent from each other (as

would be expected for “discrete units” mentioned in the abstract)? From Fig. 2o, we could see that some primitives were used more frequently than others by subject 1, while according to the subsection “Task types/Character tasks” in Methods, a well-trained subject could use all the primitives with similar frequencies since the shapes in characters were sampled randomly. Another point against independence is that we could see many supposed “primitives” being decomposed into other primitives in Fig. 2l. Does this imply that the macaques only learned some of the primitives? If so, the authors probably could try to specify a minimum symbol set.
(b) Moreover, similarity/distance metrics, while useful in establishing behavioral/neural fidelity, could not directly tell us whether stroke patterns were truly acquired by the animal as independent action symbols (also note that there are certain off-diagonal regions in Fig. 2d with distance values as low as part of the diagonal).
(c) Following a), there were some primitives that were rarely used in recombination tasks. For example, a circle is very scarcely drawn as a single circle primitive for subject 1 (as shown in Fig. 2l, 4 out of 5 circles were drawn as a combination of other primitives, and also in Fig. 2o, the matches for the circle are very few and of relatively low quality). However, in Fig. 2g, the circle was shown as an example for categorical structure. It is confusing whether the circle primitive was actually learned by the macaques. The authors could include behaviors across all the different tasks to justify the acquirement of each primitive since incongruency will undermine their conclusion.
(d) Following c), the sigmoidal “category boundary” results regarding between-shape morph sets are encouraging, but they are not necessarily proof of the usage of independent action symbols; training-induced motor output bias towards learned movements (being more efficient to perform and to thereby obtain a categorical-score-dependent reward, thus constraining degree of freedom) might also explain the data. What behavioral/neural evidence is (or could be) there to support the notion that these animals were using symbols to perform each task, and not simply getting more skillful at generating motor responses de novo?
(e) In addition, the equivalent to Fig. 2o for subject 2 appears absent.

2. My second concern is, supposing the macaques acquired the symbol system, whether the recorded neurons support such symbol representations. The electrophysiological recordings covered multiple cortical regions, among which only PMv showed potential symbolic encoding. However, as the authors already stated in the Discussion, PMv encodes invariant action representations (Taghizadeh et al., Nature Communication, 2024; Harpaz et al., Neuron, 2014). The results could be explained as an egocentric encoding of action sequences in PMv. The authors emphasized that the differences between action symbols and invariant action representations are categorical structure and recombination. However, since the macaques were eventually led to perform action sequences in these tasks, it’s also possible that PMv was just encoding the actions as the output of a symbol system, rather than the symbol system per se. In other words, the authors didn’t decorrelate the symbol system with the action output it produces.

3. My third concern is with insufficient analysis of symbol recombination. As suggested by the authors’ previous paper (Science, 2015), recombination involves the structured organization of discrete symbols through specific relational rules (e.g., directional or sequential relationships). The current manuscript lacks exploration of such rules at both behavioral and neuronal levels, weakening the authors’ claims for evidence of recombination.
At the behavioral level, with more than a dozen “primitives”, the learned stroke patterns are perhaps too exhaustive for alternative draw paths to appear; that is, whatever motion on the touchscreen a monkey makes, it might become significantly similar enough to one of the “primitives” regardless of the monkeys’ intention. The current task paradigm therefore may not be able to offer a suitable control against possible alternative hypotheses in Fig. 2k and beyond. Perhaps seeing what monkeys would do if trained to reproduce complex traces without previous training on “primitives” could help?
At the neuronal level, the manuscript does not investigate how relationships between primitives (e.g., their order, spatial arrangement, or hierarchy) are neurally encoded during novel pattern reproduction. For instance, if recombination involves combining primitives via relational rules (e.g., “up-left-down”), neural activity may reflect the primitives and the transitions/rules linking them.
In summary, recombination requires evidence of structured relationships between primitives, not just their isolated usage. The current analyses do not adequately address this, and additional experiments or re-analysis focusing on relational dynamics would be needed to substantiate the central hypothesis.

Minor issues:
1. Fig. 2o could perhaps be normalized into a high/low-quality match ratio to accommodate different numbers of total matches per category.
2. What symbols are used in Fig. 2d and Fig. 4, exactly? Showing the stroke patterns would help.
3. There seems to be some disassociation between behavioral and neural data analyses; for instance, are the “primitives” best learned by subject 1 (as seen in, say, Fig. 2o) also the ones best represented by neural activity? Are neural responses to the same “primitive” stimulus similar or distinct across monkeys like the behavioral outputs in Fig. 1e?
4. Notably, the neural data on recombination came from -0.6 to 0.2s around the first stroke start (Fig. 6b) instead of the planning period around image onset used in previous analyses. The peri-stroke-onset time window might provide data that could be too complex to sort through (mixture of motor signals from hand rising to touch screen, motor planning, “Go” cue comprehension, etc.).

(Remarks on code availability)

Version 1:

Reviewer comments:

Referee #1

(Remarks to the Author)
We thank the authors for their detailed responses to our comments and to the substantial edits, both in terms of clarifications in the manuscript text and follow-up analyses that help bring greater clarity to the key questions at hand.

The edits to the Introduction and Discussion sections in particular help motivate, clarify and address several points raised in the initial review. The additional references added to the Discussion section also help bridge the current work to the existing literature (e.g., relating to hippocampal replay).

The new experiments assessing the (in)ability to decode visual features of the cueing stimulus, indexed by which part of the stimulus the subject is attending to during planning, relative to motor plans helps support the argument that pMV is primarily involved in planning action sequences and is not directly driven by the visual input being attended to. This helps distinguish the extent to which pMV engages in processing visual information vs. motor planning and addresses comments raised in the initial review. The follow-up analyses investigating the state-space trajectories of different primitives, both when drawn in isolation or as a part of composite characters as the first or second+ stroke, help highlight that there does appear to be reuse of same primitive-specific neural code when primitives are combined, addressing our initial comments regarding the lack of neural evidence for the same.

Furthermore, we appreciate the extensive comments provided in response to the thought experiments we posited in our original review to more deeply understand the notion of "action symbols" and "compositional generalization" that are so central to this work.

As we understand it based on the response letter, in the authors' view, it is not critical that the action symbols result in visual marks, so long as they are internally constructed and goal-directed. However, it does seem important for an action symbol to be cued in such a manner that "surface-level" stimulus information is not sufficient to determine the behavioral output, and that instead an "internal constructive process" is needed. Indeed, these are the reasons given for why using arbitrary sequences of shapes/sounds (such as a red/green cue) to cue the production of actions—even if these actions possess all the hallmarks of action symbols—would not be sufficient to elicit genuine action symbols. Based on our understanding of lines 777-783 in the Discussion, ambiguity in the mapping between the sensory input and the motor output is argued to be critical for engaging true action symbols and for investigating true compositional generalization.

However, there remain importantly puzzling aspects of these criteria that, to our minds, remain unresolved.

If it is the case that such ambiguity in how to parse the sensory input is a critical feature of an action symbol / compositional generalization, that would suggest that a key computational challenge is the "input parsing problem" and not only the action representation. The "input parsing problem" (so to speak) in the case of these image-cued drawing tasks is the problem of visually decomposing the target character. Without the challenge posed by the "input parsing problem," it would seem to be the case that the "GREEN RED" cue example (given in author response) ought to qualify, especially if that specific sequence of cues had never been seen or acted upon by the animal before. So either the "GREEN RED" cue example should be admissible, or it should be acknowledged that the construct under investigation is one that relies crucially on the ability to parse ambiguous (visual) inputs, and not so much the ability to compose previously learned action primitives in novel ways (if it were, then the "GREEN RED" example ought to be admissible). But if "the ability to compose previously learned action primitives in novel ways" is not the core phenomenon under investigation, then it is much less clear why "compositional generalization" is an appropriate term to apply to it.

Supposing a task that poses a challenging "input parsing problem" is actually an essential ingredient for studying compositional generalization (which I take the authors' response to have insisted upon, even if we remain unconvinced), then something else that remains unclear to us is what role Pmv is thought to play in the "input parsing" operations themselves and not just representation of the cued actions,which are visual operations in the context of the Character task. Clarification on this front is particularly important in light of the new experimental results in Extended Data 14., showing limited decodability of visual gaze patterns from Pmv. If Pmv is thought to play a crucial role, what direct evidence is there that these input parsing operations are performed in that region? If Pmv is not thought to play a crucial role, then what does that imply about the relationship between Pmv and action symbols / compositional generalization?

We were puzzled by the response to our thought experiment concerning the sequences of actions needed to open various food containers. While we were reassured that such scenarios might also hypothetically involve action symbols and support genuine compositional generalization, we did not understand why this setting was inherently less suitable for studying these phenomena than a drawing task. And we thought it possible that the "food container" example we gave might have been misunderstood. In this scenario, really meant to be a stand-in for a "physical problem-solving" or "tool-use" variant of the task posing the very same set of computational challenges, we do not really anticipate that the resulting movements produced by a participant would actually be "discrete" because they would be interacting with real physical containers. And certainly the way a food jar looks does not directly "instruct" a participant when it comes to how to open it. Suppose there was a tab that needed to be pulled, or a flip top, or a screw top, or a cloth covering. Food jars with these different lids look even less like the actions needed to open them than the letter "G" looks like the action needed to reproduce that letter by hand (cf. observation about isomorphism between visual input & motor output in the Character task), even if the kinds of actions needed to interact with each of the possible lids (e.g., screw top, flip top, cloth covering) are themselves reasonable candidates for representation as "action symbols." Moreover, there are many such physical mechanisms that can be devised and learned, much as there are many characters that can be devised and drawn/written. Though it might simply be more expedient given

the state of digital tablet technologies to use a drawing task rather than devising many physical puzzles. Is that right?

On a separate note, while the new 'remixed' stroke analyses (Figure 2p.) are fascinating and do help contribute to the narrative that primitives are subject-specific and re-used, it was unclear how the strokes were remixed in the main results text and figure. While the remixing algorithm is described in both the methods and extended data, it would be helpful to at least note that primitives were remixed within-subjects earlier in the text, as this helps contextualize the results.

Additionally, the current takeaway for this result is presented on L.353, "Combined, these control analyses show that character strokes specifically resemble one's own primitives (first analysis) in their idiosyncratic stroke trajectories (second analysis)."
What are the consequences of a subject's primitives (action symbols) being so highly specified that they only match a subject's own (the highly trained) idiosyncrasies? Greater discussion of this finding is warranted.

The authors have clarified their stance on the importance of constraints on naturalistic skill learning (handwriting, musicianship, athletics, etc.) and have highlighted that even if primitives are learned, the core contribution in the present work is evidence of how these primitives can be generalized, which we agree is not a trivial feat. Nevertheless, it would be helpful to comment on the importance of how the action primitives were induced in the present study and how that might relate to how action symbols might emerge in naturalistic behavior. Would the decodability of signals from pMV have been noisier if the learning of primitives were less strictly enforced (noting that the authors did allow for some flexibility in how primitives were learned – e.g., results from Figure 2p.)? In a non-drawing task, where there is even looser isomorphism between cued stimuli and desired actions, would more training/learning be needed to arrive at action symbols?

Thank you again for your thoughtful response and we look forward to hearing what you think after having reviewed the above comments.

(Remarks on code availability)

Referee #2

(Remarks to the Author)
I co-reviewed this manuscript with one of the reviewers who provided the listed reports.

(Remarks on code availability)

Referee #3

(Remarks to the Author)
The strengths and weaknesses of the article are not really changed from the last version, but the article has nevertheless improved. The article should now be published (subject to one very minor change below that does not require re-review), and the authors commended for their attention to the comments and commitment to open science.

Notable changes:

• They now provide source code and data. My lab wrote small scripts of our own to plot neural activity and behavioural activity, which wasn't too hard, and as far as we tested, all of their code works and produces the paper's figures.
• They added reasonable analyses related to (i) where the animal is looking, to show that the visual content is what drives the PMv activity, and (ii) encoding analysis from velocity straight to neural data, which shows that while it explains some of the M1 activity, it doesn't explain the PMv activity.
• They also provide useful analysis of neural activity of the middle- and late- strokes in multi-shapes trials, which require doing some correction for the fact that the first "shape" starts with a massive motor movement from the fixating "poke" to the shape onset.
• They provide rather unreadable new figure 13 (Minor comment: In my view, these scales are harmful for the message they are trying to convey; binarise, or give us real scales, the current version makes it impossible to know what is significant and what isn't)
• The discussion is more structured and now includes a section about ventral and dorsal pathways and the location of PMv in all of this, and another making it clear what they mean by their finding being an abstract "action symbol".

Importantly, one of the major concerns that I had reading the other reviewers' comments has been cleared: they are _not_ penalised if they don't use sub-shapes (which, indeed, would have made the claim much weaker). This comes from a slightly confusing terminology between single shape, multiple shapes (several on screen, but disconnected; there it is penalised to use a single stroke since they are disconnected) and character (complex shape). Maybe they could find a better name or make the terminology clearer in fig. 1.

The biggest drawback of the paper remains true: As pointed out by all reviewers this is not a paper about compositionally or how things combine or rules, etc.. However, they are careful not to claim that besides one opening in the end of the discussion. The data they do present are nevertheless remarkable.

(Remarks on code availability)
The code is available and can be used by a skilled postdoc :)

Referee #4

(Remarks to the Author)
We appreciate that the authors' point-by-point response is drafted with impressive thoroughness and outlines a greatly improved manuscript. There are still a few issues where my opinion diverges from that of the authors.

I agree with the authors that, for the purpose of demonstrating that some primitives were learned and reused to draw complex figures, what's already in the manuscript should suffice. However, the comparison to language and other forms of acquired compression is not without question. Unlike these cases where the inherent structure of environmental inputs could lead to biased distributions, in the case of this study's single strokes, the training was presumably conducted without bias among all stroke types. If this is not so, then perhaps the main text should make it clear.
Another of my concerns was with whether one could indeed call what the animals learned a "basis set", given the tendency for some primitives to be rarely used and/or decomposed when drawn (which would reduce trace similarity and therefore reward). Wouldn't the monkeys be encouraged to use the most efficient primitive with the goal of reproduction in mind? I understand that the usage of primitives is not necessarily efficient in every case, but still it challenges parsimony in explaining behavior. That being said, within the authors' stated scope, it may already be sufficient to demonstrate that certain reusable primitives were sufficiently independent of each other. It might be still better to show directly that even rarely used and/or often "decomposed" primitives were nonetheless reliably reused when drawing complex figures.
Moreover, I would like to discuss the integrity of the putative action symbols. In handwriting, it is fine to write "d" as an "o" and an "l" connected together. Even if one does so, the semantic meaning of "d" would not be decomposed into "o" and "l", which I would call the integrity of meaning for a symbol. However, the semantic meaning of an action symbol is perfectly aligned with the process of producing it. In other words, the "meaning" of an action symbol is defined by how it is produced. This is part of what's causing my confusion when the subject parses a known primitive into several other primitives; does it also decompose the meaning of a primitive, or does it just decompose the production? If the former, when the subject draws a circle as a squiggle + line, there should be a compound representation of squiggle + line in neural activity. If it is the latter case however, the neural activity should represent a circle. I think it is worth diving into those cases more carefully.

While I concur with the authors that a more comprehensive investigation of relational rules and their underlying neural mechanisms falls beyond the scope of this study and should be deferred to future work, I remain concerned that the current PMv data pertaining to "recombination" are somewhat limited in their interpretability.
Specifically, to robustly demonstrate recombination, it would seem necessary to present neural data that involves interaction between representations of at least two distinct strokes (e.g., by examining the bivalent parsing of complex shapes). Although Extended Data Figure 14 partially addresses this, the evidence remains insufficient. Moreover, the consistency of stroke representations across different ranks in complex symbol drawing, while informative, does not fully substantiate the claim of recombination. If recombination is occurring at the output stage of motor processing, one might expect contextual modulation to differentially affect the first and second strokes, thereby reflecting their distinct roles in the sequence. Currently, the PMv data appear to reflect the elementary components/"ingredients" (cf. the discrete stroke class z_ij in Lake et al., "Human-level concept learning through probabilistic program induction") of recombination, rather than the dynamic process itself. It may be worthwhile to re-examine the dynamics of potential contextual modulation within the related time window.
Additionally, I suggest moving Extended Data Figure 14 to the main text, as Figure 6 (which focuses on recombination) is currently under-supported and would benefit from more direct evidence.
Furthermore, the authors' proposed "recombination" is framed so far mainly as "reuse." I agree that simultaneously addressing action symbol representation and syntactic structure may be overly ambitious for a single paper, especially since the neural representation of action symbols itself has not yet been firmly established. Thus, focusing solely on action symbols is justifiable. However, I find that the novelty of the study is limited if it only demonstrates neural representation of action symbols with motor invariance, as this phenomenon has previously been observed in other motor areas. More abstract representation of movement—even across varying combinatorial contexts—is already a known capability of motor cortex and does not, in itself, constitute evidence of recombination. In this context, I recall the work by Zimnik and Churchland ("Independent generation of sequence elements by motor cortex"), which appears to preempt in PMd much of the conclusions presented here—especially if one considers a double reach task as also based on the elementary "action symbol", i.e. the reach movements, which in their work are also invariantly represented and can be recombined to form a variety of complex movements. While it is encouraging that only PMv shows all of the properties for action symbols in the current manuscript, the novelty is to some extent compromised, when the take-home message can be summarized as "reuse of invariantly represented movements", with no further analysis for the mechanism of recombination/relation, nor analysis of how exactly the primitives are represented in PMv. Perhaps the dynamic features in the planning period could be explored further, rather than simply having them described as distinct trajectories.

(Remarks on code availability)

Version 2:

Reviewer comments:

Referee #1

(Remarks to the Author)
We thank the authors for their response. However, we continue to believe that there is still a need to resolve an open question regarding the emphasis placed on "ambiguity" in the stimuli used to cue action symbol representations. This ambiguity is precisely what poses the "input parsing problem" raised in earlier rounds of review, concerning the centrality of visual processing in thinking about action symbol representations.We suggest two potential ways to address our concerns.

If the authors believe that the problem of visually decomposing ambiguous stimuli such as the glyphs used in the present studies is not central for supporting action symbol representations, but is mostly useful from a methodological perspective, then we think it would be appropriate to soften claims about the novelty and centrality of their experimental paradigm in investigating action symbol representations. There needs to be strong justifications for why simpler task paradigms (refer to sound/light cueing examples from earlier correspondences) would not test for the existence of action symbols and the need for the drawing-based paradigm.

If, on the other hand, the authors believe that what is truly key about the drawing task is the fact that the glyphs are ambiguous with respect to what actions must be performed (i.e., impose “minimal instruction from the environment”) and that many potential ways of drawing the glyph can be conceived (each recruiting different action symbols), then the “input parsing problem” feels deeply central to the phenomena under study. That is, the action(s) used to produce the drawing are reliant on how the agent discretizes a static image of a glyph into segments, each of which must be reproduced using discrete actions. The authors should emphasize the importance of the problem of visually parsing these shapes as a core prerequisite for action symbol representations. To be clear, we are not saying that PMv is the site of the parsing (the results of the experiments reported in Extended Data Fig. 15 help dissociate action plans from perception), but that a key property of learned action symbols is that they require parsing otherwise ambiguous sensory inputs. These operations might ostensibly be related to operations in sensory processing regions upstream of PMv.

We would also gently suggest some additional close editing passes over the manuscript to ensure that the argument narrative flows as intended.

(Remarks on code availability)

Referee #2

(Remarks to the Author)
I co-reviewed this manuscript with one of the reviewers who provided the listed reports.

(Remarks on code availability)

Referee #4

(Remarks to the Author)
All my comments have been addressed. I would like to congratulate the authors on a very nice piece of work.

(Remarks on code availability)

Version 3:

Reviewer comments:

Referee #1

(Remarks to the Author)
We thank the authors for their detailed response and for engaging with our questions throughout multiple rounds of review. We believe that the passages in the manuscript that emphasize the distinctive (but not unique) value of using a cued drawing task, where the cue is intended to be parsed in a way consistent with prior experience, have been improved. Ultimately, we believe that this is important work and we are looking forward to seeing these findings presented in the best possible way in its final published form.

(Remarks on code availability)

Referee #2

(Remarks to the Author)
I co-reviewed this manuscript with one of the reviewers who provided the listed reports.

(Remarks on code availability)

Open Access This Peer Review File is licensed under a Creative Commons Attribution 4.0 International License, which permits use, sharing, adaptation, distribution and reproduction in any medium or format, as long as you give appropriate credit to the original author(s) and the source, provide a link to the Creative Commons license, and indicate if changes were made.
In cases where reviewers are anonymous, credit should be given to 'Anonymous Referee' and the source.
The images or other third party material in this Peer Review File are included in the article's Creative Commons license, unless indicated otherwise in a credit line to the material. If material is not included in the article's Creative Commons license and your intended use is not permitted by statutory regulation or exceeds the permitted use, you will need to obtain permission directly from the copyright holder.
To view a copy of this license, visit https://creativecommons.org/licenses/by/4.0/

Referees' comments:

Referee #1 (Remarks to the Author):

## Summary

This manuscript investigates behavioral and neural population signatures associated with drawing movements produced by two macaque monkeys. Using analyses of the shape and velocity of the strokes the monkeys produced, along with analyses of neural activity patterns, the authors argue for the representation of "action symbols" in PMv. The criteria defined in this work for detecting action-symbol representations are threefold: (1) the representation should be invariant to changes in the location and size of the movement; (2) the representation should display categorical structure (i.e., sharp transitions at boundaries between categories), and (3) the representation should be combinable with other putative action symbols to generate novel sequences of compound actions.

The authors find that both monkeys are highly consistent in how they produce the stroke primitives that they receive extensive practice generating, regardless of the location from which they are cued or the size at which they appear. These invariant patterns were found to be most easily decoded from PMv, rather than other other frontal areas, including SMA and dlPFC. In addition, these primitives are argued to display categorical structure, based on the non-linear/sigmoidal transition that the movements make when monkeys are cued with "morphed" shapes that interpolate between two practiced ones. PMv best predicted the neural alignment patterns between morphed and primitive shapes, including for ambiguous stimuli. Lastly, the monkeys re-used learned primitives when drawing novel composite characters and the neural signatures in PMv were highly similar for the same primitive regardless of whether it was drawn in isolation or as part of a larger composite.

Overall, this manuscript reports an impressive body of rigorous work on a set of questions that is both fundamental and timely, and almost certainly will be of deep interest to members of the neuroscience and cognitive science communities, and potentially of interest to many other segments of the Nature readership. We offer the comments below in the spirit of highlighting opportunities for clarifying key components of the argument.

We thank the reviewer for this clear summary and positive comments.

## General comments

### Clarifying the scope of the behavioural phenomenon under investigation

A conceptual issue that remains unresolved is the question of what the relationship is between the image-cued drawing task used here and other tasks that might (or might not) recruit internal representations that display the hallmarks of "action symbols" highlighted in this work.

Would a task in which the same movements were made, but no marks were left behind be thought to recruit action symbols? In other words, how critical is it that a "drawing" task was used in this study for the purposes of investigating action symbols?

We do not think it is critical to the idea of action symbols for visual marks to be produced; instead, we think action symbols are likely to be involved for movements that are purposeful (i.e., internally

constructed and directed towards achieving a goal) and represented in terms of concepts that abstract away from motor parameters (e.g., "draw three circles left to right"). It is important to emphasize that whether any given behavior involves action symbols is an empirical question. A major contribution of our study is an empirical framework for asking this question at both the behavioral and neural levels. Importantly, our use of a drawing task was motivated by certain properties that make it ideally suited for studying action symbols in macaques. See our elaboration of both of these points below.

- We have updated the manuscript to further emphasize these points, which we describe in our responses to the next two comments below.

Or what about a task in which the same movements were made, but cued by something other than an image containing visual information that can be largely reproduced by hand? In other words, how critical is it that the drawing movements be visually cued by an image that contains details meant to "directly" convey the spatial and shape information needed to succeed on the drawing task —that is, a visual cue that is isomorphic to the target behavioural output? What if the cues were instead three arbitrary shapes/sounds, and the monkeys were provided with training as or more extensive in order to learn to recreate the target primitives in the intended location at the intended size? One potential response might be that while such an approach might also yield the discovery of action symbols, it would be needlessly challenging, because monkeys might not ever become proficient at the task under such settings. If so, would that suggest that there is something important about the drawing phenomenon that is under study here that depends on the nature of the visual cue? If that is the case, then does the construct of an "action symbol" also include this information about the visual cue? If not, why not?

This is an important comment, which reveals to us the need to clarify how the nature of the visual cue relates to the concept of action symbols.

First, we emphasize that action symbols do not require movements being directly cued by visual (or other sensory) cues that are "isomorphic to the target behavioural output". In fact, we believe that it is rather the opposite—action symbols are critical when the surface-level stimulus information (and/or memorized responses to specific stimuli) are *not* sufficient to instruct the details of behavioral output, instead requiring an internal constructive process. This was the case in our task paradigm. In general, the image showed only the final target, and not the movements needed to produce that target. These movements had to be inferred by the monkey, especially for novel and ambiguous images. This is evident in the experiment on categorical structure, where the same image was performed differently across trials (**Fig. 2f-j** and **Fig. 5**), and in novel character tasks, where subjects did not directly draw each component shape in the image, but instead interpreted the overall ambiguous image in terms of primitives (**Fig. 2k-p** and **Fig. 6**). Moreover, all task paradigms for motor invariance, categorical structure, and recombination tested the subjects on novel images (**Fig. 2**). In short, an action symbol is an internal representation that supports compositional generalization. They can sometimes be directly tied one-to-one to particular visual cues, but this is not necessary.

To be clear, consider, as a contrast, previously published macaque tasks where a visual cue is indeed isomorphic to the required behavioral details. Examples include tracing tasks where the cue is either a moving cursor that the finger must follow (Schwartz Science 1994) or one out of a very small set (~3-5) of highly-practiced images (Averbeck ExpBrRes 2003). As compositional generalization was not tested in these and related paradigms, it is not clear whether subjects used action symbols. They may not

have been incentivized to learn action symbols because they could solve the task by directly following instructive cues.

The above points have been incorporated into a new paragraph in the Discussion, starting with "Our study introduces a paradigm to study compositional generalization" (lines 776 to 789).

We also note that there is another kind of visual cue, one that involves an arbitrary, learned mapping to movement, such as a green cue that means *draw circle*, and a red cue that means *draw line*. This is the kind of cue in the reviewer's suggested task ("three arbitrary shapes/sounds"). In such a task, one might also expect action symbols. While we agree it would be interesting to study, we do not think this task is necessary in order to draw our conclusions regarding the neural substrates of action symbols. In fact, we think our task has an advantage of being more naturally suited to testing generalization (a key feature in studying symbols), because we can readily generate novel, complex and systematically-varying images that are interpretable as compositions of action symbols and yet ambiguous to exactly which symbols to compose and how. In contrast, in a task where actions are cued directly, one would have to incorporate additional layers of rules to test compositional generalization. Simply presenting two basic cues in order (e.g., GREEN RED) would not be sufficient, as the subject could simply respond to each cue one by one. A suitable approach could be to train higher-order rules, such as "blue cue means repeat twice", so that a test problem could be a blue cue next to a green cue, which would mean *draw circle twice*. This goes beyond the scope of the immediate question of action symbols, but would be an interesting paradigm for future studies.

To refute the reviewer's suggestion that an action symbol may be directly tied to its associated visual features, we have incorporated additional data and analyses to show that PMv exhibits a form of abstraction in which it is not directly driven by visually-fixated shape cues:

- We performed an additional set of experiments using images with multiple shapes present in the image during planning (with the subject choosing which of these to draw first), and tracked the subject's gaze as he visually scanned each shape. We found that PMv activity was not affected by what shape was visually fixated on, but instead strongly encoded the planned first primitive. This result has been added as **Extended Data Fig. 15.**
- In the new Results section ("PMV encodes primitives in a manner dissociated from both the shape in view and from motor kinematics", lines 638 to 666), this result has been combined with an additional analysis showing that PMv activity is not related to generic motor kinematics. The point of this new Results section is that PMv activity is abstract (not directly linked to either visual or motor parameters).
    - In the analysis of motor kinematics, we found that PMv has essentially no generalizable encoding of kinematics, while, in contrast, M1 does show significant encoding of kinematics. This result has been added as **Extended Data Fig. 16.**
- These two results contribute to a new paragraph in the Discussion, which summarizes our study's evidence that PMv's action symbol representation is not directly related to visual or motor parameters: The paragraph starts with "Several lines of evidence indicate that this action symbol representation in PMv is abstract…" (lines 722 to 735).

Or what about a task in which totally different movements were made, but happened to display many of the same hallmarks — for instance, in the case of a multi-step procedure for extracting food from different containers. Suppose it were the case that three steps needed to be performed in order to extract food from one container, while two of those steps needed to be performed in order to extract

food from a different container, but the third step needed was different. And suppose a monkey could be shown to perform all of those steps in slightly different postures and in different locations of a room (invariance); engage in one learned 3-step strategy or the other when presented with a novel third container that had an appearance intermediate to the first two (categorical structure); and could demonstrate proficiency in performing the "same" step even when it is embedded in two different 3-step procedure. Moreover, suppose that recordings from PMv showed similar patterns in this hypothetical setting to those reported in the manuscript. Would the internal representations of each step also be a reasonable candidate for an action symbol — because it represented a quasi-discrete unit that could form part of a multi-step plan? If that is the case, why not use such a task — which more clearly exposes the function of these actions in the context of behaviors that might be considered to be more ethologically relevant? Why instead use a drawing task that requires potentially so much more training? Perhaps the reason is that a commonsense notion of what symbols are to many people is in their manifestation as visual marks on a surface, so it seemed natural to explore the ability of monkeys to produce "written"/"drawn" symbols. But is the "written"/"drawn" nature of the behavioural traces collected in this study really central to the notion of an "action symbol" — from the perspective of the representation in PMv? If so, why? If not, why not?

We would interpret such findings, assuming the subjects also generalize compositionally, as indeed involving action symbols, because the results satisfy all the criteria we proposed for action symbols. This would be consistent with our conceptualization of action symbols as generally relevant for motor behaviors that involve structured internal representations of discrete action sequences, and which can exhibit generalization to novel situations.

The reason we used a drawing task is not because there is something important about the “written/drawn” nature of the behavior to the notion of an action symbol. Instead, it is because this task has many useful qualities for testing for compositional generalization. A first reason is that it conveys the target image in a manner not directly instructing the detailed motor trajectory or what strokes to use. This creates a situation in which solutions—especially when generalizing to novel, ambiguous images—reflects the subject's understanding of the underlying compositional generative structure of these images (as combinations of action symbols). A different task where the stimuli or instructive cues themselves are clearly discrete (e.g., three containers that must be opened in sequence) makes it difficult to test whether symbolic structure reflects an internal representation, or is instead simply inherited from the stimuli. A second reason is that drawing offers flexibility in generating novel stimuli for testing invariance, categorical structure, and recombination. We can present images with continuously varying parameters for testing invariance and discrete categorical structure. It also is well-suited for testing recombination, because there is a large space of target images that are novel and ambiguous.

We have added these explanations of the advantages of drawing as a task paradigm in the Discussion, in the paragraph starting:

- “Our study introduces a paradigm to study compositional generalization.” (lines 776 to 789).

The reviewer’s comment suggests that our paper’s criteria for symbolic representations could form a valuable framework for testing for symbolic representations in other and even vastly different behavioral paradigms. We agree that this is an important implication of our study. We previously conveyed the generality of action symbols, including in the Introduction, where we reference a variety of kinds of motor behaviors which may involve action symbols (e.g., writing, drawing, object manipulation, tool use, dance), and in our definition of action symbols (“internal, recombinable representations of discrete units

of motor behavior”). We have now revised the main text to emphasize the reviewer’s suggestion that our study’s framework can be applied to other motor behaviors.

- Introduction:
    - “and may be related to the movement motifs (or “syllables”) that have been identified in various animal behaviors” (lines 107 to 108).
- Discussion:
    - “Our findings raise the question of whether PMv’s involvement in diverse motor behaviors—including speech—may be explained by the single principle that PMv encodes action symbols, a question which may be addressed by testing for symbolic properties, abstraction, and involvement in compositional generalization” (lines 749 to 754).
    - “A related line of research has identified reused motifs (or “syllables”) in minimally-restrained behaviors, including in birdsong, and spontaneous rodent behavior. For both motor primitives and syllables, it remains to be seen whether they reflect internal representations of action symbols, a possibility which could be assessed using the criteria in this study” (lines 811 to 814).

### Clarifying the relationship between "action symbols" and "symbolic representations" more generally.

Clarifying the above points would help the reader to better understand the relationship "action symbols" (a novel construct) and “symbolic representations” more generally. The present lack of this distinction is especially evident around L.114, where for the different behavioral phenomena cited - handwriting, drawing, object manipulation., dance etc., it is not clear that they are really using action symbols, as the authors define them, in particular. This is related to a line in the General Discussion (around L.624) where the authors seem to imply the existence of different kinds of symbol systems, all of which rely on discrete units. It would be helpful to clarify early on what makes an action symbol distinct from other symbolic representations and on the basis of those properties what kinds of phenomena may or may not be thought to use these representations.

We appreciate the reviewer’s request to clarify our terminology. A defining feature of an action symbol (as opposed to another kind of internal symbolic representation) is that it ultimately represents a motor act. We made this point previously in defining action symbols as “internal, recombinable representations of discrete units of motor behavior” (see Abstract), reiterated in the Introduction: “symbolic representations in the form of discrete units of action (action symbols) that are recombined into sequences”, and in the Discussion paragraph noting that action symbols are a basic kind of symbol compared to the full range of abstract cognitive symbols thought to underlie (at least) human cognition (lines 702 to 719). Realizing from the reviewer comments that additional clarification was necessary, we have revised the text to further clarify this distinction between action symbols and symbols broadly.

- Lines 711 to 714 (relevant text in bold): “It remains to be determined whether and how the neural substrates identified here in **motor tasks** relate to other proposed kinds of symbols, including those implicated in more abstract tasks, and those with properties hypothesized to be unique to humans”

We also note that the study of symbolic representations at the neural level is in early stages without established frameworks, and so an important contribution of our study is an empirical framework based on specific symbolic properties in behavior and neural activity. Our hope is that this will spur future

studies that empirically clarify the relationship between the action symbols here and other kinds of symbols. We have added this point to the Discussion:

- "Our study's criteria of invariance, categorical structure, and recombination may be useful in future studies of symbolic neural representations, including in reassessing prior findings of invariant activity in cognitive tasks." (lines 716 to 719).

### Relationship to visual categories and visual scene representation
The primate visual system possess well studied systems for extracting "symbol-like" representations from visual inputs that seem to also meet the standards laid out for action symbols — visual object concepts are defined by their invariance over a variety of visual perturbations, display signatures of categorical structure, and visual objects can be represented in consistent ways even when embedded in different visual scene contexts.

Yet it seems to be critical to the argument in this paper that the representation in PMv is not driven by visual input, but instead encodes planned actions. Here is a passage in the Discussion to this effect: "...evidence already suggests that the circuit mechanisms underlying action symbol representation in PMv depend on local processes within PMv, as opposed to being driven entirely by image properties in a feedforward manner, or by symbol representations inherited from another region. First, as noted above, we found that PMv was not driven by image properties, but instead encoded the planned action."

What direct evidence is there for these claims in the current study? Why would it not be plausible for the structure observed in PMv to have been driven by "symbol extraction"-like mechanisms in visual cortex that operate upstream of action planning? Moreover, what is at stake / lost by assigning credit to more posterior representations more strongly associated with sensory processing for the kind of flexible "compositional generalization" observed for drawing the multi-stroke targets? Notably, there are no electrophysiological measurements conducted in these posterior areas, making it difficult to resolve whether similar neural signatures for action symbols might be decodable from such posterior representations.

We thank the reviewer for this important point. First, our claim that "PMv was not driven by image properties, but instead encoded the planned action" is based on the finding that, for tasks with ambiguous images, PMv encodes the planned action, and not the image, and that we did not detect an action symbol representation in vlPFC, one likely major relay of visual object representation to PMv. Based on these points, we suggested that the action symbol representation in PMv was unlikely to be completely inherited from upstream areas.

Motivated by this reviewer comment, to further strengthen this argument, we have performed additional experiments showing that PMv is not directly driven by the visual properties of the shape that the subject is visually fixated on; instead, PMv encodes the primitive the subject is planning to draw.

- This is added in **Extended Data Fig. 15.** See the description of this new figure above (The paragraph starting "To refute the reviewer's suggestion that an action symbol may be directly tied to its associated visual features…").

Second, we agree with the reviewer that we cannot (and do not mean to seem to want to) rule out that other inputs to PMv, especially those we are not recording from and including visual processes, may contribute meaningfully, and we agree that this possibility would not detract from our findings in PMv but

instead would raise important questions for future work. In light of this suggestion, we have revised the pertinent Discussion paragraph to now describe the rich motor, cognitive, and visual signals that converge onto PMv. One purpose of this revision was to convey PMv's unique position at the intersection of visual (dorsal and ventral stream), motor, and cognitive signals. Another purpose was to note the possibility that visual processing may play a role.

- The new paragraph is in lines 756 to 773, which includes this sentence: "PMv receives input from the two main visual pathways: (i) spatial- and action-related "dorsal stream" signals via inferior parietal cortex, and (ii) shape- and object-related "ventral stream" signals via vlPFC."

Arguably the actual stroke primitives themselves are exactly the overlearned stereotyped movements that the manuscript takes care to distance this study from (L683-L705) in the Discussion, and what really distinguishes this behavior from other cases is the ability to generalize to novel and more complex visual targets. What are we missing?

We agree that at the behavioral level, our study's key advance is showing that subjects flexibly generalize, especially in how they can in-the-moment recombine learned primitives to draw novel characters. This finding is one of the key pieces of evidence for action symbols, and, to our knowledge, is unprecedented in previous paradigms. The capacity to generalize reflects a powerful cognitive operation on primitives, even if the primitives were learned through substantial practice, just as it does for humans composing new sentences in language, or new sketches in drawing. Because the finding of generalization is so important, we have updated the Discussion to further clarify that, while it is true that prior studies have also studied motor primitives and behavioral units, an essential advance of our task is compositional generalization:

- Lines 776 to 789: "Our study introduces a paradigm to study compositional generalization…"
- Lines 813 to 814: "For both motor primitives and syllables, it remains to be seen whether they reflect internal representations of action symbols, a possibility which could be assessed using the criteria in this study."
- The paragraph in lines 791 to 803, starting with: "This drawing task is complementary to prior tasks in neural studies of motor behavior, which, to our knowledge, have not directly addressed compositional generalization."

### Re-examining support for recombination

For the neural analyses, the authors report similar representations for primitives whether they are drawn in a standalone fashion or as the first primitive in a composite character. In L.657-L.664, the authors begin by highlighting a finding about PMv that closely dovetails with findings in this paper but try to distinguish the current findings by emphasizing the categorical and recombinatorial properties observed. While category sensitivity isn't unique to PMv (see notes on visual categories above), I see the argument as "PMv is the only area that exhibits all 3 properties (invariance, categorical structure, recombination)." While the authors show similar neural signatures for primitives when drawn alone or as the first sub-part in a character, this does not seem to be evidence for recombination. A hallmark of recombination should be that the production of primitives in a character reuse the representations from when they are produced in a standalone manner. The justifications for why subsequent primitives in characters were not compared are reasonable, but as it stands the takeaways with respect to recombination are limited.

The present argument seems to be based on the results from the behavioral analyses of stroke trajectories, which are clearly related but are not directly germane to arguments about PMv. The authors should clarify how the present neural results constitute evidence for recombination.

The reviewer is correct to emphasize that importantly PMv exhibits all three properties in combination. We add that our argument is based, additionally, on the finding that PMv is the area that most strongly exhibits each of these properties alone (**Figs. 4-6**).

We agree with the reviewer that "a hallmark of recombination should be that the production of primitives in a character reuse the representations from when they are produced in a standalone manner". To strengthen this argument, as per the reviewer's request, we performed additional analyses of all strokes during character drawings. The results confirm those from the original manuscript's analysis of the first stroke in characters; specifically, PMv activity is reused from Single-shape tasks when a primitive is drawn embedded in a character.

- This is a new figure: **Extended Data Fig. 14.**
- And corresponding text added to Results in lines 599 to 609, starting with "In the preceding analysis, to account for the fact that Single-shape strokes…"

We note that, in performing this analysis, we found a generic effect of whether a given stroke was the first stroke in a sequence vs. one of the later strokes. In extensive analyses, we found that this effect on neural activity was simply an additive component and that it was the same across all task types (Single-shape, Multi-shape, Character). We therefore reasoned that it likely reflects the motor aspect of the large initial reaching movement that is present only for the first stroke (from the start button to the drawing). In the new analyses that include the non-first character strokes, we performed a simple correction by subtracting out this component from neural activity (for "first stroke" trials), before performing the comparison between Single-shape and Character tasks.

- We added figures showing the additive effect of "first stroke" on neural activity (**Extended Data Fig. 14**), and the rationale for this correction is described in the corresponding figure legends.

Finally, also analyzed additional experiments testing whether PMv reflects recombination of primitives in the Multi-shape task. Just as for the Character task, we found reuse of PMv activity in the Multi-shape task. This strengthens our conclusion by both replication in a new dataset and by generalization across task types.

- We added this result to the new figure (**Extended Data Fig. 14**).

Together, these multiple results support the conclusion that PMv activity reflects recombination of primitives, a hallmark of an action symbol representation.

Additionally, for the stroke trajectory analyses, while it is interesting that the strokes used by each monkey in the character task are aligned to their own primitives, it is not clear why an emphasis is placed on action symbols being individual-specific. Since the monkeys were strongly disincentivized from deviating from strict stroke patterns during the primitive learning phase perhaps this result is a partial artifact of that. There should be more discussion of these results as it relates to the extensive training procedure for the primitives.

We thank the reviewer for highlighting the need for a clarification on how the ability to recombine primitives in characters relates to the significant training that went into initially learning the primitives.

We do not think that this recombination ability should be considered an "artifact" of extensive training on single primitives, because this training cannot explain how subjects generalize to novel character tasks. Indeed, it is clear from prior studies that even simple forms of generalization are not trivial feats. Consider an example study where animals overtrained on reaching movements failed to generalize to simple manipulations such as reaching to a different angle (Kondapavulur et al. NatComm. 2022). Similarly, consider one of the only prior drawing-like tasks in macaques (Averbeck ExpBrRes 2003). There, monkeys took 1-2 years to learn four shapes, and even then failed to generalize to draw new shapes (B. Averbeck, personal communication). In our case, monkeys spent less time training on primitive shapes (weeks, excluding the time taken for initial training on task structure), and can readily generalize to draw new complex shapes. We believe that this remarkable ability to generalize is only possible because they learned a compositional strategy (action symbols). We have now further emphasized this in the Discussion:

- The paragraph in lines 791 to 803, starting with: "This drawing task is complementary to prior tasks in neural studies of motor behavior, which, to our knowledge, have not directly addressed compositional generalization."
- We also revised the paragraph starting with "Our study introduces a paradigm to study compositional generalization." (lines 776 to 789) to better describe the properties of our task which makes it well-suited for studying generalization, and which distinguish it from prior task paradigms.
- Finally, we make a specific reference to the prior drawing-like task in macaques as a contrast to the extensive generalization ability in our animals: "Note that this lack of direct instruction contrasts with prior drawing-like studies in macaques, where the visual cue was a moving cursor instructing how to move the finger or was one of two to four highly-practiced images to which memorized joystick movements were learned." (lines 783 to 786).

The reason we highlighted that primitives were individual-specific is that this (coincidental) fact gave us a powerful tool for analyzing behavioral signatures of primitive recombination. We have now performed additional behavioral analyses which do not rely on individuality, but instead compare each subject's primitive-match-rate to a null hypothesis based on simulating primitives by "remixing" each subject's own primitives.

- These results are in an updated **Fig. 2p**.

It is also important to clarify that the specific primitives learned by each subject were not "artifacts" of the training procedure. In fact, the subjects were not "strongly disincentivized from deviating from strict stroke patterns during the primitive learning phase". Instead, during primitive training, scoring was based on the final image similarity, and not on the stroke trajectory; therefore, subjects were allowed to use variable stroke trajectories if they wished. Due to their own biases and idiosyncratic learning trajectories, they each converged on their own set of unique primitives.

- We have emphasized this point in the main text: "Although we did not impose rules for what spatio-temporal trajectory (i.e., stroke) should be used for a given shape…" (lines 126 to 127).
- And to Methods: "We did not constrain subjects to learn specific stroke trajectories for each primitive; therefore, differences between primitives reflected each subject's own decision for how to draw each shape." (lines 952 to 954).

### Clarifying the process by which the stimulus set was generated
It was not entirely clear how the specific shapes used for training were generated. Could more detail be provided concerning the rationale for arriving at this particular set of shapes, and why there are no

more and no fewer? What can be said about the degree to which these shapes provide good coverage over the set of possible shapes that could have been used in this study?

We designed this set of shapes to intuitively cover a variety of trajectory profiles (by varying rotation, the number of direction changes, and whether they were curved or linear), and yet simple enough that multiple shapes could be combined into single character images.

We also note that the conclusions of our study do not depend on the particular set of shapes (or on using an "ideal" set of shapes). Our study depends on subjects successfully learning primitives associated with the basic shapes, and then demonstrating behavioral generalization using these primitives.

Both of these points have been added to the Methods:

- "We chose this set of shapes in order to cover a variety of trajectory profiles (by varying rotation, the number of direction changes, and whether shapes were curved or linear), and yet to still be simple enough to draw with one stroke and to be able to combine multiple (two to six shapes) shapes into single character images" (lines 949 to 952).
- "We also note that our study did not depend on using an "optimal" set of shapes, but instead depended on subjects learning primitives associated with the basic shapes, and then demonstrating behavioral generalization using these primitives, as we found that they did" (lines 954 to 957).

### Potential impact of experimenter demands on learning stroke primitives
The methods seem to imply that 'sub-strokes' were heavily penalized "we also provided feedback online by immediately aborting a trial if subjects made serious errors, including (i) if they touched a position far from any image points, and, (ii) for single-shape and multi-shape trials, if they used more than one stroke per shape in the image." This seems to imply that the monkeys were highly disincentivized from producing anything outside the primitives. What bearing does this have on the different results reported? Would we have not observed as strongly categorical primitives and re-use of the same in the character tasks under a more lenient training regime?

First, we would like to clarify that these constraints did not directly lead to a situation where subjects "*were highly disincentivized from producing anything outside the primitives*". For Single-shape and Multi-shape tasks, the constraints were minimal, and just there to incentivize subjects to stay reasonably close to the image, and to use one stroke per shape. They still had freedom to vary the stroke trajectory, including the (i) direction, (ii) onset location, and (iii) other kinematic details. This freedom is evident, for example, in the fact that the two subjects learned different stroke trajectories given the same shapes and constraints (**Fig. 1e-g**), and that during learning, subjects exhibited variable stroke trajectories before stabilizing to the final trajectories [data not shown].

- We have emphasized this point in the main text and Methods (see our response to the comment above that starts with "Additionally, for the stroke trajectory analyses…").

We also note that this constraint was specific to just the Single-shape and Multi-shape tasks (where the shapes were spatially separated in the image), and was not imposed in the Character task, where subjects were allowed to use strokes that did not match the image shapes (i.e., a single stroke could span multiple shapes in the image). The fact that subjects still reused their primitives, and did so on novel complex images, is strong evidence for primitive recombination (**Fig. 2k-p**).

- We have updated the schematic in **Fig. 2k** to emphasize that we did not prescribe just a single "correct" stroke sequence for character tasks.

The other two main behavioral findings—invariance and categorical structure—are also not trivially explained by the hard constraint requiring a single stroke. This is because these findings were based on immediate generalization to novel images (see "Novel" label in schematics in **Fig. 2a** and **Fig. 2f**), and subjects did not receive higher reward for reusing their primitives. In fact, for the categorical structure experiment, they would have been rewarded *more* if they traced the image in detail instead of using their primitives.

That said, we agree that training constraints surely played important roles in the learning of primitives. Our intuition is that we found the right balance of constraints vs. practice on many tasks that are variable yet structured. To test this would require long-term experiments manipulating constraints across subjects and assessing the effect on learning and subsequent ability to generalize. These experiments are beyond the scope of this paper, but would be interesting for future studies.

We also note a similarity to skill learning in humans. Hard constraints on initial learning would be consistent with how many human skills are initially learned, even skills that, once learned, exhibit remarkable flexibility and generalizability, such as handwriting, musicianship, and athletics.

### Clarifying the degree to which the tasks studied here constitute a novel paradigm distinct from established motor tasks.
The class of behavioral tasks reported in this paper appear to be less of a novel category of tasks than one that combines aspects of some of the earlier ones mentioned (instructed movements / working memory guided movements / minimally restrained movements). While the evaluation tasks are fairly unrestrained, the training tasks aren't and adhere more to classic 'instructed movement' tasks. These strongly learned movements might themselves strongly constrain the space of possible results that can obtain on the various behavioral generalization tasks. A clearer distinction of the current paradigm that doesn't reduce to a 'sum of previous methods' should be presented if the authors choose to emphasize the novelty of this paradigm

We thank the reviewer for highlighting the need for us to demonstrate the novelty of our approach. We believe our approach represents a fundamental advance relative to previous task paradigms. Specifically, it is the first paradigm, to our knowledge, to study *compositional generalization*. This point was previously conveyed in the Discussion, but not clearly enough (original lines 683 to 705, paragraph starting "We introduced a task paradigm testing compositional generalization by recombining action symbols into novel sequences…"). We now, in light of the reviewer comment, suspect that this could easily be interpreted as a "sum of previous methods" because we listed specific features of this paradigm that are critical for compositional generalization (original lines 683 to 687, starting "This task has three critical design features…"). Instead, we now clarify that there is a single fundamental advance (compositional generalization), and those features were critical for achieving this advance. Importantly, compositional generalization has not been tested in prior tasks that used instructed movements, working memory guided movements, or minimally restrained naturalistic movements (as we now note in the paragraph starting "This drawing task is complementary to prior tasks in neural studies…" (new lines 791 to 803).

- We have now revised a Discussion paragraph to clarify that the task's key innovation is in introducing a way to study compositional generalization. This is the paragraph starting "Our study introduces a paradigm to study compositional generalization" (lines 776 to 789).
- We highlight here a key point we made in that revised text, which is that there are two key features that enable the probing of compositional generalization (see lines 776 to 789 for details):
    - First, visual cues (images) do not directly instruct movement details, instead requiring the subject to infer what stroke movements to make. This led them to learn "compressed" representations of action (i.e., action symbols).
    - Second, there was substantial flexibility in stimulus generation, with a large pool of possible images that can be varied systematically and continuously to test generalization behavior that is diagnostic of three independent hallmarks of symbolic representations: invariance, categorical structure, and recombination.

As a contrast, consider the closest previous drawing-like task, Averbeck et al., ExpBrRes 2003. There, monkeys took 1-2 years to learn four shapes, and failed when asked to generalize to draw new shapes (B. Averbeck, personal communication). So, compositional generalization was not tested. In our case, monkeys spent less time training on more primitive shapes (weeks, excluding the time taken for initial training on task structure), and can readily generalize to draw new complex shapes by composing those primitives.

- This has been added to that Discussion paragraph: "Note that this lack of direct instruction contrasts with prior drawing-like studies in macaques…" (lines 783 to 785)

At the same time, we acknowledge that this task is certainly inspired by and has some overlap with prior approaches. We have revised the Discussion accordingly.

- First, we have modified the entire paragraph about "motor primitives" to be more open to the possibility that prior findings of units/segments/motifs in motor behavior may be related to the action symbols in our study. This is in the paragraph now starting with "Prior behavioural studies suggest that continuous motor behavior can be decomposed into reused segments…" (lines 805 to 814)
- Second, we have added more references in the paragraph on prior evidence for invariance in PMv, to better acknowledge the breadth of studies of this brain region. See the paragraph starting with "Prior evidence for motor invariance in PMv may reflect action symbols related to those identified here…" (lines 737 to 754).

### Bridging the theoretical/modeling paradigms laid out in the introduction/Discussion (symbolic, neural, and dynamic systems approaches).
The argumentation is that the decodability of theorized action symbols in PMv hints at the possibility of programs as being represented in neural substrates too (e.g., REPEAT(◯, 3)). But this is a bit of a leap, as there is no evidence for anything beyond simple stroke primitives in the present neural data. Either this section needs further elaboration delineating how the present results might reasonably be extended to evidence for programs or the claims should be tempered.

We thank the reviewer for raising this important question about whether and how the concept of neural programs (as a mechanism for compositionality) fits into the overall paper. We agree that the neural findings were restricted to action symbols, and did not directly show evidence for programs. However, we think that raising the idea of programs is important because it clarifies how symbols could actually

work to support compositional generalization. It clarifies that compositional generalization involves both symbols and mechanisms for composing them, and that programs are a hypothesis for those mechanisms. Without this, the reader may be confused as to what neural mechanisms could possibly link these two abstract concepts of action symbols and compositional generalization. Therefore, we believe it is useful to briefly allude to programs.

In the Introduction, we were careful to focus on symbols and had only one (indirect) reference to programs: *"Symbols support generalization because they enable the systematic derivation of a large set of possible representations from a smaller set of components, such as new animals imagined as rule-based combinations of discrete parts (e.g., new animal = 1 torso + 8 arms + 4 legs)." (*in lines 56 to 58). We would hope to keep this sentence, as it gives a useful example for how symbols could support compositional generalization.

In the Discussion, we raise the idea of programs only in the last paragraph, and we frame this as speculative by using phrases such as "possibly by implementing symbolic-program-like operations" (line 821) and "Looking forward…" (line 823). We would prefer to keep this point because it is critical to the topic of this paragraph (how the identified neural representation in PMv could potentially bridge our understanding of symbolic and neural processes).

We hope the reviewer agrees with our rationale for keeping these brief references to programs in the Introduction and Discussion.

We do agree that the specific reference to a "REPEAT CIRCLE" program may have been too speculative. We have therefore removed this specific example from the last paragraph of the Discussion.

## Specific comments

Line 1079: "95% CI" is not the correct term to use here to refer to the interval defined by the 2.5th and 97.5th percentiles of the data distribution. The CI is used for the interval defined by those thresholds over the sampling distribution of a statistic.

We thank the reviewer for spotting this mistake. We agree and have updated this in line 1188:

- "in particular, if the distance was within the interval bounded by the 2.5th and 97.5th percentiles of the distribution of trajectory distances."

L67. What are 'these abilities' referring to? Compositional Generalization abilities?

"These abilities" refers to "abilities" used in the preceding sentence: "abilities, such as drawing, which are not superficially symbolic, but which seem to reflect internal symbolic representations". We believe that the phrase "span various domains" in the phrase "These abilities span various domains" may explain the ambiguity, so we have replaced that phrase with: "These abilities include…" (line 62)

L78. Why these 3 properties specifically (invariance, categorically, recombination), is this well established?

The prior literature on putative symbolic representations in human and animal cognition has led to a common understanding that, essentially, symbols are internal representations of discrete units that are systematically recombined (see references in Introduction). We operationalized this definition of symbols into the three independent testable properties of invariance, categorical structure, recombination. This rationale was described in the Introduction ("*Given that symbols are discrete units that are systematically recombined, a neural population representing symbols should exhibit three basic properties in its activity patterns*", new lines 71 to 73). In the Discussion (paragraph starting *"Identifying a symbolic neural representation in monkeys may provide general insights into the neural basis and evolutionary origins of symbols"*, lines 702 to 719), we again suggest that this may be a general property of symbolic representations, but we also note that some symbol systems may have other, perhaps more abstract, properties as well (that same paragraph).

L97. No stats reported for Fig 2d./2e.

Thank you for catching this — Fig. 2e has been updated.

L291.Does scoring 'images' mean using Hausdorff distance? Wasn't clear here.

This was indeed not clear. We have updated that line to clarify that "scoring images" uses the image distance (which is based on the Hausdorff distance): "...applying this metric to score the images (using image distance instead of trajectory distance; see Methods)...produced a linear relationship with morph number." (line 304)

L605.The line about PMv showing visual invariance seemed a bit out of left field.

We agree that this line needed better motivation. We have moved this sentence into a new second paragraph in the Discussion, which summarizes our evidence for PMv being not directly visual nor motor, but instead reflecting a relatively abstract representation.

- This new paragraph is in lines 722 to 735, starting "Several lines of evidence indicate that this action symbol representation in PMv is abstract, not directly driven by visual features or motor parameters."

Fig 4 k. It is interesting that best decoding accuracy for primitives (in PMv) is higher than decoding accuracy for location in any area. Given that decoding location (4-way classification) seems like an easier task than decoding primitives, of which there are many, could the authors elaborate as to what a reader should conclude about the representation of location generally in both motor and planning areas of the brain?

This is a great observation, and we missed the potential difficulties a reader might encounter. We are wary of making any conclusions based on comparing the precise values of the decoding accuracies of location and primitives. One reason is that this may be affected by the particulars of our paradigm, such as the distance between the locations. Instead, we think it is more rigorous to draw conclusions based on comparing between areas their relative primitive vs. location encoding strengths, which is how we derived the conclusions of Fig. 4. That said, we think this is an interesting observation worth pursuing in the future with paradigms that more systematically manipulate location and primitives.

L610. The first paragraph of the Discussion closes with the following two sentences: "This finding of a localized representation of action symbols in PMv indicates an important role for symbols in producing novel, goal-directed action sequences. More broadly, this finding supports the hypothesis that cognition can be understood, at the algorithmic level, in terms of internal symbolic operations…" The latter sentence is overly broad in its claims about "cognition" and does not obviously follow from the first, nor from the set of findings here.

We understand the reviewer's concern. We intended this sentence to foreshadow the last paragraph of the Discussion, which is more forward-looking on how the findings here give insight into symbolic operations more broadly in cognition. But we agree that this sentence may be misconstrued as asserting a conclusion, and so we have removed it.

In the present work, symbols are acquired through intensive training. Does learning any form of action symbol require such laborious learning or are there alternative ways that such action symbol representations might be acquired by primates? It would be helpful to comment on the learning of such symbols in both humans and non-human primates and where convergences and differences might lie.

Many skilled motor behaviors require extensive learning and practice, even those exhibiting flexibility and generalization once learned, such as handwriting, drawing, speech, and abilities in sports and music, and we expect that many of these skills involve action symbols (although this remains to be directly tested in neural studies). At the same time, we don't think there is anything inherent to action symbols that requires extensive learning. Indeed, studies of naturalistic motor behaviors in various animals suggest the presence of discrete units in motor behavior, and some of these may be innate, although whether these units support compositional generalization or reflect symbolic representations remains to be tested.

- We have added this to Discussion, in lines 821 to 814: "For both motor primitives and syllables, it remains to be seen whether they reflect internal representations of action symbols, a possibility which could be assessed using the criteria in this study…"

Some relevant references are missing with respect to investigations of the neural computations underlying compositional generalization. Here is one using somewhat different methods in humans, but appears to be relevant to the overarching argument: Schwartenbeck, P., Baram, A., Liu, Y., Mark, S., Muller, T., Dolan, R., ... & Behrens, T. (2023). Generative replay underlies compositional inference in the hippocampal-prefrontal circuit. Cell, 186(22), 4885-4897.

We agree that this work is relevant in indicating a potentially important role for hippocampal replay in constructing compositional representations, which may engage with the PMv representation identified here. We have added it, along with related work, to the Discussion:

- We added these references to the line "...and in hippocampal systems implicated in generating compositional representations…" (lines 826 to 827).

Additionally, it is a strong claim to argue in the introduction that these other phenomena rely on the hypothesized construct in the present paper before results supporting the presence of the construct have been presented.

We did not notice this, but now understand the reviewer's concern and agree with it. We clarify that the purpose of referencing these phenomena was to suggest that there is already behavioral evidence

across a variety of abilities for the presence of internal symbolic representations. We did not intend to suggest that symbolic representations must exist across all these domains. Indeed, we call the idea of symbolic representations a “hypothesis” (line 58) and state that “we lack definitive evidence for whether and how symbols are implemented in neuronal substrates (line 67)”.

- In addition, we have now replaced “Despite behavioral evidence **for** internal, symbolic representations” with “Despite behavioral evidence **suggesting** internal, symbolic representations”. (line 67)

Referee #2 (Remarks to the Author):

I co-reviewed this manuscript with one of the reviewers who provided the listed reports.

Referee #3 (Remarks to the Author):

This is an extremely ambitious project that will be interesting to a broad readership within neuroscience and cognitive science. The authors find that when drawing complex shapes, macaques sequentially represent discrete strokes which are idiosyncratic to each subject, and these representations are invariant to location and size of the stroke on the drawing surface, as well as the order of strokes within a shape. The authors identify neural correlates of these representations during the planning period in the ventral premotor cortex (PMv). They show extremely convincingly that the neural representations in PMv represent the primitive categorically, in a manner that is invariant to low level motor details, and in a manner that can be reused in different combinatorial plans. Three critical factors that are required for symbolic-like processing.

The article is easy to read, and builds a strong case for an exciting finding that is strongly empirically supported. The task that the authors designed, the coverage of recording sites and the established results are all outstanding features. It is particularly impressive that the authors have established a rich compositional task for primates that even allows such rich questions to be asked. The vast majority of previous electrophysiology tasks engage much simpler behaviours where it is hard to know what is overtrained, and often hard to dissociate the roles of different brain regions, particularly within the frontal lobe. This task is extremely imaginative and impressive because the space of possible problems that can be given on different trials is vast, but yet the task is amenable to formal computational description - because of its compositional nature. It is a beautiful achievement.

The strength of the manuscript is that authors focus on a very targeted part of the problem - the representation of action symbols — and provide extremely strong empirical evidence for these particular claims. They describe this evidence very thoroughly, leaving little room for doubt (hence see limited questions below). They also show that the claims are limited to PMv amongst many frontal regions. It is striking that they can identify neural representations of actions that are so consistent with regard to the shape and velocity, and yet are invariant to the location, size and "part-whole" relation to a bigger shape. The testing of ambiguous stimuli helps a lot with ensuring that the authors really are capturing motor activity and not perceptual features. The finding sits well within the context of previous PMv literature on object grasping, and extends it dramatically both computationally and functionally. It also sits naturally with PMv's anatomical location at the junction of the dorsal and ventral streams.

We thank the reviewer for this very favorable assessment.

If there are weaknesses of the study, they are the corollaries of the strengths. Of their 16 recording sites, the authors effectively provide an account of the neural activity only in the PMv. Because of this, they cannot give a theory of more abstract representations that are taking place during this task (how is the complex shape is represented? how the parsing of the complex shape into simpler shapes is performed? Are there representations of the sequence of actions?). This is especially unfortunate, as neither the code nor the data are available, making (i) independent testing of the representation hypothesis in their data impossible and (ii) delaying further investigation of these exceptionally interesting questions.

Overall, my view is that this article represents an extremely unusual achievement and should be published by any journal to which it is submitted. My main comment is about the choice of scoping the analysis to the author's action symbols, but I can see this being a trade-off to provide a very meticulous

understanding of these action symbols in the PMv, and on those I have no major comments (see below for minor comments).

We thank the reviewer for this positive assessment. We are also grateful to the reviewer for highlighting one point, which we have clarified in the revision: we understand now how the impression that "*the authors effectively provide an account of the neural activity only in the PMv*" came about. We need to emphasize that all recordings and all analyses were performed identically for all recorded areas—it just turned out that in each case the quality in question was found the strongest in PMv. Therefore, example units and population activity illustrating a given property always include PMv (Figs. 4a-d, 5b-e, 6a-b). But we then proceed to perform analyses on all areas and summarize them in comparison to each other (Fig. 4jk, 5g, 6f). That said, because it turned out that PMv exhibited all of these properties the strongest, and thus turned out to be the site of action symbols (the conceptual focus of our paper), PMv assumes a central role in all sections of the manuscript, thus giving rise to the reviewer's impression. To avoid making this impression on readers, we have made the following revisions:

- Headers for the Results sections on the neural findings (for Figs. 4-6) have been revised to place the property in question (e.g., motor invariance) in the topic position of the sentence. Compared to previous, when "PMv" was instead placed in the topic position, the headers now don't give the impression that we started out analyzing only PMv. For example, the header for Fig. 4 was previously "PMv encodes stroke primitives in a manner that is invariant over location and size", and is now "Motor-invariant encoding of stroke primitives in PMv".
- In the first Discussion paragraph, we now emphasize that we tested all areas for these three properties, and as a result of this, identified PMv as the area where those properties converged: "In neural recordings, we tested for each of these three properties in population activity across multiple motor, premotor, and prefrontal cortical areas (Fig. 3). We found evidence for each of these three properties, and, strikingly, each property was strongest in a single area, ventral premotor cortex (PMv). This reveals that activity in PMv, but not elsewhere, encodes primitives in a manner that reflects the conjunction of three hallmarks of symbolic representations…" (lines 674 to 679).

Minor Comments

The choice of signal processing steps applied to the touchscreen data seemed overly complex. If I understand correctly, the data is acquired at 60Hz. In order to estimate velocity, the data is then (i) upsampled to 500Hz, (ii) low-pass filtered to 15Hz, (iii) "epoched" based on the upsampled data, (iv) low-pass filtered to 12.5 Hz, (v) downsampled to 25Hz, (vi) a numerical estimation of the signal's derivative is performed, and (vii) upsampled to 500Hz. Because virtually all of the results afterward rely on an accurate estimation of action symbols from the touch data, the choices here are very impactful. I think that providing an explanation for the choice of these steps will help the reader understand why, e.g., data was low-pass filtered twice in a row, and how robust the results would be to different preprocessing steps.

The reviewer correctly points out that the preprocessing steps appear overly complicated. The reason for this was that the preprocessing pipeline had a modular structure, with separate modules for acquisition, which automatically upsamples touch data to match the sampling rate of all the behavioral signals (500Hz), and smooths the data to reduce noise above the maximum frequency of the drawing movements (15Hz), and segments the data into strokes (steps i, ii and iii). For analyses that use velocity data, a second module converts these positions to velocities (steps iv, v, vi, viii).

- We updated the Methods to explain the logic for choosing those frequencies (lines 1106 to 1111) and to clarify that there were two separate processing modules, one for stroke extraction and one for velocity estimation (lines 1106 to 1120).

The color-coded statistical significance of the author's Fig 5j, 6g and Extended data Fig 5b is missing information: What do "low" and "high" mean here? We learn in the methods that there is a mapping between a Bonferroni-corrected two-sided t-test and the colors, but we don't know what the mapping is, or what the order of magnitude of the p-values are, etc.

Thank you for pointing out this oversight. We have added a description to the figure legends noting that the “low” and “high” correspond to no (0) and maximum (7) other brain regions beaten (in the statistical test comparing each pair of brain regions):

- *“Low” to “High” ranges from no (0) to all (7) other regions “beaten”)* has been added to line 494 for Fig 4j, and similarly for the other figure legends.

We have also added plots showing the exact p-values for all pairs of brain regions:

- For Fig 4j and Extended Data Fig 5b (which is now Extended Data Fig. 10b), p-values are now in **Extended Data Fig. 8**.
- For Fig. 6f, p-values are now in **Extended Data Fig. 13**.

Looking at Figure 1, it appears that one subject prefers systematically tracing starting from the left, while the other prefers starting from the right. This makes the set of stereotypical action symbols particularly different across primates and helps subsequent analyses. But it is also a striking feature that is not discussed in the article. Were there pre-training steps to get that? Are the animals tracing with the same hand? Were there more subjects in behavior, and those were selected to acquire neural data? Or is this just a fortunate coincidence?

Indeed we capitalized on these idiosyncrasies for our analysis of behavioral recombination in the Character task. This difference was coincidental; we did not encourage this behavior in training nor select for this behavior by subselecting subjects. Both subjects used their left hand. Each monkey, on his own, developed his own set of primitives.

- We have noted this in Methods (line 952 to 954): “We did not constrain subjects to learn specific stroke trajectories for each primitive; therefore, differences between primitives reflected each subject’s own decision for how to draw each shape”.
- We have also added a new analysis of behavioral recombination that does not depend on comparing one subject’s strokes to the other subject’s primitives. This analysis compares each subject to a simulated “remixed” set of primitives based on his own primitives (updated **Fig. 2p**). The result confirms our conclusion that subjects reuse their own primitives when drawing characters.

This dataset is rich and the authors analysis only explore part of it (as the authors acknowledge in the discussion); this is particularly visible from the fact that almost all of the recorded areas appear to be performing task-related computations, but the authors' analyses are providing insight about the PMv. However, the authors have not made the data or the code available and, from the writing of the "Data and code availability" section, they are not intending to do so. Besides the fact that reviewers cannot offer a cursory review of the code as well as the article, this means testing hypotheses on this dataset outside of the author's lab will be much harder, hindering scientific progress.

We agree with the importance of releasing the code and data. We have done so, uploading those items to a Zenodo repository. It is currently private and can be accessed at this link (it will be made public upon publication):

[redacted]

- We have updated the “Data and code availability” statement accordingly.

In their discussion, the authors report as surprising that their action symbol representation is confined to the PMv (and not, for example, in the PFC or the MTL). But the authors' representations are identified using not the cinematic of tracing, i.e. both location and velocity. When they conclude that they did not find symbols in the dlPFC or the preSMA, what the authors mean is that the specific signature of velocity+location arrangement that they are looking for is not present in these areas; but perhaps this is because e.g. the dlPFC contains a more abstract representation (which does not encode velocity, etc.)?

The reviewer is raising an important point that requires clarification. We believe that our main analyses appropriately tested the possibility of a more abstract encoding of action symbols in dlPFC, or any other area, because these analyses treated action symbols as discrete classes, and not as stroke kinematics or trajectories. Specifically, although kinematics are used for the initial assignment of labels to strokes in Figs. 5 and 6, the decisive neural analyses in Figs. 4i-k, 5d-h and 6e-f all treat action symbols as discrete classes. Therefore, our results do suggest that even the abstract encoding of primitives (as discrete classes) is localized to PMv and not in PFC.

To further draw this distinction between encoding of kinematics vs. the primitive class, we performed additional analyses showing that PMv does not significantly encode generic motor kinematics of strokes. In contrast, primary motor cortex (M1) does.

- This is **Extended Data Fig. 16**.
- This is added to a new results subsection “PMV encodes primitives in a manner dissociated from both the shape in view and from motor kinematics.” (lines 638 to 666).

Figure 4: the authors do not plot the same PCs for PMv (PC2 and PC3) and dlPFC (PC1 and PC2). Then in Figure 5, the plot PC1 and PC2 for PMv. And then in Figure 6 they plot PC2 and PC3 again (in both PMv and preSMA). This does not impact the analyses (which rely on the first 8 PCs) but begs the question of why these PCs specifically were chosen. Similarly, different time periods are used to estimate PCs in different analyses, but no information is provided about the motivation for these choices.

The reviewer raises an important point. The choice of different PCs for the different trajectory plots was in order to visually emphasize dimensions with the strongest effects. We reasoned that this was acceptable, since there is no *a priori* reason to pick one PC over the other, and the final quantification was performed using all top 8 PCs. We do agree that it is worth clarifying, and therefore have added this sentence to the figure legends: “Here, we use PCs 1 and 2 in order to emphasize the main effect in dlPFC of location.” (line 482).

For the time windows, this is likely referring to the use of 0.05 to 0.6 s for Fig. 4 and 0.05 to 0.9 s for Fig. 5. This difference was because, for a subset of the experiments in Fig. 4, the duration for the planning window was between 0.6 s and 1.0 s across trials. To ensure that neural analyses did not overlap the "go cue", we used the minimal window duration (0.6 s) for the maximum of the analysis window. We have noted this in Methods: "...to avoid including data after the go cue, which, for a subset of these motor-invariance experiments, occurred 0.6 to 1.0 s after image onset…" (lines 1420 to 1421).

Similarly, indicating which shape number corresponds to which actual shape, would be very helpful to the reader in trying to understand e.g. specific features of Fig 4j. In fact, I am not entirely sure which shapes / primitives were included, and why, in some of the figures where a number is all that's referenced.

We agree, and have added icons of the primitives to all figures that refer to primitive classes: **Figs. 2d, 4, 5.**

Extended Data Fig. 5 is never referenced in the text.

We checked to make sure that it is. There is indeed a reference which was previously "In different sessions, using the same methods, we found that PMv population activity encodes primitives in a manner that is also invariant to size (Extended Data Fig. 5)." and which is now "Second, we found that PMv population activity during planning encodes primitives in a manner that is also invariant to size (Extended Data Fig. 10)" (lines 459 to 461).

Tim Behrens

Referee #3 (Remarks on code availability):

The data and code are not available. Clearly the authors would like to analyse it further. This is understandable given the vast scope of the project and the limited focus of the current manuscript within this scope. However, it is clearly also disappointing (and perhaps against NIH policy?). I recommend the editors and authors negotiate a time period within which it will be made available.

We have made the data and code available at [temporary link pending publication]:
[redacted]

Referee #4 (Remarks to the Author):

Overall evaluation:
In this study, the authors presented both behavioral and electrophysiological results from macaque monkeys that learned to trace/draw different stroke patterns on a touchscreen. By training monkeys to reproduce in one stroke a wide variety of visually presented simple geometric patterns first (horizontal/vertical lines, half/full circles, zigzags, etc.), the authors attempted to foster symbolic representations for these "primitives" of finger movements. To verify that such representations truly reflect action symbols, the authors performed analyses evaluating three requisite features of symbols: motor invariance across locations/stimuli magnitudes, (discrete) categorization of learned stroke patterns, and applied recombination (of primitives) when reproducing complex shapes.
The authors provided both neural and behavioral data suggesting motor invariance and sigmoidal/category-like responses to the ambiguous morphing of two "primitives". However, the former has already been established by previous studies, and the latter has little bearing on whether or how such "primitives" were actually utilized by the animals. Regarding recombination, this study offered tangential evidence in the form of "task invariance" such that a shape could be drawn similarly when drawn by itself or as part of a complex. Unfortunately, current data could not address either the neural mechanism of the stroke pattern "decomposition" required to complete a novel drawing task with primitives or the self-generated positional/ordinal relationships among the stroke primitives.
This work is truly ambitious, introducing a creative paradigm with great potential. The great effort it took to survey various cortical areas with closely placed microelectrode arrays should also be acknowledged. With admirable courage, the authors addressed a very important question of the field, that of symbolic representation which, as the authors rightfully surmised, is key to our understanding of problem-solving using learned knowledge, and of intelligence in general. That said, as things stand, current evidence remains insufficient to call what the authors observed "neural representations of action symbols".

We thank the reviewer for these thoughtful comments, and also for acknowledging that—notwithstanding some critical suggestions which we respond to below—this work is a conceptual and technical advance that targets a fundamental question in the neuroscience of cognition.

Below, we respond in detail to each of the reviewer's comments, including with new experiments and analyses. At the very end, we summarize the reviewer's concerns and the essential aspects of our responses. We are confident that we have addressed all of the reviewer's concerns and hope that the reviewer will be convinced that we have sufficient evidence to claim that we have discovered a neural representation of action symbols in PMv.

Major issues:
1. My first concern is whether/to what extent the macaques acquired a system of symbols that could be utilized to accomplish various tasks, judging by the animals' behavioral performance. The authors trained the subjects for many generalization tasks to show that the symbol system was well established. However, some of the evidence seemed ambiguous:

We wish to first address one possible source of the reviewer's concern regarding whether the behavioral evidence demonstrates that subjects learned action symbols. Understandably, given that this is a motor task, one's default assumption may be that the behavior can be explained by stereotyped motor habits and simple sensory-motor associations. Because we also had this concern, we went to

great lengths to perform behavioral tests that, we believe, cannot be explained by stereotyped motor habits and simple sensory-motor responses. In general, this is because generalization tasks were novel and different from the training involved in learning primitives. For example, consider generalization to draw novel characters using sequences of reused stroke primitives. This cannot be explained by motor habits—which predicts stereotyped, not novel, sequences—nor by learned sensory-motor associations—because the sensory input (image) is novel. Below, we present detailed responses that expand on why the behavior is consistent with action symbols.

(a) Macaques were trained to learn > 15 different stroke patterns. How are these primitives independent from each other (as would be expected for "discrete units" mentioned in the abstract)? From Fig. 2o, we could see that some primitives were used more frequently than others by subject 1, while according to the subsection "Task types/Character tasks" in Methods, a well-trained subject could use all the primitives with similar frequencies since the shapes in characters were sampled randomly. Another point against independence is that we could see many supposed "primitives" being decomposed into other primitives in Fig. 2l. Does this imply that the macaques only learned some of the primitives? If so, the authors probably could try to specify a minimum symbol set.

The reviewer suggests that non-uniformity in the frequency distribution of primitives (Fig. 2o) indicates failure to learn some of the primitives. However, this non-uniformity is consistent with prior studies of the usage frequency of elements in other behaviors. One well-studied example is natural language, in which word frequencies tend to follow a power law distribution (Zipf's law; see Yang PNAS 2013). This non-uniformity has also been described in numerous animal behaviors, and has been interpreted as an adaptive form of information compression (Ramon Ferrer-i-Cancho et al., Cognitive Science 2013). We therefore do not think that non-uniform frequency of primitive reuse is an argument against them having been learned as distinct units.

- We have added the following statement: "Note that the non-uniformity of this distribution is reminiscent of the non-uniform frequency distributions seen in language and other behaviors" (lines 258 to 259).

One reason the reviewer may expect uniformity is the expectation that success on characters requires using stroke primitives that match the specific shape components in the image one-to-one. The reviewer states that: "*a well-trained subject could use all the primitives with similar frequencies since the shapes in characters were sampled randomly*." and that "*we could see many supposed "primitives" being decomposed into other primitives in Fig. 2l*." We clarify that the task was not designed for testing whether subjects match strokes to shape components. In general, images were ambiguous in that there were multiple correct ways to parse them into primitives. For example, in Fig. 2l, the fourth image from the left was parsed into two "horizontal V"s by subject 2. An alternative correct parse could have been to use one "horizontal V" and two diagonal lines.

- To make it clear that characters have multiple possible parses consistent with reuse of primitives, we have updated the schematic figure to depict multiple possible solutions (**Fig. 2k**).
- And we added this to the main text: "Note that, due to ambiguity, there are generally multiple possible behaviors that exhibit primitive reuse." (lines 331 to 332).

We also note that the suggestion that subjects did not successfully learn primitives is inconsistent with our positive evidence for highly consistent stroke trajectories for each primitive (**Fig. 1e-g**), and with the new analysis showing that primitive kinematics are highly different from each other even at the single-trial level (see response to next comment).

Finally, we note that our study's conclusions do not depend on there being anything special about this particular basis set of images used to train primitives. What is important is that, whatever these images were, the subjects discovered stroke primitives for them (through practice) and then productively and systematically reused the primitives.

In summary, the critical hallmark of discrete primitives being recombined is that subjects can draw novel characters, and do so by reusing their primitives, both of which are supported by our data.

(b) Moreover, similarity/distance metrics, while useful in establishing behavioral/neural fidelity, could not directly tell us whether stroke patterns were truly acquired by the animal as independent action symbols (also note that there are certain off-diagonal regions in Fig. 2d with distance values as low as part of the diagonal).

We thank the reviewer for raising the observation that the pairwise trajectory distances between primitives are not uniform (i.e., some pairs of primitives have lower pairwise trajectory distance than do others). The reviewer interprets this to mean that primitives are not independent.

We first point out that the reviewer's suggestion that "*there are certain off-diagonal regions in Fig. 2d with distance values as low as part of the diagonal*" is not accurate. Instead, the figure shows that each primitive is actually more similar to itself than to the next most closely similar primitive—all values on the diagonal are lower than the values in its corresponding row). To further emphasize this point, we have performed a decoding analysis showing that each primitive is separable from every other primitive using single-trial kinematic data.

- This has been added as **Extended Data Fig. 2.**
- And text added to Results, in lines 134 to 136: "In a further assessment of across-trial consistency and across-shape distinctiveness using a decoding approach, we found that each shape's primitive was easily distinguishable from every other primitive based on single-trial stroke trajectories (Extended Data Fig. 2)."

Second, we emphasize that non-uniformity in the distances between pairs of primitives is not unexpected. For example, for handwritten characters, it is common to write "d" and "a" with similar motor trajectories, but "d" and "a" are distinct motor concepts (probably represented as action symbols). A matrix of pairwise trajectory distances between all pairs of letters of the alphabet would show non-uniformity, similar to our matrix in **Fig. 2d.** Such non-uniformity is also present when considering kinematic distance between phonemes in speech (Willett Nature 2023).

We have also performed an analysis of neural data similar to the above analysis of motor kinematics, and found that primitives are separable from each other in their single-trial population activity in PMv:

- This is a new figure, **Extended Data Fig. 12**, accompanied by visualization of single-trial data in a low-dimensional embedding using UMAP, showing that primitives are separated from each other in PMv, but not in other areas (**Extended Data Fig. 7**).
- This is added to a new paragraph in Results, starting with "Categorical encoding of primitives implies that every primitive is associated with a distinct activity pattern." (lines 543 to 547)

(c) Following a), there were some primitives that were rarely used in recombination tasks. For example, a circle is very scarcely drawn as a single circle primitive for subject 1 (as shown in Fig. 2l, 4 out of 5

circles were drawn as a combination of other primitives, and also in Fig. 2o, the matches for the circle are very few and of relatively low quality). However, in Fig. 2g, the circle was shown as an example for categorical structure. It is confusing whether the circle primitive was actually learned by the macaques. The authors could include behaviors across all the different tasks to justify the acquirement of each primitive since incongruency will undermine their conclusion.

We take this criticism to be related to the previous comment 1a: that subjects should exhibit a uniform distribution over primitive frequencies, and they should use primitives matching the shape components in each image. For the reasons we detailed in the response to that comment, we do not think these two concerns are evidence against the claim that subjects learned and recombined these primitives. As discussed more extensively above, first, non-uniformity is consistent with behavioral findings from other behaviors with discrete units (e.g., language) and may reflect adaptive constraints for information compression; second, character images were ambiguous and therefore subjects were not required to match shape components to stroke primitives one-to-one.

We also address the specific example given by the reviewer, which focuses on the “circle” as evidence for a primitive that was not acquired by the subject, based on the relatively lower frequency with which character strokes were labeled as “circles” in the example trials in Fig. 2l and in the across-trial distribution in Fig. 2o.

First, we comment on the observation that the subject sometimes (but not always) used the circle primitive when the circle shape was embedded in the image. We do not think this is evidence against the subject acquiring the circle primitive, because the subject used the circle many times (n = 144) in the dataset (Fig. 2o), and each of these 144 cases passed stringent criteria to be confidently labeled “circle” (see examples in Fig. 2n, and Methods).

As an aside, we think the reviewer's observation is interesting in pointing out that the subject found alternative ways to draw circle shape components. Across the five examples with circles (Fig. 2l), the subject parsed circles using the following primitive combinations: squiggle + line, circle, squiggle + arc, arc + line, or squiggle. The constraints that drive particular preferences for one set of primitives over another, perhaps reflecting efficiency or other constraints, would be an interesting topic for future study.

[redacted]

[Redaction] Includes Third Party Material

Second, as alluded to above, even in human writing systems, some elements are used much less frequently than others. This table shows the usage frequency for each English letter (Wikipedia). Many

letters, including “z”, “q”, “j”, and “x” are used as infrequently as the “circle” was used by our monkeys, but there is no doubt that people understand these letters as distinct visual-motor concepts.

Together, these arguments support our conclusion that the “circle” primitive (and by extension, each other primitive) was successfully learned.

(d) Following c), the sigmoidal “category boundary” results regarding between-shape morph sets are encouraging, but they are not necessarily proof of the usage of independent action symbols; training-induced motor output bias towards learned movements (being more efficient to perform and to thereby obtain a categorical-score-dependent reward, thus constraining degree of freedom) might also explain the data. What behavioral/neural evidence is (or could be) there to support the notion that these animals were using symbols to perform each task, and not simply getting more skillful at generating motor responses de novo?

We thank the reviewer for raising the question of how the finding of categorical structure speaks to the presence of action symbols. Our main argument is that categorical structure indicates that primitives are represented with discrete structure. This is relevant for making a case that primitives reflect symbolic representations, because a hallmark of symbolic representations is their discrete structure.

We also address the possibility that the reviewer’s statement that there exists “a categorical-score-dependent reward” implies that we rewarded the subject for making strokes that looked more like primitive category 1 or 2 in the “categorical morph” tasks. We did not do this. Instead, the score for the morphed images was based on matching its image, and therefore would actually be *lower*, not higher, if the subject drew in a way that matched primitive category 1 or 2 (as they did).

We also address the possibility that the reviewer’s main concern is that the extensive training on primitives trivially explains the categorical structure, because subjects might just be reproducing rote memorized movements (“training-induced motor output bias towards learned movements”). However, we know that the subjects are not just producing rote memorized movements, because they behave flexibly and compositionally across the task variants. Subjects generalized to produce different movements at different sizes and locations (motor invariance), and composed primitives into novel movement sequences to draw characters (recombination). The presence of these three properties in combination (invariance, categorical structure, and recombination) indicates that the bias towards using primitives in the “categorical morph” tasks reflects a discrete internal representation, and not a rigid low-level motor habit.

To strengthen this argument that primitives representations are indeed abstract symbols that are not directly encoding low-level motor kinematics, we have performed additional analysis of PMv activity:

- We added results showing that PMv activity does not encode generic kinematic variables, whereas M1 activity does (**Extended Data Fig. 16**).
- We added a paragraph to the Discussion summarizing our findings that PMv activity is not tied to motor output, starting “Several lines of evidence indicate that this action symbol representation in PMv is abstract, not directly driven by visual features or motor parameters.” (lines 722 to 735).

We also thank the reviewer for proposing an alternative to action symbols, “*getting more skillful at generating motor responses de novo*”. However, we believe that this statement is not detailed enough

to be considered an alternative *mechanism,* but it instead specifies the *behavioral observation* that our study works to explain. In other words, it raises the question: “what mechanisms enable us to become skillful at generating novel goal-directed motor behavior?” Our study provides an answer: these mechanisms involve acquired internal representations of action symbols that can be flexibly recombined to generate novel, goal-directed, motor responses.

(e) In addition, the equivalent to Fig. 2o for subject 2 appears absent.

We thank the reviewer for pointing out that omission. We have now included both subjects in Fig. 2o. We note that both subjects show the behavioral characteristics in question.

2. My second concern is, supposing the macaques acquired the symbol system, whether the recorded neurons support such symbol representations. The electrophysiological recordings covered multiple cortical regions, among which only PMv showed potential symbolic encoding. However, as the authors already stated in the Discussion, PMv encodes invariant action representations (Taghizadeh et al., Nature Communication, 2024; Harpaz et al., Neuron, 2014). The results could be explained as an egocentric encoding of action sequences in PMv. The authors emphasized that the differences between action symbols and invariant action representations are categorical structure and recombination. However, since the macaques were eventually led to perform action sequences in these tasks, it’s also possible that PMv was just encoding the actions as the output of a symbol system, rather than the symbol system per se. In other words, the authors didn’t decorrelate the symbol system with the action output it produces.

We thank the reviewer for the many thoughtful points in this comment. We break down the comment into its three main points.

Reviewer point #1
The reviewer’s first point is that PMv may not be unique in its invariance. This is implied to us by the reference to Taghizadeh et al., 2024, a study which reports size and location invariance in PMd. Note that they did not test PMv. They identified this invariance in tasks requiring reaches targeted to specific parts of virtual objects, with objects varying in size and location across trials. This finding may seem to suggest that PMd exhibits similar location and size invariance to PMv. But this likely reflects a different phenomenon, for the following two reasons.

First, we have direct evidence comparing PMv and PMd in our task, and found neither location nor size invariance in PMd, while we found both in PMv (location: **Fig. 4;** size: **Extended Data Fig. 10**).

Second, Taghizadeh et al. report that location invariance in PMd was present only during a “visual working memory” trial epoch, when the stimulus disappeared and the subject had to remember a cued object and within-object reach location. During the subsequent planning epoch, when the object became visible and the subject could plan the reaching movement, PMd stopped being invariant to location. (Whether size invariance also disappeared is unclear, as they did not test this). This finding of location-dependence in PMd during planning is compatible with our finding of location-dependence in PMd during a planning epoch when the target was visible. This likely reflects PMd’s role in planning spatially-directed reaches. One interpretation (suggested by the authors) is that invariance in PMd during the working memory epoch is more related to an explicit strategy for encoding locations in an object-centered reference frame (e.g., left-most, middle, ...) or an abstracted positional code (1st, 2nd,

3rd,.. position on the object). This explicit encoding would be an interesting form of abstraction, but one of spatial location, not one of action symbols.

Indeed, lack of invariant encoding of movement in PMd is consistent with prior literature. While there have been some findings of invariance in motor cortex (e.g., in M1: Harpaz et al., Neuron 2014; Harpaz et al., CerCortex 2019, Hatsopoulos et al., JNeurosci. 2007), outside of PMv, invariance in dorsal premotor and motor cortex is uncommon, with most studies finding encoding that reflects a mixture of motor parameters, such as posture, location, and reach angle (e.g., Kakei et al. NatNeuro 2001 showing posture-invariance in PMv but not in M1; Taghizadeh NatComm 2024 showing lack of location-invariance in PMd during reaching; and Caminiti et al., JNeurosci 1991 showing lack of location-invariance in PMd and M1).

In summary, both our results and the literature suggest that the invariance we observed in PMv is different from invariances previously reported in PMd.

To strengthen the above argument that PMv activity is invariant to location, while PMd's activity is not, we performed additional analyses showing that location invariance is present during the reaching movement from the finger "hold button" to the starting location of the stroke. This complements the analysis in the initial submission, which focused on the planning epoch. This adds further evidence that PMv differs from PMd as described in the Taghizadeh et al. study (which found that PMd switches to losing its invariance as the animal prepares to reach) and the broader literature.

- This new figure is in **Extended Data Fig. 9.**
- It is incorporated in the main text in the section starting (lines 455 to 457): "*First, we tested whether location invariance was present also during the initial reaching movement between the go cue and the start of drawing (Extended Data Fig. 9a)....*"

Review point #2

The reviewer's second point is that invariance in PMv is to be expected based on prior recording studies, which diminishes the impact of our finding ("However, as the authors already stated in the Discussion, PMv encodes invariant action representations"). We have three responses.

First, and most important, we emphasize the key logic of the argument supporting our conclusion of discovery of an action symbol representation in PMv. Our criteria for concluding that any area (it just so happened to be PMv) encodes action symbols is based on all three properties of invariance, categorical structure, and recombination being strongly present in neural activity. We tested each of these three properties separately, and strikingly, each property was strongest in PMv. The importance of this convergence of properties is both conceptual—they are essential for a symbolic representation and thus they must be found in the same population for us to conclude that the population encodes action symbols—and (to a lesser extent) statistical—each of these three symbol properties was present to a greater extent in PMv than in every other recorded area, which is unlikely if these three properties were independent. Regarding this last point, note that we recorded many areas (8), which is significantly more than is commonly done in individual macaques (~1-4). We reiterate that, even though there are prior studies finding invariance in PMv, no prior study has reported a single population encoding these three properties, and therefore our study is the first to directly address the neural basis of action symbols.

- This is a point we previously made in the Discussion (currently lines 745 to 749): "There are differences between these proposals and the action symbols framework, both

conceptually—action symbols place stronger emphasis on categorical structure and recombination—and experimentally—we tested both categorical structure (in a systematic manner not previously done) and recombination into novel sequences (differing from previous studies, which behaviorally tested only a few well-practiced sequences)."

- We added further emphasis of the point that symbolic representations require finding not just invariance, but invariance in combination with categorical structure and recombination. We added this line to the Discussion: "Our study's criteria of invariance, categorical structure, and recombination may be useful in future studies of symbolic neural representations, including in reassessing prior findings of invariant activity in cognitive tasks." (lines 716 to 719).

Second, the fact that there already exists evidence for invariance in PMv is useful in suggesting broader implications of action symbols. Specifically, action symbols may be a framework for explaining some prior findings. For example, mirror neuron activity reported in PMv may reflect activation of an internal symbolic representation of the action in question, which may contribute to functions including planning, action recognition, and so on.

- We noted this point in Discussion: "Our findings raise the question of whether PMv's involvement in diverse motor behaviors—including speech—may be explained by the single principle that PMv encodes action symbols, a question which may be addressed by testing for symbolic properties, abstraction, and involvement in compositional generalization" (lines 750 to 754)

Third, the literature does not universally show that PMv exhibits invariance. For example, the mentioned Harpaz (Neuron 2014) study suggests that PMv activity is not invariant to size (and in fact, they found evidence for invariance in M1), in a handwriting task humans performed in an fMRI scanner. Another example is in a study of grasping in macaques, in which PMv activity encodes not only the grasp type, but also the object size (e.g., Schaffelhofer & Scherberger, eLife 2016). Therefore, because there is evidence for both invariance and variance in PMv activity, our findings of location and size invariance in PMv were not entirely predicted, and therefore are informative to the kinds of invariances that exist in PMv.

Reviewer point #3

The reviewer's third point is that maybe "*PMv was just encoding the actions as the output of a symbol system, rather than the symbol system per se*". We think this is unlikely, for a few reasons. Importantly, PMv invariance was during the planning period and not directly related to motor output (in many cases more than a second before movement onset). Relatedly, other areas known to encode motor output, including M1, PMd, and SMA, did not exhibit the same invariant properties as in PMv. Moreover, we did not find action-symbol encoding in prefrontal executive areas that may be expected to have such encoding, including vlPFC, dlPFC, and preSMA [e.g., prior evidence for perceptual categories in vlPFC (Freedman Science 2001), action categories in dlPFC (Shima Nature 2007), and abstract sequence representations in preSMA (Tanji Nature 1994)]. In summary, the fact that we found this representation specifically in PMv, not directly tied to movements, even with recording from many other motor and cognitive areas, indicates that this is not PMv simply encoding motor output.

To further bolster this argument that PMv's encoding of primitives is abstract and distinct from the encoding of motor output seen in PMd and M1, we performed a two additional analyses:

- First, during the initial reaching movement from the fixation button to the drawing, we found that PMv still exhibits location invariance. In contrast, M1, PMd and SMA strongly encode location, compared to PMv. See new figure in **Extended Data Fig. 9**.
- Second, we performed an analysis showing that PMv activity does not encode generic kinematic variables (that generalize across primitives), whereas M1 does (**Extended Data Fig. 16**).
    - This result is part of the new Results section: "PMV encodes primitives in a manner dissociated from both the shape in view and from motor kinematics". (lines 638 to 666).
- We added a paragraph to the Discussion summarizing our findings that PMv activity is not tied to motor output. See paragraph starting "Several lines of evidence indicate that this action symbol representation in PMv is abstract, not directly driven by visual features or motor parameters." (lines 722 to 735).

Minor points

We note, for the sake of completion, that we interpreted the reviewer's comment "The results could be explained as an egocentric encoding of action sequences in PMv" to mean either that (i) it is already known that PMv encodes invariant representations of action (in which case our response is under point #2), or (ii) that PMv is just encoding actions as the motor output of a symbol system (in which case our response is under point #3).

As a minor revision, we have added citations to prior literature supporting our choice of location and size parameters, as values which are large enough to drive variation in motor cortical and muscle EMG activity in prior reaching studies:

- In Results: "using location and size parameters expected to elicit variation in both motor cortical and muscle electromyographic activity based on prior studies of reaching movements; see Methods" (lines 186 to 187).

3. My third concern is with insufficient analysis of symbol recombination. As suggested by the authors' previous paper (Science, 2015), recombination involves the structured organization of discrete symbols through specific relational rules (e.g., directional or sequential relationships). The current manuscript lacks exploration of such rules at both behavioral and neuronal levels, weakening the authors' claims for evidence of recombination.

We thank the reviewer for this insightful question that links to our prior work on symbolic representations in handwriting (Science, 2015). The reviewer is referencing that paper's conclusion that people have both an internal representation of stroke primitives (action symbols) and can apply rules that define how symbols should be combined (syntax). However, when it comes to the study of neural substrates (as in the current study), we think that these two questions of how symbols are *represented* and how symbols are *used* are separable. Indeed, this separability is consistent with the computational model for handwriting in the 2015 paper, which had distinct "modules" for symbols and syntactic rules. This may be seen as predicting the presence of distinct areas/populations in the brain for symbols and syntax. In that vein, the current study is addressing one question: how symbols are represented in the brain. We believe that we have performed the essential experiments to show that stroke symbols are represented in PMv.

At the same time, we agree that asking what mechanisms implement rules that combine symbols is important for future studies, and can be addressed both in terms of how images are parsed into

symbols, and the mechanisms for organizing symbols into motor sequences. These are significant projects that would require new and extensive analyses and experiments, and which would go beyond the scope of this paper. Importantly, these future studies could build on the finding here, of a representation of symbols in PMv.

- We raise the question of rules/syntax in the last paragraph of Discussion, both to place the finding of action symbols in a broader context, and to suggest future research directions: "Looking forward, an important aspect of finding an action-symbol-encoding population in PMv is that it could be a foundation for future studies to test for activity dynamics consistent with symbolic operations in programs…" (lines 823 to 825).

Note that our response here mirrors a point made by Reviewer 3 that our scope is appropriate: *"The strength of the manuscript is that authors focus on a very targeted part of the problem - the representation of action symbols — and provide extremely strong empirical evidence for these particular claims. They describe this evidence very thoroughly, leaving little room for doubt."*

At the behavioral level, with more than a dozen "primitives", the learned stroke patterns are perhaps too exhaustive for alternative draw paths to appear; that is, whatever motion on the touchscreen a monkey makes, it might become significantly similar enough to one of the "primitives" regardless of the monkeys' intention. The current task paradigm therefore may not be able to offer a suitable control against possible alternative hypotheses in Fig. 2k and beyond. Perhaps seeing what monkeys would do if trained to reproduce complex traces without previous training on "primitives" could help?

The reviewer raises an important question of whether the finding of recombination on character tasks is trivially due to character strokes "accidentally" matching one of the primitives, such that, even if a monkey were to scribble in a mindless way not related to his primitives, those scribbles would still tend to match his primitives at the high rate that we found (~80%). Our results (both old and new) show that this is not the case.

First, in the "accidental matching" situation, we would expect both subjects' strokes to match both subjects' primitives at similar frequencies. This is because, if subjects were simply "scribbling" and not biased to use their prior distribution of primitives, they would be expected to produce unstructured stroke distributions similar to each other (because they were tested on identical images). We would expect such similar distributions to match both sets of primitives at similar rates between subjects. Instead, we found that each subject's strokes matched its own primitives, and not the other subject's primitives (Fig. 2p). This is the first evidence against "accidental matching."

Second, we performed an additional control analysis that does not rely on comparing across subjects. We compared each subject to a null model based on generating new simulated primitives with similar "subpart" structure to the real primitives, but different idiosyncratic "whole" structure. We did this by "mixing" the actual primitives, connecting the first half of one to the second half of another. We found that this led to a poor match between strokes and these remixed primitives. This argues that subjects were really reusing their idiosyncratic primitives.

- These results are in an updated **Fig. 2p**, and the remixed primitives are shown in **Extended Data Fig. 5**.
- We have described this in a new section in Results, starting with: "Second, we asked how often strokes matched simulated primitives designed to resemble the actual primitives in their subparts (i.e., their first and second halves) but not in their entire trajectories (see simulated

primitives in Extended Data Fig. 5). We found that strokes infrequently matched these “remixed” primitives (<43% for novel characters, Fig. 2p).” (lines 349 to 353)

We agree that it would be an interesting exploratory study to test what monkeys would do if trained to reproduce complex traces without previous training on primitives. We would gain insight into subjects' innate biases, including the possibility of there being innate primitive movements, or of them learning low-level tracing strategies that lack stroke primitives. This experiment would be extremely demanding, as it would require training separate groups of monkeys from scratch on multiple task variants (with and without trained primitives). Given our rebuttal points above, we think this is not necessary in the current study.

At the neuronal level, the manuscript does not investigate how relationships between primitives (e.g., their order, spatial arrangement, or hierarchy) are neurally encoded during novel pattern reproduction. For instance, if recombination involves combining primitives via relational rules (e.g., “up-left-down”), neural activity may reflect the primitives and the transitions/rules linking them.

In summary, recombination requires evidence of structured relationships between primitives, not just their isolated usage. The current analyses do not adequately address this, and additional experiments or re-analysis focusing on relational dynamics would be needed to substantiate the central hypothesis.

The reviewer suggests experiments that elucidate the neural implementation of rules that act on symbols, and/or the neural encoding of relations between primitives. We acknowledge the importance of these questions towards gaining a complete understanding of how symbols support compositionality. However, the focus of the current paper is on one specific question: the representation of action symbols. Our analyses and experiments were specifically designed to test for a symbolic representation in behavior and neural activity, and to that end, we believe we have presented thorough supporting evidence.

The question of how rules are implemented is a formidable question that is beyond the scope of the current paper. It deserves a thorough study on its own, which is ideal for follow-up studies that build on this study's finding in PMv.

We also wish to clarify the goals of our analysis testing for activity reflecting recombination. Reiterating our point above, the goal was not to elucidate the neural activity or mechanisms that perform this recombination (a goal for future research), but was to test whether primitive representations (including in PMv) are reused in the context of recombination, which we succeeded in doing. This is an essential property of action symbols.

Minor issues:
1. Fig. 2o could perhaps be normalized into a high/low-quality match ratio to accommodate different numbers of total matches per category.

We realize, from this comment, that Fig. 2o was a bit confusing, because, by presenting splits of each primitive into high- and low-quality matches, it implied that there was something meaningful about the primitive each low-quality match was assigned to. Instead, all strokes that fail to have a quality match fall into a single bucket of strokes that do not match any primitives (“no match”). This is the approach we originally took in the summary plot (Fig. 2p).

- We have updated Fig. 2o to just show the frequency distribution over primitives. Note that we have not lost any useful information, as the fraction of strokes that are high-quality matches is (as was previously) depicted in Fig. 2p.

2. What symbols are used in Fig. 2d and Fig. 4, exactly? Showing the stroke patterns would help.

We agree, and have added icons of the primitives to all figures that refer to primitive classes: Figs. 2d, 4, 5.

3. There seems to be some disassociation between behavioral and neural data analyses; for instance, are the "primitives" best learned by subject 1 (as seen in, say, Fig. 2o) also the ones best represented by neural activity? Are neural responses to the same "primitive" stimulus similar or distinct across monkeys like the behavioral outputs in Fig. 1e?

We appreciate this suggestion, but it is not clear to us how we would go about doing this.

First, there isn't an obvious measure that ranks how well each primitive has been learned. The reviewer suggests that this is indicated by the frequency of reuse in character tasks, but as we note above (response to comment 1a), we do not think that frequency is a good proxy for how well a primitive has been learned.

Second, it is not clear what would be an indication of "better representation" in neural activity. One possibility would be to use the Euclidean distances between primitive representations, but we do not believe that if a primitive is further apart from others, then this is an indication that it is better represented (see response to comment 1b).

Third, it is not clear how to compare neural activity across animals. As opposed to comparing behavior, which has the common reference frame of touchscreen coordinates, there is no common reference frame of neuron identities across animals. This is why all of our neural analyses focus on comparing different conditions within the same subject and within the same recording session, when we are confident about neural data having the same reference frame (i.e., defined by the set of recorded neurons).

4. Notably, the neural data on recombination came from -0.6 to 0.2s around the first stroke start (Fig. 6b) instead of the planning period around image onset used in previous analyses. The peri-stroke-onset time window might provide data that could be too complex to sort through (mixture of motor signals from hand rising to touch screen, motor planning, "Go" cue comprehension, etc.).

The reviewer raises an important point that the peri-stroke-onset time window may be too noisy or complex for these analyses. However, we think our results make it clear that this window was appropriate. We found strong primitive encoding in PMv, in spite of these potential mixed signals. It is also important to clarify that the window was before stroke onset (-0.5 to -0.05 s relative to stroke onset; see Methods), not overlapping the stroke onset, and so it's less of a concern that there was contamination by signals related to online control of movement.

- We have also updated the figure legends (Fig. 6e) with the time window used for analyses: "averaged over time (-0.5 to -0.05 s relative to stroke onset)" (line 624).

Overall summary of responses to Reviewer 4

In summary, Reviewer 4's main concerns were:

(1) Subjects did not learn and compositionally generalize the primitives.
(2) The finding of motor invariance in PMv is not novel enough to act as evidence for the novel construct of "action symbols".
(3) The finding of categorical structure is irrelevant with respect to testing for action symbols.
(4) The evidence for PMv activity reflecting recombination is not sufficient, because we also need to explain how the rules for combining symbols are implemented in neural activity.

We believe we have presented strong arguments against these points above. Here, we briefly summarize the essential aspects of our arguments:

(1) Evidence that subjects indeed learned the primitives comes from their strong capacity to spontaneously generalize in a compositional manner to draw novel images, across a diversity of task paradigms (testing invariance, categorical structure, and recombination). These behaviors cannot be explained simply by the effects of motor practice on primitives because they are on novel images.
(2) We emphasize that *the critical evidence* for PMv encoding action symbols is the finding of convergence in PMv of three essential symbol-encoding properties (invariance, categorical structure, and recombination). Importantly, this was done using an unbiased "screen" of multiple frontal cortical areas.
(3) The finding of categorical structure is important because it gives insight into the nature of the internal representation of primitives, which is relevant for making a case that they are symbolic representations, because a hallmark of symbolic representations is their discrete structure. This hallmark is stated in the large prior literature on symbolic representations that we cited in the Introduction.
(4) The critical evidence supporting that PMv activity reflects recombination is that PMv activity is *reused* in compositional generalization. The additional question regarding the neural mechanisms of relational and syntactic rules that act to "manipulate" these symbols is beyond the scope of our current study.

[redacted]

[redacted]

[redacted]

[redacted]

Referees' comments:

Referee #1 (Remarks to the Author):

We thank the authors for their detailed responses to our comments and to the substantial edits, both in terms of clarifications in the manuscript text and follow-up analyses that help bring greater clarity to the key questions at hand.

The edits to the Introduction and Discussion sections in particular help motivate, clarify and address several points raised in the initial review. The additional references added to the Discussion section also help bridge the current work to the existing literature (e.g., relating to hippocampal replay).

The new experiments assessing the (in)ability to decode visual features of the cueing stimulus, indexed by which part of the stimulus the subject is attending to during planning, relative to motor plans helps support the argument that pMV is primarily involved in planning action sequences and is not directly driven by the visual input being attended to. This helps distinguish the extent to which pMV engages in processing visual information vs. motor planning and addresses comments raised in the initial review. The follow-up analyses investigating the state-space trajectories of different primitives, both when drawn in isolation or as a part of composite characters as the first or second+ stroke, help highlight that there does appear to be reuse of same primitive-specific neural code when primitives are combined, addressing our initial comments regarding the lack of neural evidence for the same.

We thank the reviewers for acknowledging the improvements we have made to the manuscript.

Furthermore, we appreciate the extensive comments provided in response to the thought experiments we posited in our original review to more deeply understand the notion of "action symbols" and "compositional generalization" that are so central to this work.

As we understand it based on the response letter, in the authors' view, it is not critical that the action symbols result in visual marks, so long as they are internally constructed and goal-directed. However, it does seem important for an action symbol to be cued in such a manner that "surface-level" stimulus information is not sufficient to determine the behavioral output, and that instead an "internal constructive process" is needed. Indeed, these are the reasons given for why using arbitrary sequences of shapes/sounds (such as a red/green cue) to cue the production of actions—even if these actions possess all the hallmarks of action symbols—would not be sufficient to elicit genuine action symbols. Based on our understanding of lines 777-783 in the Discussion, ambiguity in the mapping between the sensory input and the motor output is argued to be critical for engaging true action symbols and for investigating true compositional generalization.

The reviewers raise an important point that warrants further clarification. We now realize a lack in precision of our previous response regarding the importance of stimulus ambiguity in compositional generalization. We did not intend to mean that action symbols are *only* induced in situations with ambiguity. Instead, we *agree* with the reviewer that even in situations with no ambiguity but with internally constructed and goal-directed movements, action symbols may very well be involved.

The point we intended to make was that ambiguity is a useful feature in tasks testing the existence, nature, and neural encoding of action symbols in compositional generalization. This is because ambiguity allows experimenters to probe the nature of prior knowledge while imposing minimal instruction from the environment. If subjects still exhibit invariance, categorical structure, and recombination (consistent across a large variety of stimuli), then this provides useful evidence for action symbols.

We regret causing this confusion, and hope we have made our position clear. We have revised the Discussion to clarify this point:

- In the section the reviewer references (old lines 777-783), we have now reorganized the paragraph to make clear that: "Second, images were generally ambiguous, which allowed us to probe prior knowledge." (lines 619 to 620).

However, there remain importantly puzzling aspects of these criteria that, to our minds, remain unresolved.

If it is the case that such ambiguity in how to parse the sensory input is a critical feature of an action symbol / compositional generalization, that would suggest that a key computational challenge is the "input parsing problem" and not only the action representation. The "input parsing problem" (so to speak) in the case of these image-cued drawing tasks is the problem of visually decomposing the target character. Without the challenge posed by the "input parsing problem," it would seem to be the case that the "GREEN RED" cue example (given in author response) ought to qualify, especially if that specific sequence of cues had never been seen or acted upon by the animal before. So either the "GREEN RED" cue example should be admissible, or it should be acknowledged that the construct under investigation is one that relies crucially on the ability to parse ambiguous (visual) inputs, and not so much the ability to compose previously learned action primitives in novel ways (if it were, then the "GREEN RED" example ought to be admissible). But if "the ability to compose previously learned action primitives in novel ways" is not the core phenomenon under investigation, then it is much less clear why "compositional generalization" is an appropriate term to apply to it.

The reviewers raise a logical follow-up to the previous question, that if ambiguity were essential to compositional generalization, then the “input parsing” problem would be fundamental to understanding its neural mechanisms. In the previous response, we clarified that stimulus ambiguity is a useful task feature, but not fundamental. As a consequence, we also do not view input parsing as fundamental to explaining action symbols and compositional generalization. A further consequence is that we do agree with the reviewer that the “GREEN RED” cue example may very well involve action symbols. Indeed, we tried to make this point in the previous Response: “In such a task [involving red/green cues], one might also expect action symbols.”

We hope this clarifies our stance, and importantly, that we have been sufficiently clear in the manuscript that the question we are addressing is “the ability to compose previously learned action primitives in novel ways”.

We do recognize that the input parsing problem is important for understanding the full breadth of how compositional generalization contributes to real-world problem-solving, and that it would be an interesting topic for future study, which may build on our findings in PMv.

Supposing a task that poses a challenging "input parsing problem" is actually an essential ingredient for studying compositional generalization (which I take the authors' response to have insisted upon, even if we remain unconvinced), then something else that remains unclear to us is what role Pmv is thought to play in the "input parsing" operations themselves and not just representation of the cued actions,which are visual operations in the context of the Character task. Clarification on this front is particularly important in light of the new experimental results in Extended Data 14., showing limited decodability of visual gaze patterns from Pmv. If Pmv is thought to play a crucial role, what direct evidence is there that these input parsing operations are performed in that region? If Pmv is not thought to play a crucial role, then what does that imply about the relationship between Pmv and action symbols / compositional generalization?

This comment continues the line of reasoning based on the possibility that we believe that the input parsing problem is essential to the mechanisms of compositional generalization. As we hope we have made clear in our responses to the previous two comments, we do not believe that it is essential. We thank the reviewer for initiating this deep discussion that allows us to clarify this point.

We were puzzled by the response to our thought experiment concerning the sequences of actions needed to open various food containers. While we were reassured that such scenarios might also hypothetically involve action symbols and support genuine compositional generalization, we did not understand why this setting was inherently less suitable for studying these phenomena than a drawing task. And we thought it possible that the "food container" example we gave might have been misunderstood. In this scenario, really meant to be a stand-in for a "physical problem-solving" or "tool-use" variant of the task posing the very same set of computational challenges, we do not really anticipate that the resulting movements produced by a participant would actually be "discrete" because they would be interacting with real physical containers. And certainly the way a food jar looks does not directly "instruct" a participant when it comes to how to open it. Suppose there was a tab that needed to be pulled, or a flip top, or a screw top, or a cloth covering. Food jars with these different lids look even less like the actions needed to open them than the letter "G" looks like the action needed to reproduce that letter by hand (cf. observation about isomorphism between visual input & motor output in the Character task), even if the kinds of actions needed to interact with each of the possible lids (e.g., screw

top, flip top, cloth covering) are themselves reasonable candidates for representation as "action symbols." Moreover, there are many such physical mechanisms that can be devised and learned, much as there are many characters that can be devised and drawn/written. Though it might simply be more expedient given the state of digital tablet technologies to use a drawing task rather than devising many physical puzzles. Is that right?

We thank the reviewer for clarifying the details of how this hypothetical task would work. From this, we agree that it would be an interesting paradigm well-suited to studying action symbols and compositional generalization, and moreover, in the relatively naturalistic setting of physical problem solving. Our initial misunderstanding was in thinking that the task would involve a series of discrete actions cued by objects one-by-one, with specific highly-practiced responses to each cue. But, instead, the reviewers clarified that this would be a self-directed task with rich variation in stimuli and action possibilities. We would be excited if future studies build on our findings in the drawing task to investigate these different settings.

On a separate note, while the new 'remixed' stroke analyses (Figure 2p.) are fascinating and do help contribute to the narrative that primitives are subject-specific and re-used, it was unclear how the strokes were remixed in the main results text and figure. While the remixing algorithm is described in both the methods and extended data, it would be helpful to at least note that primitives were remixed within-subjects earlier in the text, as this helps contextualize the results.

Thank you for noticing this ambiguity and pointing it out to us. We have added this clarification to the main text (addition in bold):

- "Second, we asked how often strokes matched simulated "remixed" primitives designed to resemble **one's own** primitives in their subparts (i.e., their first and second halves) but not in their entire trajectories." (lines 292 to 294).

Additionally, the current takeaway for this result is presented on L.353, "Combined, these control analyses show that character strokes specifically resemble one's own primitives (first analysis) in their idiosyncratic stroke trajectories (second analysis)."
What are the consequences of a subject's primitives (action symbols) being so highly specified that they only match a subject's own (the highly trained) idiosyncrasies? Greater discussion of this finding is warranted.

We agree with the reviewers that this is a notable aspect of the behavior. For us, there are two important implications. First, it gave us a useful signature of each subject's "style", which allowed us to look for reuse of this style as a behavioral signature of compositional generalization. Second, our study suggests that such idiosyncrasy may be a hallmark of action symbols, especially those that are learned with strong environmental influences. We have revised the Discussion with this point:

- "Related studies have identified reused segments (or "syllables") in minimally-restrained behaviors, including in birdsong (**and in some cases syllables can be idiosyncratic to individuals, similar to stroke primitives in our study**)." (lines 639 to 641)

The authors have clarified their stance on the importance of constraints on naturalistic skill learning (handwriting, musicianship, athletics, etc.) and have highlighted that even if primitives are learned, the core contribution in the present work is evidence of how these primitives can be generalized, which we agree is not a trivial feat. Nevertheless, it would be helpful to comment on the importance of how the

action primitives were induced in the present study and how that might relate to how action symbols might emerge in naturalistic behavior. Would the decodability of signals from pMV have been noisier if the learning of primitives were less strictly enforced (noting that the authors did allow for some flexibility in how primitives were learned – e.g., results from Figure 2p.)? In a non-drawing task, where there is even looser isomorphism between cued stimuli and desired actions, would more training/learning be needed to arrive at action symbols?

The reviewers raise an interesting question related to understanding the learning and innate constraints that support the emergence of action symbols across different naturalistic behaviors. Answering this question is difficult given current uncertainty about how action symbols contribute to other behaviors. We speculate that they may be involved in a variety of behaviors, including ones at different points along the axes of innate vs. learned (as suggested in Discussion). If so, we may realize that there is a lot of variability in the relative contributions of learning and innate knowledge in action symbols, and how these factors interact during development and learning. Stronger environmental and training constraints may lead to action symbols that are more stereotyped and, given sufficient variability across individuals, more idiosyncratic. We hope that future work may eventually support such a comparative study of action symbols across naturalistic behaviors.

Thank you again for your thoughtful response and we look forward to hearing what you think after having reviewed the above comments.

Referee #2 (Remarks to the Author):

I co-reviewed this manuscript with one of the reviewers who provided the listed reports.

Referee #3 (Remarks to the Author):

The strengths and weaknesses of the article are not really changed from the last version, but the article has nevertheless improved. The article should now be published (subject to one very minor change below that does not require re-review), and the authors commended for their attention to the comments and commitment to open science.

We thank the reviewer for this positive assessment.

Notable changes:

- *They now provide source code and data. My lab wrote small scripts of our own to plot neural activity and behavioural activity, which wasn't too hard, and as far as we tested, all of their code works and produces the paper's figures.*
- *They added reasonable analyses related to (i) where the animal is looking, to show that the visual content is what drives the PMv activity, and (ii) encoding analysis from velocity straight to neural data, which shows that while it explains some of the M1 activity, it doesn't explain the PMv activity.*
- *They also provide useful analysis of neural activity of the middle- and late- strokes in multi-shapes trials, which require doing some correction for the fact that the first "shape" starts with a massive motor movement from the fixating "poke" to the shape onset.*
- *They provide rather unreadable new figure 13 (Minor comment: In my view, these scales are harmful for the message they are trying to convey; binarise, or give us real scales, the current version makes it impossible to know what is significant and what isn't)*

The reviewer makes a fair point that the scale makes it difficult to identify which comparisons are significant.

- Following his suggestion to "binarize" the scales, we have now updated the figure to use a unique color to mark comparisons which are not significant (Extended Data Fig. 13).

- *The discussion is more structured and now includes a section about ventral and dorsal pathways and the location of PMv in all of this, and another making it clear what they mean by their finding being an abstract "action symbol".*

Importantly, one of the major concerns that I had reading the other reviewers' comments has been cleared: they are _not_ penalised if they don't use sub-shapes (which, indeed, would have made the claim much weaker). This comes from a slightly confusing terminology between single shape, multiple shapes (several on screen, but disconnected; there it is penalised to use a single stroke since they are disconnected) and character (complex shape). Maybe they could find a better name or make the terminology clearer in fig. 1.

We are glad to hear that this point has been clarified. Following the reviewer's suggestion, to make this point even more clear, we have revised the text in this Results section to emphasize that subjects are not constrained to reuse their primitives to match the character subshapes.

- "We then tested subjects on complex figures called characters. Although characters were designed by connecting multiple simple shapes, subjects were not forced to draw using primitives that matched these shape components, and characters had ambiguity in being

consistent with multiple possible interpretations in terms of shape components." (lines 267 to 270)

The biggest drawback of the paper remains true: As pointed out by all reviewers this is not a paper about compositionally or how things combine or rules, etc.. However, they are careful not to claim that besides one opening in the end of the discussion. The data they do present are nevertheless remarkable.

We thank the reviewer for this comment, which we believe is fair.

Referee #3 (Remarks on code availability):

The code is available and can be used by a skilled postdoc :)

Referee #4 (Remarks to the Author):

We appreciate that the authors' point-by-point response is drafted with impressive thoroughness and outlines a greatly improved manuscript. There are still a few issues where my opinion diverges from that of the authors.

We thank the reviewer for this favorable assessment of our revisions.

I agree with the authors that, for the purpose of demonstrating that some primitives were learned and reused to draw complex figures, what's already in the manuscript should suffice. However, the comparison to language and other forms of acquired compression is not without question. Unlike these cases where the inherent structure of environmental inputs could lead to biased distributions, in the case of this study's single strokes, the training was presumably conducted without bias among all stroke types. If this is not so, then perhaps the main text should make it clear.

We agree with the reviewer's implied point that we should de-emphasize the importance of this comparison to language in coming to the conclusion that primitives were learned and generalized. Indeed, the main arguments in support of this conclusion are from *positive* evidence in their behavior (as elaborated in our previous round of responses). We are pleased that the reviewer now agrees with us that "for the purpose of demonstrating that some primitives were learned and reused to draw complex figures, what's already in the manuscript should suffice".

We do agree that it is difficult to make a one-to-one comparison between our paradigm and language, in which some of the bias in primitive use may indeed be explained by bias in the distribution of environmental inputs (e.g., distribution of words in books). The reviewer is correct to note that our training, in contrast, did not have such a bias. We have revised the main text to make this clear:

- "Note that the non-uniformity of this distribution is reminiscent of frequency distributions seen in language and other behaviors (**although those cases may in part reflect non-uniformity in "training" experience**)" (lines 204 to 206).

Another of my concerns was with whether one could indeed call what the animals learned a "basis set", given the tendency for some primitives to be rarely used and/or decomposed when drawn (which would reduce trace similarity and therefore reward). Wouldn't the monkeys be encouraged to use the most efficient primitive with the goal of reproduction in mind? I understand that the usage of primitives is not necessarily efficient in every case, but still it challenges parsimony in explaining behavior. That being said, within the authors' stated scope, it may already be sufficient to demonstrate that certain reusable primitives were sufficiently independent of each other. It might be still better to show directly that even rarely used and/or often "decomposed" primitives were nonetheless reliably reused when drawing complex figures.

We thank the reviewer for raising this discussion. A key idea for us is that our findings do not depend on subjects frequently using every single primitive. Having a bias to use some primitives over others does not detract from the main findings that show that the subject *does* reuse primitives and that this reuse reflects an underlying action symbol representation. We are thankful that the reviewer seems to agree with us: "within the authors' stated scope, it may already be sufficient to demonstrate that certain reusable primitives were sufficiently independent of each other".

We agree with the reviewer's suggestion to highlight that even low-frequency primitives were reliably reused. Our analyses already show this, because even primitives that were low frequency still passed conservative criteria for the identified strokes to be labeled as those primitives. We have updated the text to point this out:

- "Note that even low-frequency primitives still passed criteria to be deemed high-quality matches (Methods)." (lines 206 to 207).

The reviewer raises an interesting question about what notions of efficiency may account for the biases seen in subjects' choice of primitives. As the reviewer implies, we suspect this may reflect a combination of factors, including not only biases in what primitives a subject likes to use, but also motor efficiency and biomechanical constraints. Optimization of these and other constraints during planning may contribute to the process of parsing an image into an action plan. This would be an interesting topic for future studies.

Moreover, I would like to discuss the integrity of the putative action symbols. In handwriting, it is fine to write "d" as an "o" and an "l" connected together. Even if one does so, the semantic meaning of "d" would not be decomposed into "o" and "l", which I would call the integrity of meaning for a symbol. However, the semantic meaning of an action symbol is perfectly aligned with the process of producing it. In other words, the "meaning" of an action symbol is defined by how it is produced. This is part of what's causing my confusion when the subject parses a known primitive into several other primitives; does it also decompose the meaning of a primitive, or does it just decompose the production? If the former, when the subject draws a circle as a squiggle + line, there should be a compound representation of squiggle + line in neural activity. If it is the latter case however, the neural activity should represent a circle. I think it is worth diving into those cases more carefully.

We thank the reviewer for bringing up this important question: is the "meaning" of an action symbol related more to it being a representation of the shape image or of the action? We think action symbols relate to the action. We were in fact able to address this question directly in the neural data, focusing on situations where the image shape and the behavior were dissociated. We found that action symbol representations are related more to the planned action than the visual content, across a variety of analyses, which are summarized in a dedicated Discussion paragraph (lines 569 to 580).

Consistent with these and the other findings in our paper, we suggest that the "meaning" of an action symbol may be understood as a combination of (i) its role as a unit that can be composed with other units to construct goal-directed action sequences, and (ii) its "top-down" mapping to the corresponding motor output. We are not claiming that this meaning is the same kind of semantic or referential meaning attached to other kinds of symbols, which can be very abstract, such as symbols in language or mathematics. We are simply focusing on one fundamental aspect of "meaning", which is the role that a symbol takes within a computational system. Because of the somewhat speculative and philosophical nature of this point, we have not emphasized it in the paper - notwithstanding its importance.

Finally, we agree with the reviewer's implication that our work does not rule out that the subject may also have symbolic representations of shape parts in a more visual domain, and that such "perceptual symbols" could feed into or interact with action symbols. Testing this possibility would be an interesting topic for future work, especially using recordings in visual areas of the brain.

While I concur with the authors that a more comprehensive investigation of relational rules and their underlying neural mechanisms falls beyond the scope of this study and should be deferred to future work, I remain concerned that the current PMv data pertaining to "recombination" are somewhat limited in their interpretability.

Specifically, to robustly demonstrate recombination, it would seem necessary to present neural data that involves interaction between representations of at least two distinct strokes (e.g., by examining the bivalent parsing of complex shapes). Although Extended Data Figure 14 partially addresses this, the evidence remains insufficient. Moreover, the consistency of stroke representations across different ranks in complex symbol drawing, while informative, does not fully substantiate the claim of recombination. If recombination is occurring at the output stage of motor processing, one might expect contextual modulation to differentially affect the first and second strokes, thereby reflecting their distinct roles in the sequence. Currently, the PMv data appear to reflect the elementary components/"ingredients" (cf. the discrete stroke class z_ij in Lake et al., "Human-level concept learning through probabilistic program induction") of recombination, rather than the dynamic process itself. It may be worthwhile to re-examine the dynamics of potential contextual modulation within the related time window.

We are glad that the reviewer agrees that investigation of relational rules falls beyond the scope of this study.

We also agree with the reviewer that it is important to clarify how our findings relate to the dynamic process of recombination. Our main argument is that PMv represents the *components* that are recombined (indeed, similar to the discrete stroke classes in Lake et al). To do so, this required identifying PMv's representation as being reused in the context of behavior exhibiting recombination. To us, it is striking that PMv does *not* exhibit the kind of contextual modulation that the reviewer — rightfully — suggests as a hallmark of a neural process that implements the mechanisms of recombination. Indeed, going into the study, we thought it quite possible that PMv would have significant contextual modulation given its known function in higher-order aspects of action, and its direct connections with prefrontal cortex and other higher-order areas.

Given this lack of contextual modulation in PMv, the mechanisms of recombination likely depend on PMv interacting with other areas. As a hint of what that might look like, we point out one of our results, that in preSMA we found contextual modulation (**Fig. 6**). This suggests a critical role for preSMA in the dynamic process underlying recombination. A possible avenue for future work — on the mechanisms of rules and recombination — would be to assess the nature of that contextual modulation in preSMA and simultaneously recorded activity in PMv. We thank the reviewer for leading us towards this possibility.

- To note this possibility in the Discussion, we have made this revision (updates in bold):
- "Building on our results, future studies may test for activity consistent with symbolic programs in PMv and connected areas, including PFC and preSMA, **areas known to encode sequential context [in prior[36,37,57,95] and the current study (preSMA, Fig. 6)],** and in hippocampal circuits implicated in generating compositional representations." (lines 650 to 653).

Additionally, I suggest moving Extended Data Figure 14 to the main text, as Figure 6 (which focuses on recombination) is currently under-supported and would benefit from more direct evidence.

We think this is a useful suggestion and see the merits of doing so. However, as the Editor has reminded us, the manuscript is currently excessively long, and we feel that the Extended Data Figures are very similar to the main figure and also prominently described in the text. We therefore hope that the reviewer agrees with our decision to leave Fig. 6 as is, and suggest the Editor decide this.

Furthermore, the authors' proposed "recombination" is framed so far mainly as "reuse." I agree that simultaneously addressing action symbol representation and syntactic structure may be overly ambitious for a single paper, especially since the neural representation of action symbols itself has not yet been firmly established. Thus, focusing solely on action symbols is justifiable. However, I find that the novelty of the study is limited if it only demonstrates neural representation of action symbols with motor invariance, as this phenomenon has previously been observed in other motor areas. More abstract representation of movement—even across varying combinatorial contexts—is already a known capability of motor cortex and does not, in itself, constitute evidence of recombination. In this context, I recall the work by Zimnik and Churchland ("Independent generation of sequence elements by motor cortex"), which appears to preempt in PMd much of the conclusions presented here—especially if one considers a double reach task as also based on the elementary "action symbol", i.e. the reach movements, which in their work are also invariantly represented and can be recombined to form a variety of complex movements. While it is encouraging that only PMv shows all of the properties for action symbols in the current manuscript, the novelty is to some extent compromised, when the take-home message can be summarized as "reuse of invariantly represented movements", with no further analysis for the mechanism of recombination/relation, nor analysis of how exactly the primitives are represented in PMv. Perhaps the dynamic features in the planning period could be explored further, rather than simply having them described as distinct trajectories.

We appreciate the reviewer providing a summary assessment of the novelty and impact of the paper, and in particular, pointing out the importance of engaging with prior work in motor and premotor cortex on invariant encoding. Indeed, prior work has identified a variety of kinds of invariance, which we do cite and discuss in the manuscript (lines 582 to 594, Discussion) and in our previous response to reviewers. We do not wish to discount any of this prior work, which has been very relevant for placing our study in context. We thus appreciate this opportunity to extend this point.

We agree with the reviewer's statements that "*motor invariance … has previously been observed in other motor areas",* and that, "*More abstract representation of movement—even across varying combinatorial contexts—is already a known capability of motor cortex*." However, we believe our study is the first to demonstrate invariance and abstraction in the manner necessary for investigating action symbols. <u>The critical evidence</u> for PMv encoding action symbols we provide is the finding of *convergence* in PMv of three essential symbol-encoding abstract properties (invariance, categorical structure, and recombination).

It is also worth noting that prior studies have found evidence both for and against invariance (as we noted in our response to the previous reviews), meaning that our finding of invariance was not trivially expected. Importantly, we conducted an unbiased "screen" of multiple frontal cortical areas. For these reasons, we think that our study is the first to provide direct evidence for neural encoding of action symbols, and we respectfully disagree with the reviewer's suggestion that we "only demonstrate neural representation of action symbols with motor invariance".

Regarding the specific question of recombination, we agree with the reviewer that prior studies have found evidence for motor cortical activity being reused in different sequential contexts. Zimnik and Churchland is raised specifically: "*Zimnik and Churchland … appears to preempt in PMd much of the conclusions presented here—especially if one considers a double reach task as also based on the elementary "action symbol", i.e. the reach movements, which in their work are also invariantly represented and can be recombined to form a variety of complex movements."* While Zimnik and Churchland — and other studies finding reuse in motor cortex — are complementary and relevant, our findings in PMv are not preempted to their findings in PMd and M1, for a few reasons.

- First, their study does not systematically test motor invariance, neither in behavior nor in neural activity. Although one can infer from their figures that the second reach, which was at a different location from the first reach, has a similar "reach angle" tuning (their Fig. 5), whether this was generally the case was neither tested nor quantified (as it was not the focus of their study).
- Second, their study does not test for categorical structure in behavior or neural activity. If anything, their study implies continuously varying encoding of reach angle in one of their key analyses (their Fig. 5), thus a lack of categorical structure.
- Third, their study does not test reuse of reaches in the context of generalization. Indeed, each of the two-segment reach sequences was highly practiced. This makes it unclear how their finding of reuse of reach representations relates to compositional generalization.

For these reasons, we believe that the Zimnik study — and prior studies similarly finding evidence for reuse in M1 (e.g., see our references in lines 637 to 638) — do not directly address the question of action symbols.

We also note that we did assess M1 and PMd in our study and found that neither exhibited action-symbol properties similar to PMv.

Summarizing this argument, in the Discussion, we have a section stating that prior studies have shown evidence for motor primitives in M1 (where "motor primitives" refers to reused motor elements that don't necessarily have symbolic properties). We realize that we should have cited Zimnik and Churchland in this section. We have revised the text.

- "An open question is whether motor primitives are encoded in the brain (see evidence in M1 **[reference added here]**)" (lines 637 to 638).

The reviewer suggests that *"[the authors did not analyze] how exactly the primitives are represented in PMv. Perhaps the dynamic features in the planning period could be explored further, rather than simply having them described as distinct trajectories."* We agree that this is an interesting suggestion, with our understanding that it is a similar suggestion to the one in a previous comment, of assessing contextual effects in neural dynamics (see our response above). Further, we wish to highlight all the extensive investigations that we *do pursue* regarding "how exactly the primitives are represented in PMv", with the most important ones being invariance (Fig. 4), categorical structure (Fig. 5), recombination (Fig. 6), visual and motor kinematic encoding (Extended Data Figs. 15, 16).

The reviewer also states that we have not investigated the "*mechanisms of recombination/relation"*, but previously did acknowledge that this is not a problem: *"I agree that simultaneously addressing action symbol representation and syntactic structure may be overly ambitious for a single paper, …Thus, focusing solely on action symbols is justifiable".*

For all these reasons, we would suggest small but important revisions to the reviewer's "take-home message" of our paper, in the statement: "*the novelty is to some extent compromised, when the take-home message can be summarized as "reuse of invariantly represented movements".* We suggest that the empirical take-home is more like: "*reuse of invariantly and categorically represented movements, in recombination, to perform a large variety of complex sequences, including novel ones*", and that there is a conceptual one that none of these earlier studies have reached: "evidence for an action symbol code specifically in one brain region, PMv"

Finally, we do thank the reviewer for very thoughtful and thorough suggestions. While we acknowledge that there may still remain some disagreements, we hope these disagreements productively inspire future research. We also hope that our revisions have sufficiently addressed the reviewer's reservations to the point of allowing us to conclude that our results provide evidence for the discovery of a neural representation of action symbols, localized to PMv.

[redacted]

Referees' comments:

Referee #1 (Remarks to the Author):

We thank the authors for their response. However, we continue to believe that there is still a need to resolve an open question regarding the emphasis placed on "ambiguity" in the stimuli used to cue action symbol representations. This ambiguity is precisely what poses the "input parsing problem" raised in earlier rounds of review, concerning the centrality of visual processing in thinking about action symbol representations.We suggest two potential ways to address our concerns.

If the authors believe that the problem of visually decomposing ambiguous stimuli such as the glyphs used in the present studies is not central for supporting action symbol representations, but is mostly useful from a methodological perspective, then we think it would be appropriate to soften claims about the novelty and centrality of their experimental paradigm in investigating action symbol representations. There needs to be strong justifications for why simpler task paradigms (refer to sound/light cueing examples from earlier correspondences) would not test for the existence of action symbols and the need for the drawing-based paradigm.

If, on the other hand, the authors believe that what is truly key about the drawing task is the fact that the glyphs are ambiguous with respect to what actions must be performed (i.e., impose “minimal instruction from the environment”) and that many potential ways of drawing the glyph can be conceived (each recruiting different action symbols), then the “input parsing problem” feels deeply central to the phenomena under study. That is, the action(s) used to produce the drawing are reliant on how the agent discretizes a static image of a glyph into segments, each of which must be reproduced using discrete actions. The authors should emphasize the importance of the problem of visually parsing these shapes as a core prerequisite for action symbol representations. To be clear, we are not saying that PMv is the site of the parsing (the results of the experiments reported in Extended Data Fig. 15 help dissociate action plans from perception), but that a key property of learned action symbols is that they require parsing otherwise ambiguous sensory inputs. These operations might ostensibly be related to operations in sensory processing regions upstream of PMv.

We thank the reviewer for allowing us to further clarify what exactly is important about ambiguity in the stimuli used in our task. Our position is aligned with the reviewer’s option #1, that the use of ambiguous stimuli is important mainly from a methodological perspective, a major reason being that it is useful for assessing the nature of prior knowledge. This has been the position we have been attempting to take

over the last two rounds of review, including through various edits to the manuscript. To clarify how we have presented our position, we collect here the relevant statements from the manuscript and from previous responses to reviewers:

- In the manuscript, whenever stimulus ambiguity is mentioned, it is either implied or explicitly stated that we use ambiguity as a way to probe the nature of prior knowledge, and it is not stated that we use ambiguity because "input parsing" is essential to the role of action symbols. Specifically:
    - In the paragraph explaining how this task represents a new approach to study compositional generalization, we stated, in lines 620 to 621: "Second, images were generally ambiguous, which allowed us to probe prior knowledge."
    - In the results section on recombination, we noted that "...and characters had ambiguity in being consistent with multiple possible interpretations in terms of shape components" (lines 269 to 270), and then implied that this ambiguity is useful because it allowed us to probe prior knowledge by asking whether subjects reused their own primitives: "Given the same image, the two subjects produced different drawings (Fig. 2I), raising the possibility that subjects reused their primitives" (lines 279 to 280).
    - In the results section on categorical structure, ambiguity was also framed around it being a useful experimental method for finding evidence of categorical structure: "for the ambiguous image at the category boundary (morph v), trajectories diverged towards these two states depending on whether the subject was planning to draw primitive 1 or 2")" (lines 420 to 422).
- Also, in the manuscript, all implicit or explicit definitions of action symbols and compositional generalization emphasize the question of how the brain represents and recombines discrete units, rather than how the brain parses ambiguous stimuli. Specifically:
    - "generation of novel ideas and behaviors [is hypothesized to rely on] internal representations of discrete units, or symbols, and processes that recombine them into a large set of possible composite representations". (lines 8 to 9)
    - "...action symbols—recombinable representations of discrete units of motor behavior…" (line 12)
    - "An influential hypothesis is that generalization depends on an internal representation of discrete units (symbols) that can be recombined into composite representations, in a process called compositional generalization" (lines 51 to 53)
    - "...symbols are discrete representational units that are systematically recombined…" (line 66)
    - "This task involves generating novel, goal-directed actions, an ability hypothesized to involve symbolic representations in the form of discrete units of motor behavior (action symbols) that are recombined into sequences" (lines 92 to 94).
- Finally, in our previous two responses to reviewers, we also agreed with the reviewer's option #1. Specifically:
    - In our response to the second round of reviews:
        - "We did not intend to mean that action symbols are only induced in situations with ambiguity. Instead, we agree with the reviewer that even in situations with no ambiguity but with internally constructed and goal-directed movements, action symbols may very well be involved. The point we intended to make was that ambiguity is a useful feature in tasks testing the existence, nature, and neural encoding of action symbols in compositional generalization." (lines 60 to 65).

        - “As a consequence, we also do not view input parsing as fundamental to explaining action symbols and compositional generalization. A further consequence is that we do agree with the reviewer that the “GREEN RED” cue example may very well involve action symbols.” (lines 97 to 99).
    - In our response to the first round of reviews:
        - “In such a task [involving red/green cues], one might also expect action symbols.”
        - “In short, an action symbol is an internal representation that supports compositional generalization. They can sometimes be directly tied one-to-one to particular visual cues, but this is not necessary.” The point here is that action symbols indeed sometimes can (but will not always) be driven directly (“one-to-one”) by visual cues isomorphic to the actions, a situation that does not involve ambiguous stimuli.

To make it more clear that we view ambiguity as a useful methodological feature, we have added a few clarifications to the manuscript.
- We modified “Our study introduces a task to study compositional generalization. We highlight three critical features” (line 613) to “Our study introduces a task for studying compositional generalization. We highlight three methodological features advantageous for studying compositional generalization.”
- We modified “Second, images were generally ambiguous, which allowed us to probe prior knowledge.” (lines 620 to 621) to “Second, images were often ambiguous, which was useful for probing the nature of each subject’s prior knowledge”.

Thus, our statements in responses to reviewers and in the manuscript have emphasized that ambiguity is a useful methodological feature, consistent with the reviewer’s option #1.

Our agreement with the reviewer’s option #1 raises another concern, as stated by the reviewer:
- *“If the authors believe that the problem of visually decomposing ambiguous stimuli … is mostly useful from a methodological perspective, then we think it would be appropriate to soften claims about the novelty and centrality of their experimental paradigm in investigating action symbol representations. There needs to be strong justifications for why simpler task paradigms (refer to sound/light cueing examples from earlier correspondences) would not test for the existence of action symbols and the need for the drawing-based paradigm”.*

First, we believe that the above revisions to the manuscript help soften our claims about novelty and centrality by clarifying that the key advantages of this task that we list are due to methodological innovations.

We also wish to clarify what seems like a possible misunderstanding—we are not arguing that simpler task paradigms do not recruit action symbols, nor that simpler task paradigms cannot possibly be used to study action symbols. Rather, we are arguing that there are properties of this drawing task paradigm that are advantageous for identifying and studying the nature of action symbols. Indeed, in both rounds of review, we acknowledged that other task paradigms—including the sound/light cuing example, and the object manipulation example—may involve action symbols. Specifically:
- In our response to the second round of reviews:
    - “As a consequence, we also do not view input parsing as fundamental to explaining action symbols and compositional generalization. A further consequence is that we do

agree with the reviewer that the “GREEN RED” cue example may very well involve action symbols.” (lines 97 to 99).

- In our response to the first round of reviews:
  - “In such a task [involving red/green cues], one might also expect action symbols.”
- And in the manuscript we have emphasized that prior behavioral and neural findings may indeed reflect action symbols, an interesting question for future studies:
  - “Our study’s criteria of invariance, categorical structure, and recombination may be useful in future studies, including in reassessing prior findings of invariant activity in cognitive tasks as symbols.” (lines 564 to 566)
  - “Our findings raise the possibility that PMv’s involvement in diverse motor behaviors—including speech—may reflect diverse kinds of action symbols” (lines 593 to 594).
  - “For both motor primitives and syllables, future studies may address whether they reflect action symbols.” (lines 649 to 650).

Thus, our statements in both responses to reviewers and the manuscript do not argue that “simpler” tasks cannot involve action symbols.

This discussion about stimulus ambiguity also highlights a separate but related important point about the advantages of this drawing task, in that it captures a critical aspect of complex naturalistic behavior, which is the need to make decisions in situations where sensory inputs, task specifications (rewards, task structure, etc), and specific prior experiences do not uniquely determine what behaviors to perform and how to generalize to new situations—thus requiring internal decisions and computations involving a complex mixture of perceptual processing, decision-making, and efficient planning, all constrained by structured prior knowledge. Thus, what makes symbols useful and important (among other aspects) is that—rather than being one-to-one linked to sensory inputs or rewarded behavior—they are reusable units of knowledge that are flexibly deployed and recombined by the brain's internal computations. Our drawing task helped us identify a neural representation of action symbols. It also highlights and lets us study how symbols are engaged to enable complex generalization behavior—it leaves open the question of how the brain decides which action symbols and combinations of symbols to deploy, a process that likely involves visual perception, efficient action planning, and computations involving multiple brain areas besides PMv. This drawing task is well-suited for future studies to address these questions.

- We have revised the manuscript to highlight these points, in lines 623 to 630: “Together, these task features (free choice, stimulus ambiguity, and parametric stimulus design) relate to a critical aspect of complex naturalistic behavior—the need to generalize to situations where behavior is not uniquely determined by sensory inputs and specific prior experiences, but instead depends on internal decision-making computations involving multiple interacting processes, including perception, inference, and efficient planning, all constrained by structured prior knowledge, including of symbols and how they can be flexibly recombined. Thus, we established a task paradigm that allowed us to identify action symbols, and which, in future work, allows studying how multi-area neural processes underlie the internal computations that map from complex sensory input to structured symbolic behavior.”

Overall, we hope we have clarified our position that stimulus ambiguity is a useful methodological feature of tasks studying action symbols and compositional generalization, and that we have presented

this idea—and more broadly, the reasons we believe our task paradigm is a novel and useful way to study compositional generalization—in a fair and well-reasoned manner.

We would also gently suggest some additional close editing passes over the manuscript to ensure that the argument narrative flows as intended.

We thank the reviewer for this suggestion, and have made various edits to tighten the narrative (see tracked edits).

Referee #2 (Remarks to the Author):

I co-reviewed this manuscript with one of the reviewers who provided the listed reports.

Referee #4 (Remarks to the Author):

All my comments have been addressed. I would like to congratulate the authors on a very nice piece of work.

Thank you!
